# A Review of Zoonotic Infection Risks Associated with the Wild Meat Trade in Malaysia

**DOI:** 10.1007/s10393-017-1229-x

**Published:** 2017-03-22

**Authors:** Jennifer Caroline Cantlay, Daniel J. Ingram, Anna L. Meredith

**Affiliations:** 1 The Royal (Dick) School of Veterinary Studies and The Roslin Institute, The University of Edinburgh, Easter Bush Campus, Roslin, Midlothian, EH25 9RG UK; 2 Independent Researcher, Unit 6301, No 1, Lane 600, Central Yincheng Road, Pudong District, Shanghai, 200120 People’s Republic of China; 3 School of Life Sciences, University of Sussex, Brighton, BN1 9GQ UK

**Keywords:** wild meat, zoonotic pathogens, infection risk, public health, Southeast Asia

## Abstract

The overhunting of wildlife for food and commercial gain presents a major threat to biodiversity in tropical forests and poses health risks to humans from contact with wild animals. Using a recent survey of wildlife offered at wild meat markets in Malaysia as a basis, we review the literature to determine the potential zoonotic infection risks from hunting, butchering and consuming the species offered. We also determine which taxa potentially host the highest number of pathogens and discuss the significant disease risks from traded wildlife, considering how cultural practices influence zoonotic transmission. We identify 51 zoonotic pathogens (16 viruses, 19 bacteria and 16 parasites) potentially hosted by wildlife and describe the human health risks. The *Suidae* and the *Cervidae* families potentially host the highest number of pathogens. We conclude that there are substantial gaps in our knowledge of zoonotic pathogens and recommend performing microbial food safety risk assessments to assess the hazards of wild meat consumption. Overall, there may be considerable zoonotic risks to people involved in the hunting, butchering or consumption of wild meat in Southeast Asia, and these should be considered in public health strategies.

**Electronic supplementary material:**

The online version of this article (doi:10.1007/s10393-017-1229-x) contains supplementary material, which is available to authorized users.

## Introduction

Globally, one of the most significant threats to wildlife is the overhunting of species for food and commercial gain (Schipper et al. 2008; Maxwell et al. 2016), which is prevalent in the Amazon (Peres 2000), West and Central Africa (Abernethy et al. 2013; Ingram et al. 2015) and Southeast Asia (Bennett et al. 2000; Scheffers et al. 2012; Luskin et al. 2014). The large quantity of wildlife harvested is highlighted in the literature; for example, one study estimated the annual wild meat harvest in the Malaysian state of Sarawak at 23,500 tonnes (Bennett 2002). The increased commercialisation of the wildlife trade facilitates the supply of wild meat to urban consumers (Milner-Gulland and Bennett 2003) and international markets (Chaber et al. 2010). This leads to greater movement of species that increases the likelihood of zoonotic pathogens being translocated, thus presenting health risks to human populations worldwide (Marano et al. 2007). Anthropogenic activities, including the global wildlife trade, have been linked to the rise in emerging infectious diseases (EIDs) (Karesh et al. 2007), and whilst the contribution from the wild meat trade is unknown, its involvement in zoonotic spillovers to humans has been recognised in some countries such as Côte d’Ivoire (Ayouba et al. 2013) and Cameroon (Pernet et al. 2014). “One Health” research (Atlas et al. 2010) synthesises this information and uses collaborative interdisciplinary approaches to improve understanding of zoonotic disease epidemiology in relation to human activities, such as wildlife hunting (Daszak et al. 2007).

People who are involved in wildlife hunting, butchering and consumption risk transmission of infection from their close contact (e.g. transcutaneous, mucosal routes) with live and dead animals or via contaminative routes (e.g. faeces, fomites). Zoonotic infections from hunting are well documented, such as an Ebola disease outbreak related to handling infected chimpanzee, gorilla and duiker carcasses (Leroy et al. 2004) and brucellosis in Australian hunters of wild boar (Eales et al. 2010). Foodborne infections from wild meat consumption have been reported globally, for example, Hepatitis E from raw or undercooked venison in Japan (Matsuda et al. 2003; Tei et al. 2003) and trichinellosis from wild boar meat in France (De Bruyne et al. 2006).

Whilst numerous studies have investigated the zoonotic disease risks from the trade of wild meat in Africa (Wolfe et al. 2005; Kamins et al. 2015), significantly less attention has been focused on Southeast Asia. In this region, many people consume a great variety of wildlife due to their cultural practices and beliefs. The demand for species valued as a delicacy, such as Sumatran serow meat in Malaysia (Shepherd and Krishnasamy 2014), or used for traditional medicine, including Asiatic softshell turtles in soup (Sharma 1999), has led to greater commercialisation of the trade within Southeast Asia (Scheffers et al. 2012; Shepherd and Krishnasamy 2014), which increases risks for human health. Since the wildlife trade distribution networks enable the regional movement of animals, this facilitates cross-species transmission of pathogens due to the mixing of numerous species from different sources in combination with the close proximity between wildlife and humans (Karesh et al. 2005). The importance of understanding how these networks influence zoonotic infection between species was illustrated by the spread of severe acute respiratory syndrome (SARS)-associated coronavirus from bats to civets to humans (Li et al. 2005c).

This aim of this review is to fill the gap in knowledge about Southeast Asia by evaluating published research to determine the potential zoonotic infection risks to humans from hunting, butchering and consumption of wildlife, using the wild meat trade in Malaysia as a case study.

## Methods

The taxa sold as wild meat in Malaysia were identified from a survey of wild meat establishments (restaurants, roadside stalls and markets) across Peninsular Malaysia, Sabah and Sarawak, conducted by TRAFFIC (Caillabet et al. (Unpublished). The species identified in this survey (Table 1) were used to categorise the potential zoonotic viral, bacterial and parasitic pathogens in wildlife hosts.

**Table 1:** Wildlife Species Identified in Surveyed Establishments Across Malaysia Listed in Order of Decreasing Availability.

Species
Deer spp. (e.g. *Muntiacus muntjak* and *Rusa unicolor*)
Sunda Bearded Pig (*Sus barbatus*)
Eurasian Wild Pig (*Sus scrofa*)
Squirrel spp. (not specified)
Civet spp. (e.g. *Paradoxurus hermaphroditus* and *Viverra tangalunga*)
Softshell Turtle (e.g. *Amyda cartilaginea* and *Dogania subplana*)
Sumatran Serow (*Capricornis sumatraensis*)
Flying Fox spp. (e.g. *Pteropus vampyrus* and *P. hypomelanus*)
Porcupine spp. (e.g. *Hystrix brachyura*)
Reticulated Python (*Python reticulatus*)
Water Monitor Lizard (*Varanus salvator*)
Estuarine Crocodile (*Crocodylus porosus*)
Sun Bear (*Helarctos malayanus*)
Long-tailed Macaque (*Macaca fascicularis*)
Sunda Pangolin (*Manis javanica*)
Tiger (*Panthera tigris jacksoni*)
Red Junglefowl (*Gallus gallus*)
Leopard (*Panthera pardus*)
Asian Elephant (*Elephas maximus*)
Monocled Cobra (*Naja kaouthia*)
Tortoise spp. (not specified)

Between July 2014 and February 2015, we conducted a literature review of publications using online databases Google Scholar and Web of Science, with further information collected from the disease reporting database, ProMed. The initial search used all possible combinations of key words relating to the traded species (e.g. “tiger” or “*Panthera*”), infectious disease terminology (including “zoonotic”, “zoonoses”, “infection” and “infectious”) and three pathogen categories (including “virus”, “viral”, “bacteria”, “bacterial”, “parasite” and “parasitic”). Different combinations of the key words were linked together (e.g. “tiger” AND “zoonotic” AND “virus”) to search for information about zoonotic pathogens circulating in wildlife hosts. Specific inclusion criteria utilised surveys (serological and faecal sampling) and disease investigations (post mortem examinations) of free-ranging and captive wild animal populations for pathogens, with negative results excluded. In some cases, insufficient data about the traded species necessitated the use of research from other species within the same taxonomic family or order. Due to the lack of data on sun bears, the search was expanded to other *Ursidae* species. This approach assumes that taxonomically related hosts would share similar pathogens due to their phylogeny (Davies and Pedersen 2008). We excluded vector-borne pathogens from this review because of their indirect transmission route to humans, which we considered to be less relevant for wildlife hunters and consumers as an immediate route of zoonotic transmission than handling and consuming carcases.

A subsequent search was conducted to find evidence for zoonotic infections in humans from wildlife. It combined the word “human” with key words relating to the zoonotic pathogens identified in the initial search (e.g. “Bacillus anthracis”) or associated human disease (e.g. “anthrax”) and the wildlife host (e.g. “deer”). For example, “human” AND “bacillus anthracis” AND “deer” or “human” AND “anthrax” AND “deer”. We included disease case reports (occupational exposure to wild animals) and serological surveys of some human populations (indigenous tribes with hunting traditions), which provided information on the transmission routes and infection risks from the hunting, butchering and consumption of wildlife.

There was no limitation placed on the date of publication for the searches conducted. We examined publications and databases globally for relevant zoonotic information, but excluded pathogens geographically distributed outside of Asia. Additional references were identified by searching the reference lists of the papers that were obtained from the literature search.

## Results

**Figure 1:**
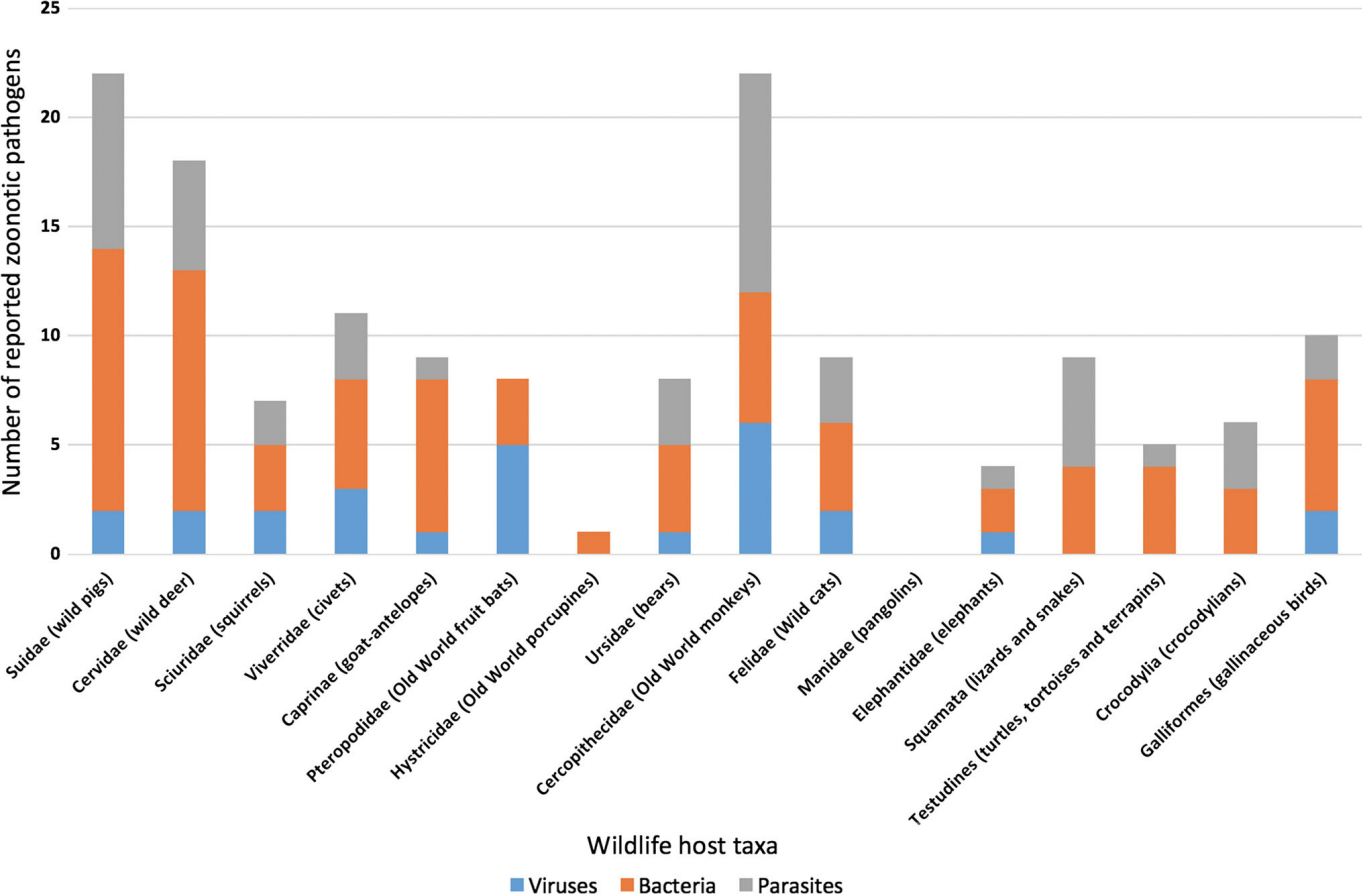
Total numbers of viral, bacterial and parasitic pathogens reported in traded wildlife taxa.

In total, 475 references were found that met the inclusion criteria to provide information on the viruses, bacteria and parasites that could potentially be hosted by the wild animals for sale in Malaysia (Tables 2, 3, 4, A1–3). Old world monkeys (Family: *Cercopithecidae*) and wild pigs (Family: *Suidae*) were found to host the greatest combined numbers of zoonotic pathogens (Figure 1). Potential transmission routes of the zoonotic pathogens were associated with wildlife hunting (capture and handling of animals), butchering (evisceration, skinning and cutting of carcasses) and consumption of the traded taxa (Table 5).

**Table 2:** Potential Zoonotic Viral Pathogens from Wildlife Involved in the Wild Meat Trade.

Virus	Wildlife host (taxonomic order or family)							
	Suidae	Cervidae	Sciuridae	Viverridae	Caprinae	Pteropodidae	Hystricidae	Ursidae
Avian paramyxovirus-1								
Cercopithecine herpesvirus-1								
Cowpox virus (Orthopoxvirus)								
Ebola virus subtype Reston						**X**		
Hepatitis E virus	**X**	**X**						
Highly pathogenic avian influenza virus				**X**				
Lymphocytic choriomeningitis virus			**X**					
Nipah virus						**X**		
Orf virus (Parapoxvirus)		**X**			**X**			
Rabies virus and related Lyssaviruses			**X**	**X**		**X**		**X**
Reoviruses (e.g. Melaka virus)						**X**		
SARS Coronavirus				**X**		**X**		
Simian foamy virus								
Simian type D retrovirus								
Simian virus 40								
Swine influenza virus	**X**							

Virus	Wildlife host (taxonomic order or family)							
	Cercopithecidae	Felidae	Manidae	Elephantidae	Squamata	Testudines	Crocodylia	Galliformes
Avian paramyxovirus-1								**X**
Cercopithecine herpesvirus-1	**X**							
Cowpox virus (Orthopoxvirus)				**X**				
Ebola virus subtype Reston	**X**							
Hepatitis E virus								
Highly pathogenic avian influenza virus		**X**						**X**
Lymphocytic choriomeningitis virus								
Nipah virus								
Orf virus (Parapoxvirus)								
Rabies virus and related Lyssaviruses	**X**	**X**						
Reoviruses (e.g. Melaka virus)								
SARS* Coronavirus								
Simian foamy virus	**X**							
Simian type D retrovirus	**X**							
Simian virus 40	**X**							
Swine influenza virus

**Table 3:** Potential Zoonotic Bacterial Pathogens from Wildlife Involved in the Wild Meat Trade.

Bacteria	Wildlife host (taxonomic order or family)							
	Suidae	Cervidae	Sciuridae	Viverridae	Caprinae	Pteropodidae	Hystricidae	Ursidae
*Bacillus anthracis*	**X**	**X**						
*Bartonella henselae*				**X**				
*Brucella* spp.	**X**	**X**			**X**			**X**
*Campylobacter* spp.	**X**	**X**		**X**	**X**			
*Chlamydophila* spp.	**X**	**X**			**X**			
*Dermatophilus congolensis*								
*Edwardsiella tarda*								
*Erysipelothrix rhusiopathiae*	**X**	**X**						
*Escherichia coli* (shiga-toxin producing)	**X**	**X**			**X**			
*Francisella tularensis*	**X**		**X**					**X**
*Leptospira* spp.	**X**	**X**	**X**		**X**	**X**	**X**	**X**
*Mycobacterium tuberculosis* complex	**X**	**X**			**X**			
Other *Mycobacterium* spp.								
*Pasteurella* spp.								
*Salmonella* spp.	**X**	**X**		**X**	**X**	**X**		
*Shigella* spp.								
*Streptococcus* spp.	**X**			**X**				
*Yersinia pestis*		**X**	**X**					**X**
Other *Yersinia* spp.	**X**	**X**		**X**		**X**		

Bacteria	Wildlife host (taxonomic order or family)							
	Cercopithecidae	Felidae	Manidae	Elephantidae	Squamata	Testudines	Crocodylia	Galliformes
*Bacillus anthracis*				**X**				
*Bartonella henselae*		**X**						
*Brucella* spp.								
*Campylobacter* spp.	**X**				**X**	**X**		**X**
*Chlamydophila* spp.								**X**
*Dermatophilus congolensis*					**X**	**X**	**X**	
*Edwardsiella tarda*					**X**	**X**	**X**	
*Erysipelothrix rhusiopathiae*								**X**
*Escherichia coli* (shiga-toxin producing)								
*Francisella tularensis*								
*Leptospira* spp.	**X**	**X**						
*Mycobacterium tuberculosis* complex	**X**			**X**				
Other *Mycobacterium* spp.	**X**							**X**
*Pasteurella* spp.		**X**						
*Salmonella* spp.	**X**				**X**	**X**	**X**	**X**
*Shigella* spp.	**X**							
*Streptococcus* spp.								
*Yersinia pestis*		**X**						
Other *Yersinia* spp.								**X**

**Table 4:** Potential Parasitic Pathogens from Wildlife Involved in the Wild Meat Trade.

Parasite	Wildlife host (taxonomic order or family)							
	Suidae	Cervidae	Sciuridae	Viverridae	Caprinae	Pteropodidae	Hystricidae	Ursidae
*Ancyclostoma* spp.								
*Anisakidae* spp.								
*Balantidium coli*	**X**							
*Cryptosporidium* spp.	**X**	**X**						
*Enantomoeba histolytica*								
*Giardia* spp.	**X**	**X**						
*Gnathostoma* spp.				**X**				
*Oesophagostomum* spp.								
*Pentastomidia* spp.								
*Sarcocystis* spp.		**X**	**X**	**X**				**X**
*Spirometra* spp.	**X**							
*Strongyloides* spp.								
*Taenia* spp.	**X**							
*Toxoplasma gondii*	**X**	**X**	**X**	**X**	**X**			**X**
*Trichinella* spp.	**X**	**X**						**X**
*Trichuris* spp.	**X**							

Parasite	Wildlife host (taxonomic order or family)							
	Cercopithecidae	Felidae	Manidae	Elephantidae	Squamata	Testudines	Crocodylia	Galliformes
*Ancyclostoma* spp.	**X**							
*Anisakidae* spp.							**X**	
*Balantidium coli*	**X**							
*Cryptosporidium* spp.	**X**	**X**		**X**				**X**
*Enantomoeba histolytica*	**X**							
*Giardia* spp.	**X**							
*Gnathostoma* spp.					**X**			
*Oesophagostomum* spp.	**X**							
*Pentastomidia* spp.					**X**	**X**	**X**	
*Sarcocystis* spp.	**X**				**X**			**X**
*Spirometra* spp.					**X**			
*Strongyloides* spp.	**X**							
*Taenia* spp.								
*Toxoplasma gondii*	**X**	**X**						
*Trichinella* spp.		**X**			**X**		**X**	
*Trichuris* spp.	**X**

**Table 5:** Transmission Risks from the Potential Zoonotic Pathogens.

Type of pathogen	Pathogen species	Human disease description	Potential transmission route from wildlife to human	Potential zoonotic risk from hunting, butchering or consumption	Referenced evidence of zoonotic infection to humans from wildlife taxa
Virus	*Avian paramyxovirus-1* (Newcastle disease)	Conjunctivitis or influenza-like disease	Contact with large amounts of virus from infected birds or their carcasses e.g. inhalation	Hunting Butchering	
	*Cercopithecine herpesvirus-1*	Herpes B virus disease	Transcutaneous: via animal bites or scratches Non-bite exposure: via mucous membranes or damaged skin	Hunting Butchering	*Cercopithecidae*: (Holmes et al. 1990; CDC 1987; CDC 1998; Huff and Barry 2003) USA; (Weigler 1992) USA and UK
	*Cowpox virus (Orthopoxvirus)*	Cowpox	Transcutaneous: via animal bites, scratches or damaged skin	Hunting Butchering	*Elephantidae*: (Hemmer et al. 2010; Kurth et al. 2008) Germany
	*Ebola virus* (subtype Reston)	Ebola haemorrhagic fever	Contact with infected animals, body fluids and tissues	Hunting Butchering	*Cercopithecidae*: (Miranda et al. 1999; Morikawa et al. 2007) USA and Philippines
	*Hepatitis E virus*	Hepatitis E	Foodborne Faeco-oral? Direct contact with infected animal blood?	Consumption Hunting? Butchering?	*Suidae*: (Li et al. 2005b; Masuda et al. 2005; Matsuda et al. 2003; Toyoda et al. 2008) Japan; (Wichmann et al. 2008) Germany. *Cervidae*: (Takahashi et al. 2004; Tei et al. 2003; Tei et al. 2004) Japan
	*Highly pathogenic avian influenza virus*	Avian influenza	Contact with infected respiratory secretions Ingestion of blood or undercooked meat? Faeco-oral?	Hunting Butchering Consumption?	
	*Lymphocytic choriomeningitis virus*	Lymphocytic choriomeningitis	Transcutaneous: via animal bites Contact with infected animal excretions and secretions	Hunting Butchering	
	*Nipah virus*	Nipah virus infection	Ingestion of virus-contaminated food products Contact with infected urine or saliva or tissues	Consumption Hunting Butchering	*Pteropodidae*: (Epstein et al. 2008) India; (Luby et al. 2006; Luby et al. 2009) Bangladesh
	*Orf virus (Parapoxvirus)*	Contagious ecthyma	Transcutaneous: via damaged skin or wounds	Hunting Butchering	*Cervidae*: (Kuhl et al. 2003; Roess et al. 2010; Smith et al. 1991) USA
	*Rabies virus* and related *Lyssaviruses*	Rabies and rabies-related disease	Transcutaneous: via animal bites and scratches Non-bite exposure: via mucous membranes or damaged skin	Hunting Butchering	*Sciuridae*: (Kumari et al. 2014) India; (ProMED-mail 2014b) Costa Rica *Viverridae*: (ProMED-mail 2009) Tanzania *Pteropodidae*: (Hanna et al. 2000; Samaratunga et al. 1998; Warrilow et al. 2002; ProMED-mail 2014a) Australia *Cercopithecidae*: (Favoretto et al. 2001) Brazil; (Summer et al. 2004) India *Felidae*: (Pandit 1950) India
	*Reoviruses* (e.g. *Melaka virus, Pulau virus)*	Acute respiratory disease	Direct transmission from bat to human occurs via close contact?	Hunting? Butchering?	*Pteropodidae*: (Chua et al. 2007) Malaysia
	SARS* Coronavirus	SARS	Mucosal transmission: contact with virus-infected respiratory droplets Indirect transmission via virus-contaminated fomites	Hunting Butchering	*Viverridae*: (Bell et al. 2004; Wang et al. 2005; Xu et al. 2004) China
	*Simian foamy virus*	Simian foamy virus infection	Transcutaneous and mucosal: via animal bites, scratches and saliva splashes	Hunting Butchering	*Cercopithecidae*: (Brooks et al. 2002) Canada; (Huang et al. 2012) China; (Jones-Engel et al. 2005) Indonesia; (Jones-Engel et al. 2008) several Asian countries; (Schweizer et al. 1997) Germany; (Wolfe et al. 2004) Cameroon
	*Simian type D retrovirus*	Persistently seropositive humans without disease	Transcutaneous and mucosal: via animal bites, scratches, saliva splashes?	Hunting? Butchering?	*Cercopithecidae*: (Lerche et al. 2001) USA
	*Simian virus 40*	Role in human cancers?	Transcutaneous and mucosal: via animal bites, scratches and saliva splashes	Hunting Butchering	*Cercopithecidae*: (Engels et al. 2004) North America; (Shah 1972) India
	*Swine influenza virus*	Swine influenza	Contact with infected respiratory secretions	Hunting Butchering	
Bacteria	*Bacillus anthracis*	Anthrax	Foodborne Transcutaneous: contact with contaminated carcasses and animal products Inhalation of spores	Consumption Butchering	*Suidae*: (ProMED-mail 2011) India *Cervidae*: (Ichhpujani et al. 2004) India; (ProMED-mail 2001) USA; (Fasanella et al. 2007) Italy
	*Bartonella henselae*	Cat scratch disease	Transcutaneous via animal bites and scratches	Hunting	*Viverridae*: (Miyazaki et al. 2001) Japan
	*Brucella* spp.	Brucellosis	Foodborne Transcutaneous and mucosal: contact with infected bodily fluids or tissues	Consumption Hunting Butchering	*Suidae*: (Carrington et al. 2012; Giurgiutiu et al. 2009; Starnes et al. 2004) USA; (Eales et al. 2010; Massey et al. 2011; Robson et al. 1993) Australia; (Garin-Bastuji et al. 2006) France *Cervidae*: (Brody et al. 1966) Alaska, USA; (Chan et al. 1989) Arctic region; (Forbes 1991) Canada; (Meyer 1966) Alaska, Canada and Russia
	*Campylobacter* spp.	Campylobacter enteritis	Foodborne Faeco-oral	Consumption Hunting Butchering	*Squamata*: (Patrick et al. 2013) USA *Testudines*: (Patrick et al. 2013; Tu et al. 2004) USA
	*Chlamydophilia* spp.	Chlamydiosis	Transcutaneous and aerogenous: contact with infected secretions or excretions	Hunting Butchering	
		Psittacosis (from birds)	Inhalation of infected respiratory secretions or dried faeces	Hunting Butchering	
	*Dermatophilus congolensis*	Dermatophilosis	Transcutaneous: direct contact with infected lesions	Hunting Butchering	
	*Edwardsiella tarda*	Edwardsiellosis	Foodborne Faeco-oral Transcutaneous: via wound	Consumption Hunting Butchering	*Testudines*: (Nagel et al. 1982) USA
	*Erysipelothrix rhusiopathiae*	Erysipeloid	Foodborne Transcutaneous: direct contact with infected animal products via damaged skin/wounds	Consumption Butchering	*Suidae*: (Addidle et al. 2009) New Zealand. *Galliformes*: (Mutalib et al. 1995) USA
	*Escherichia coli (Shiga-toxin producing)* spp.	Enterohaemorrhagic *E. coli* infections	Foodborne Faecal-oral	Consumption Hunting Butchering	*Cervidae*: (Keene et al. 1997; Rabatsky-Ehr et al. 2002; Rounds et al. 2012) USA; (Nagano et al. 2004) Japan
	*Francisella tularensis*	Tularemia	Foodborne Transcutaneous or mucosal: direct contact with infected animals Inhalation of aerosolised bacteria	Consumption Hunting Butchering	*Suidae*: (Deutz et al. 2002) Austria; (Esmaeili et al. 2014) Iran *Sciuridae*: (Bow and Brown 1946) Canada; (Magee et al. 1989) USA *Ursidae*: (Chase et al. 1980) USA
	*Leptospira* spp.	Leptospirosis	Foodborne: urine-contaminated meat Transcutaneous or mucosal: contact with infected urine	Consumption Hunting Butchering	*Cervidae*: (Brown 2005) New Zealand *Sciuridae*: (Diesch et al. 1967) USA; (Masuzawa et al. 2006) Japan *Pteropodidae*: (Vashi et al. 2010) USA *Ursidae*: (Anderson et al. 1978) USA
	*Mycobacterium tuberculosis complex (M. tuberculosis and M. bovis)*	Tuberculosis	Foodborne Transcutaneous: direct contact via damaged skin/wounds Inhalation of aerosolised bacteria	Consumption Hunting Butchering	*Cervidae*: (Baker et al. 2006) New Zealand; (Fanning and Edwards 1991; Liss et al. 1993; Nation et al. 1999) Canada; (Wilkins et al. 2003) (Wilkins et al. 2008) USA *Cercopithecidae*: (Une and Mori 2007) Japan *Elephantidae*: (Michalak et al. 1998; Murphree et al. 2011) USA
	Other *Mycobacterium* spp.	Mycobacteriosis	Inhalation or ingestion of aerosolised bacteria	Butchering	
	*Pasteurella* spp.	Pasteurellosis	Transcutaneous: via animal bites	Hunting	*Felidae*: (Capitini et al. 2002; Durazo and Lessenger 2006) USA; (Isotalo et al. 2000) Canada
	*Salmonella* spp.	Salmonellosis	Foodborne Faecal-oral Transcutaneous: via animal bites and scratches	Consumption Hunting Butchering	*Cervidae*: (Kuhn et al. 2011) Denmark; (Madar et al. 2012) Hawaii *Squamata*: (Bhatt et al. 1989; Kelly et al. 1995 ; Friedman et al. 1998; Corrente et al. 2006) USA *Testudines*: (Fukushima et al. 2008) Japan; (Harris et al. 2009) USA
	*Shigella* spp.	Shigellosis	Foodborne: faecal-contaminated meat Faeco-oral	Consumption Hunting Butchering	*Cercopithecidae*: (Kennedy et al. 1993) UK
	*Streptococcus* spp.	Streptococcosis	Transcutaneous: direct contact via damaged skin/wounds	Hunting Butchering	*Suidae*: (Dalsjö et al. 2014) Sweden; (Halaby et al. 2000) The Netherlands; (Rosenkranz et al. 2003) Germany
	*Yersinia pestis*	Plague	Transcutaneous or mucosal: contact with infected animals or carcasses Inhalation of aerosolised bacteria	Hunting Butchering	*Sciuridae*: (Li et al. 2005a) China
	Other *Yersinia* spp.	Yersiniosis	Foodborne Faeco-oral	Consumption Hunting Butchering	
Parasite	*Ancyclostoma* spp.	Cutaneous larva migrans	Transcutaneous: infective larvae that penetrate skin	Butchering	
	*Anisakidae* spp.	Anisakiasis	Foodborne: infective larvae in meat	Consumption	
	*Balantidium coli*	Balantidiasis	Foodborne: faecal-contaminated meat Faeco-oral: ingestion of cysts	Consumption Hunting Butchering	
	*Cryptosporidium* spp.	Cryptosporidiosis	Foodborne: faecal-contaminated meat Faeco-oral: ingestion of oocysts	Consumption Hunting Butchering	
	*Enantomoeba histolytica*	Amoebiasis	Foodborne: faecal-contaminated meat Faeco-oral: ingestion of cysts	Consumption Hunting Butchering	
	*Giardia* spp.	Giardiasis	Foodborne: faecal-contaminated meat Faeco-oral: ingestion of cysts	Consumption Hunting Butchering	
	*Gnathostoma* spp.	Gnathostomiasis	Foodborne: infective larvae in meat	Consumption	*Squamata:* (Akahane et al. 1998) Japan and Thailand; (Seguchi et al. 1995) Japan
	*Oesophagostomum* spp.	Oesophagostomiasis	Foodborne: faecal-contaminated meat Faeco-oral: ingestion of filariform larvae	Consumption Hunting Butchering	
	*Pentastomidia* spp.	Pentastomiasis	Foodborne: infective larvae in meat Faeco-oral: ingestion of eggs Direct contact with infected animal tissues and respiratory secretions	Consumption Hunting Butchering	*Squamata*: (Latif et al. 2011; Prathap et al. 1969) Malaysia; (Yao et al. 2008; Ye et al. 2013) China; (Yapo Ette et al. 2003) Ivory Coast
	*Sarcocystis* spp.	Sarcocystosis	Foodborne: infective sarcocysts in meat from intermediate host or faecal-contaminated meat from definitive host Faeco-oral: ingestion of oocysts from definitive host	Consumption Hunting Butchering	*Squamata*: (Lau et al. 2014; Tappe et al. 2013) Malaysia
	*Spirometra* spp.	Sparganosis	Foodborne: infective larvae in meat from the second intermediate host	Consumption	*Suidae*: (Tanaka et al. 1997) Japan *Squamata*: (Anantaphruti et al. 2011; Wiwanitkit 2005) Thailand; (Min 1990; Park et al. 2001) South Korea
	*Strongyloides* spp.	Strongyloidiasis	Transcutaneous or mucosal: infective larvae from faeces that penetrate skin or mucous membranes	Hunting Butchering	
	*Taenia* spp.	Taeniasis	Foodborne: infective larvae in meat /viscera from intermediate host	Consumption	*Suidae*: (Fan 1988; Fan et al. 1992) Taiwan
	*Toxoplasma gondii*	Toxoplasmosis	Foodborne: infective cysts in meat from the intermediate host or faecal-contaminated meat from definitive host Faeco-oral: ingestion of oocysts from definitive host	Consumption Hunting Butchering	*Suidae*: (Choi et al. 1997) South Korea *Cervidae*: (McDonald et al. 1990) Canada; (Ross et al. 2001; Sacks et al. 1983) USA *Sciuridae*: (Alvarado-Esquivel et al. 2008) Mexico *Felidae*: (Carme et al. 2009) French Guiana
	*Trichinella* spp.	Trichinellosis	Foodborne: infective cysts in meat	Consumption	*Suidae*: (Cui et al. 2011) China; (De Bruyne et al. 2006; Ranque et al. 2000) France; (García et al. 2005) Chile; (Gołab and Sadkowska-Todys 2005) Poland; (Greenbloom et al. 1996) Canada; (Jongwutiwes et al. 1998; Kusolsuk et al. 2010) Thailand; (Owen et al. 2005) Papua New Guinea; (Rodríguez et al. 2004) Spain *Cervidae*: (Ramasoota 1991) Thailand *Ursidae*: (Ancelle et al. 2005; Schellenberg et al. 2003) Canada; (Hall et al. 2012; Hill et al. 2005) USA; (Khamboonruang 1991) Thailand; (Yamaguchi 1991) Japan *Squamata*: (Khamboonruang 1991) Thailand
	*Trichuris* spp.	Trichuriasis	Foodborne: faecal-contaminated meat Faeco-oral: ingestion of embryonated eggs	Consumption Hunting Butchering	

### Viruses

We identified 16 zoonotic viruses potentially hosted by the traded wildlife (Table 2) and found evidence for transmission to humans in 46 references (Table 5). The *Cercopithecidae* and the *Pteropodidae* families harbour the greatest number of viruses, six and five respectively (Figure 1). Results show evidence of *Cercopithecine herpesvirus-1* (CDC 1987, 1998; Holmes et al. 1990; Weigler 1992; Huff and Barry 2003) and *Rabies virus* (Favoretto et al. 2001) infections in humans from monkeys, which cause fatal disease. The transmission of these viruses can occur from bites and scratches during hunting or via mucous membranes or damaged skin when butchering, presenting a significant risk for hunters. The genetic similarities between *Cercopithecidae* and humans risk primate-to-human transmission of viruses that may lead to emergence of novel infections within human populations, as illustrated by some simian retroviruses (Gessain et al. 2013).

The *Pteropodidae* bats potentially harbour five zoonotic viruses, and some species may be natural hosts for viral EIDs (e.g. *Nipah virus*, *Ebola virus* and novel *Reoviruses*). Surveys sampling *P. vampyrus* and *P. hypomelanus* have indicated these species are reservoir hosts for *Nipah virus* in Malaysia (Yob et al. 2001; Chua et al. 2002). Direct transmission of *Nipah virus* from *Pteropodidae* bats to people may be possible because epidemics have been reported in Bangladesh associated with human exposure to their urine and saliva (Luby et al. 2009), which should alert bat hunters and consumers to the potential transmission risks. *Lyssaviruses* should be regarded as a greater infection risk for hunters since fatal encephalitis cases have been reported in Australia from bat bites and scratches (Samaratunga et al. 1998; Hanna et al. 2000; Warrilow et al. 2002; ProMED-mail 2014a). Since *Rabies virus* and related *Lyssaviruses* are potentially hosted by five other traded taxa (*Sciuridae*, *Viverridae*, *Ursidae*, *Cercopithecidae* and *Felidae*), with several human case reports, there is a high infection risk for people hunting these animals.

### Bacteria

Nineteen bacteria were found to be potentially hosted by traded wildlife (Table 3), and evidence for zoonotic transmission to humans was identified in 61 references (Table 5). The commonly traded *Suidae* and *Cervidae* host the greatest numbers of bacterial pathogens, twelve and eleven respectively (Figure 1). Many of these bacteria can cause serious disease in humans (e.g. *Brucella,* Shiga-toxin producing *Escherichia coli* (STEC), *Leptospira* and *Mycobacterium* species) via various transmission routes, including foodborne, transcutaneous, mucosal, faeco-oral and inhalation (Table 5). Zoonotic transmission of *Brucella* infection occurs via exposure to bodily fluids or tissues and eating undercooked wild meat. Cases of brucellosis in North American (Forbes 1991; Starnes et al. 2004; Giurgiutiu et al. 2009) and Australian hunters (Robson et al. 1993; Eales et al. 2010; Irwin et al. 2010) were associated with field-dressing carcasses without personal protective equipment. Human tuberculosis may occur from cutaneous exposure to *M. bovis*, as evidenced by a deer hunter infected via a contaminated hunting knife (Wilkins et al. 2008), or the ingestion of infected meat, which occurred in Canadian deer hunters (Wilkins et al. 2003). Human cases of other bacterial zoonoses reported worldwide (listed in Table 5) highlight the significant risks posed by these wildlife taxa, which are relevant for Southeast Asia.

Several enteric bacteria are hosted across multiple traded taxa, for example *Campylobacter* (eight), *Salmonella* (ten) and *Yersinia* (five) species. Reptiles can harbour potentially human-pathogenic *Salmonella* and *Campylobacter* species, such as *S. enterica* and *C. fetus*, in their gastrointestinal tracts, which can lead to human infection via faeco-oral transmission (Friedman et al. 1998; Patrick et al. 2013). Zoonotic infection of salmonellosis occasionally occurs via transcutaneous transmission from scratches and bites. The public health risk for salmonellosis is well recognised in reptile pet owners (Corrente et al. 2006; Harris et al. 2009) and should be considered for hunters since a relatively high prevalence of *Salmonella* isolates has been detected in the faeces of free-living reptiles: 32.4% for chelonians, 40.9% for lizards (Briones et al. 2004) and 58.6% for snakes (Kuroki et al. 2013). Since human infections of *Salmonella* have occurred from eating snapping turtles in Japan (Fukushima et al. 2008), the hazard of reptile-associated foodborne salmonellosis should be considered in Southeast Asia, particularly as chelonians are widely consumed in Malaysia (Sharma and Tisen 1999). The isolation of *C. fetus* subspecies of reptile origin from an immunosuppressed patient who had eaten turtle soup (Tu et al. 2004) should raise concerns for foodborne *Campylobacter* infection from reptiles.

### Parasites

We identified 16 zoonotic parasites potentially hosted by traded wildlife (Table 4) and 40 references provided evidence for transmission to humans (Table 5). The results suggest that *Sarcocystis, Toxoplasma* and *Trichinella* species are most frequently found in wildlife. Since their lifecycles involve multiple wildlife hosts, the wild meat trade may increase the risk of zoonotic transmission, via foodborne or faeco-oral routes.

The greatest number of zoonotic parasites are found in *Cercopithecidae*, ten in total (Figure 1). Surveys of macaque populations in Asia for zoonotic gastrointestinal parasites have indicated relatively high prevalence of infection for *Balantidium coli*, *Cryptosporidia, Entamoeba histolytica* and *Giardia* (Ekanayake et al. 2007; Jha et al. 2011; Lane et al. 2011; Huffman et al. 2013), which are potentially transmitted to humans via faeco-oral and foodborne routes. One study suggested that close contact between macaques and humans at anthropogenic altered habitats may increase the risk of primate-to-human parasite transmission (Hussain et al. 2013), of relevance to the wild meat trade.

The *Suidae* and the *Cervidae* families host numerous parasites (eight and five respectively), with *Cryptosporidium*, *Giardia*, *Toxoplasma gondii* and *Trichinella* species harboured by both (Table 4). Trichinellosis poses an important disease risk because human cases related to the consumption of improperly cooked, inadequately frozen or cured wild pork and venison have been reported globally (Serrano et al. 1989; Rodríguez et al. 2004; García et al. 2005; De Bruyne et al. 2006; Meng et al. 2009), including in Southeast Asia (Ramasoota 1991; Jongwutiwes et al. 1998). In Southeast Asia, certain cultural food practices using this wild meat increase the infection risk, such as eating it raw in Thailand (Kaewpitoon et al. 2008) or undercooked in Papua New Guinea (Owen et al. 2005).

Reptiles host several parasites that pose significant foodborne infection risks to humans in Southeast Asia from the ingestion of reptile meat containing larvae or cysts, including *Gnathostoma*, *Pentastomidia*, *Sarcocystis*, *Spirometra* and *Trichinella* species (Table 5). Pentastomiasis has been reported in Malaysian aborigines associated with traditional consumption of snake meat, and some tribes have a greater risk of infection due to their preference for undercooked meat (Prathap et al. 1969; Latif et al. 2011).

### Data Deficiency

Figure 1 indicates that two wildlife taxa appear to harbour very few zoonotic pathogens, *Manidae* (zero) and *Hystricidae* (one), related to the deficiency of published studies on these taxa, which may lead to an underestimate of their zoonotic infection potential. This lack of data could be attributed to the difficulty of observing these animals in their environment due to their small size and secretive behaviour. Further research is required to determine whether *Hystricidae* species (Order: *Rodentia*) harbour more zoonoses, since surveys of other rodents have shown they can host several viruses and bacteria (Easterbrook et al. 2007; Firth et al. 2014).

## Discussion

The main objective of this review was to examine the scientific evidence for zoonotic pathogens in wildlife and human populations in order to improve understanding of the role of the wild meat trade in Malaysia for the transmission of infection to people. Whilst some recent publications have analysed the zoonotic EIDs associated with the bushmeat trade in Africa (Kilonzo et al. 2013; Kurpiers et al. 2016), to our knowledge this is the first zoonotic disease review related to the trade of wild meat in Southeast Asia. The findings identify 16 viruses, 19 bacteria and 16 parasites in the 16 traded taxonomic groups, which may pose significant public health risks to wildlife hunters and consumers at each stage of the commodity chain.

In this review, we highlight the three human risk behaviours of hunting, butchering and consumption associated with the wild meat trade, which leads to transmission of zoonoses, as supported by other literature (Karesh et al. 2012; Kilonzo et al. 2013). Hunting presents a medium risk of zoonotic infection because hunters handling animals can be bitten and scratched leading to the transcutaneous route of infection for some pathogens, particularly when they have existing skin abrasions or wounds on their hands, forearms or torso (LeBreton et al. 2006). The review provides evidence to suggest that people who process wildlife carcasses have a high risk of infection related to direct contact with blood, excretions or secretions, for example brucellosis and streptococcosis in wild pig hunters (Rosenkranz et al. 2003; Giurgiutiu et al. 2009). Some literature indicates that hunters who disregard health and safety precautions when field-dressing carcasses (Massey et al. 2011) or suffer from self-inflicted knife injuries (Eales et al. 2010) have greater risk for certain zoonotic infections. Future research should examine wildlife hunting and butchering techniques in Malaysia to evaluate the specific microbiological hazards of the wild meat trade.

We demonstrate that consuming wild meat may present a significant zoonotic risk, since the findings identify numerous pathogens potentially transmitted to humans via the foodborne route. The cultural food preferences for eating raw or undercooked wild meat in Southeast Asia (Anantaphruti et al. 2011; Latif et al. 2011) increases the transmission risk for those pathogens normally killed by cooking. Human cases of infection from the consumption of contaminated wild meat are also presented, for example, enterohaemorrhagic *E.coli* infections from wild venison (Rabatsky-Ehr et al. 2002). This information is further supported by other research that describes how microbiological contamination of meat is related to the killing process, field-dressing techniques (Paulsen 2011) and food-handling practices (Radakovic and Fletcher 2011), of relevance for the investigation of wild meat practices in Southeast Asia.

Since the availability of wild meat sold in Malaysia varies between species, there may be greater zoonotic risks to humans from the pathogens hosted by more commonly traded wildlife due to increased likelihood of exposure. Information from the review may be used to determine which pathogens from two commonly traded taxa (*Suidae* and *Cervidae*) pose significant health risks to humans, such as *Brucella* and *Mycobacterium* species, which would be beneficial for targeted disease surveillance. A recent study indicated that wild pigs and deer are commonly hunted for food by aborigines of Peninsular Malaysia (Or and Leong 2011), thus conducting epidemiological surveys on this human population at-risk of zoonotic disease would help to determine how their activities influence transmission of infection from wildlife.

The comprehensive presentation of zoonotic information in this study could enable qualitative assessment of infection risks from all the traded wildlife. However, the findings are limited by the lack of research on pathogens in the species traded, which made it necessary to utilise data from different species within the same taxonomic group. The assumption that they would be infected by similar pathogens may be reasonable for species with similar ecology, but species or geographical variation could affect infection prevalence. For example, whilst the scavenging and cannibalistic feeding behaviour of carnivorous *Ursus maritimus* has led to high prevalence of *Trichinella* infections in bears (Born and Henriksen 1990), this prevalence may be lower in omnivorous *H. malayanus* and lead to overestimation of its zoonotic potential. Additionally, the deficiency of studies for whole taxonomic groups (e.g. *Manidae* and *Hystricidae*) limits assessment of their zoonotic risk to humans. Utilising data from captive wild animal populations may overestimate the zoonotic importance of some pathogens, since environmental conditions in captivity can increase the likelihood of infection, as illustrated by circus elephants infected with *Cowpox virus* (Kurth et al. 2008; Hemmer et al. 2010) related to their exposure to hay or straw contaminated with rodent excretions (Wisser et al. 2001). To overcome these limitations, future research should survey free-ranging wild animal populations in this region for zoonotic pathogens.

The review is limited by the geographical variation in zoonotic disease reporting, with many human cases from Australia, North America and Europe. The fewer cases from Southeast Asia may reflect inadequate regional disease surveillance that contributes to underreporting (Coker et al. 2011). Hunting, butchering and consumption activities may be conducted differently in Southeast Asia compared to elsewhere due to cultural practices involving particular species [e.g. traditional uses of softshell turtles in Malaysia (Sharma 1999)] and so the regional deficiency of research may underestimate the zoonotic risks posed by these species. Therefore, it is also necessary to increase zoonotic disease monitoring and surveillance of at-risk human populations in Southeast Asia.

We highlight a knowledge gap in understanding the zoonotic implications of the wild meat trade in Southeast Asia and suggest that this is related to numerous factors. Primarily, there is insufficient zoonotic disease surveillance of wild animal and human populations in this region due to limited resources, weak reporting systems, lack of government policies and underdeveloped veterinary services (Coker et al. 2011). Few surveys of wildlife populations in Southeast Asia for zoonotic pathogens have been conducted (Jones-Engel et al. 2007; Jittapalapong et al. 2011; Thayaparan et al. 2013), and even fewer studies have sampled wild meat for zoonoses of relevance to wildlife consumers (Fazly et al. 2013). Whilst livestock carcasses undergo routine meat inspections to prevent foodborne zoonoses, this does not occur for wildlife carcasses intended for human consumption (Fazly et al. 2013). Since hunting to supply the wild meat trade may often contravene national legislation protecting species, if hunters or consumers contract a zoonotic infection from their illegal activities they may not report it to medical services, which likely leads to an underreporting of cases. This is further exacerbated by the limited availability of healthcare services in many Southeast Asia countries (Coker et al. 2011), particularly for people in rural areas where wildlife hunting and consumption frequently occurs.

Information from the review would be useful in guiding cross-disciplinary studies to investigate the dynamics of zoonotic disease spillover and emergence (Daszak et al. 2007) associated with wild meat trade in Southeast Asia. The findings suggest concentrating EID research on traded species that host zoonotic pathogens of greatest risk to humans, particularly those harbouring RNA viruses (e.g. Old World monkeys, flying foxes and civets) since these viruses can undergo genetic mutations and rapidly adapt to changing environmental conditions (Ludwig et al. 2003). This is relevant for Southeast Asia where the combination of anthropogenic activities, including wildlife hunting, deforestation and urbanisation, leads to greater human encroachment into natural habitats, thus increasing the risk of cross-species infection (Weiss and McMichael 2004), which threatens human, animal and ecosystem health (Rabinowitz and Conti 2013). Consequently, this study is useful for health professionals, wildlife researchers and conservationists who work at locations where significant human–wildlife interactions occur and want to understand the implications of the wild meat trade on zoonotic disease transmission.

The findings also highlight the importance of endemic and neglected zoonoses being transmitted to humans from traded wildlife, such as sarcocystosis (Tappe et al. 2013). These zoonotic infections would benefit from increased targeted disease surveillance and application of One Health approaches to integrate public health, veterinary science, epidemiology, ecology and sociology (Karesh et al. 2012) in Southeast Asia.

This study could be used in the development of public health strategies in Southeast Asia to dissuade people from harvesting wildlife for food by educating them about the numerous health risks highlighted and encourage their consumption of alternative foods. Such initiatives could have additional benefits for the conservation of threatened species, by helping to reduce the illegal international trade of reptiles and mammals for their meat that occurs in this region (Nijman 2010).

Overall, information from the review indicates the deficits in epidemiological knowledge related to Southeast Asia that suggests future research should include surveys of traded wildlife and at-risk human populations for zoonotic pathogens, with increased investigation of disease outbreaks. Since numerous zoonoses may be transmitted via foodborne routes, it would be beneficial to conduct microbial food safety risk assessments in this region that follow the Codex Alimentarius Commission framework (CAC 1999), which evaluate the consumer risk for specific pathogens from wild meat. These assessments would require microbial analysis of wildlife carcasses and investigations of the wild meat production chain to examine environmental conditions and hygienic practices (Gill 2007; Paulsen 2011) for producing a final risk estimate (CAC 1999). Some interview-based surveys of wildlife hunters and consumers in Southeast Asia have investigated the social and cultural factors driving wild meat consumption (Drury 2011; Scheffers et al. 2012), and this methodology could be applied in Malaysia to examine how people’s behaviour influences their risk of zoonoses. Such information may contribute to public health initiatives that focus on the health and safety of people involved in the wild meat trade.

In conclusion, the great diversity of potentially zoonotic pathogens in wildlife hunted for food in Malaysia is highlighted in this review, with some taxa hosting numerous infectious agents, including *Cercopithecidae*, *Suidae* and *Cervidae*. The subsequent examination of infection risks and transmission routes to humans associated with this trade illustrates the variation in zoonotic risk posed by different taxa and identifies gaps in epidemiological knowledge for some species. The findings assist in evaluating the level of infection risk to humans related to the different stages of the wild meat chain, associated with the wildlife host, pathogen transmission route(s) and behaviour of people involved. This comprehensive study could help guide future zoonotic research and disease surveillance of wild animal and at-risk human populations in Southeast Asia, which is beneficial for One Health projects located here. Our intention is to increase awareness about the possible human health risks from this trade, which are relevant for public health and conservation strategies in the region.

## Electronic supplementary material

Below is the link to the electronic supplementary material.

Supplementary material 1 (DOCX 304 kb)

## References

[CR1] Abernethy K, Coad L, Taylor G, Lee M, Maisels F (2013). Extent and ecological consequences of hunting in Central African rainforests in the twenty-first century. Philosophical Transactions of the Royal Society B: Biological Sciences.

[CR2] Addidle M, Grimwade K, Tie S, Rahman H, Sorenson R (2009). “Pigs might fly”—a case of Erysipelothrix endocarditis. Journal of the New Zealand Medical Association.

[CR3] Akahane H, Sano M, Kobayashi M (1998). Three cases of human gnathostomiasis caused by Gnathostoma hispidum, with particular reference to the identification of parasitic larvae. Southeast Asian Journal of Tropical Medicine and Public Health.

[CR4] Alvarado-Esquivel C, Cruz-Magallanes H, Esquivel-Cruz R, Estrada-Martínez S, Rivas-González M, Liesenfeld O, Martínez-García S, Ramírez E, Torres-Castorena A, Castañeda A (2008). Seroepidemiology of Toxoplasma gondii infection in human adults from three rural communities in Durango State, Mexico. Journal of Parasitology.

[CR5] Anantaphruti MT, Nawa Y, Vanvanitchai Y (2011). Human sparganosis in Thailand: an overview. Acta tropica.

[CR6] Ancelle T, De Bruyne A, Poisson D, Dupouy-Camet J (2005). Outbreak of trichinellosis due to consumption of bear meat from Canada, France, September 2005. Euro Surveillance.

[CR7] Anderson DC, Geistfeld JG, Maetz HM, Patton CM, Kaufmann AF (1978). Leptospirosis in zoo workers associated with bears. The American journal of tropical medicine and hygiene.

[CR8] Atlas R, Rubin C, Maloy S, Daszak P, Colwell R, Hyde B (2010). One health–attaining optimal health for people, animals, and the environment. Microbe.

[CR2000] Ayouba A, Akoua-Koffi C, Calvignac-Spencer S, Esteban A, Locatelli S, Li H, Li Y, Hahn BH, Delaporte E, Leendertz FH, Peeters M (2013). Evidence for continuing cross-species transmission of SIVsmm to humans: characterization of a new HIV-2 lineage in rural Côte d’Ivoire. AIDS.

[CR9] Baker M, Lopez L, Cannon M, De Lisle G, Collins D (2006). Continuing *Mycobacterium bovis* transmission from animals to humans in New Zealand. Epidemiology and Infection.

[CR10] Bell D, Roberton S, Hunter PR (2004). Animal origins of SARS coronavirus: possible links with the international trade in small carnivores. Philosophical Transactions of the Royal Society of London. Series B: Biological Sciences.

[CR11] Bennett E, Nyaoi A, Sompud J, York New (2000). Saving Borneo’s bacon: the sustainability of hunting in Sarawak and Sabah. *Hunting for sustainability in tropical forests,* Robinson J and Bennett E.

[CR12] Bennett EL (2002). Is there a link between wild meat and food security?. Conservation Biology.

[CR13] Bhatt BD, Zuckerman MJ, Foland JA, Polly SM, Marwah RK (1989). Disseminated Salmonella arizona infection associated with rattlesnake meat ingestion. The American journal of gastroenterology.

[CR14] Born E, Henriksen S (1990). Prevalence of Trichinella sp. in polar bears (Ursus maritimus) from northeastern Greenland. Polar Research.

[CR15] Bow MR, Brown JH (1946). Tularemia. A report on 40 cases in Alberta, Canada, 1931–1944. American Journal of Public Health and the Nations Health.

[CR16] Briones V, Téllez S, Goyache J, Ballesteros C, del Pilar Lanzarot M, Domínguez L, Fernández-Garayzábal JF (2004). Salmonella diversity associated with wild reptiles and amphibians in Spain. Environmental microbiology.

[CR17] Brody JA, Huntley B, Overfield TM, Maynard J (1966). Studies of human brucellosis in Alaska. The Journal of infectious diseases.

[CR18] Brooks JI, Rud EW, Pilon RG, Smith JM, Switzer WM, Sandstrom PA (2002). Cross-species retroviral transmission from macaques to human beings. The Lancet.

[CR19] Brown R (2005) Leptospirosis in deer slaughter premises. In *Proceedings of the Deer Branch of the New Zealand Veterinary Association*. New Zealand, p 120

[CR20] CAC (1999) Principles and guidelines for the conduct of a microbiological risk assessment In *Codex Alimentarius Food Hygeine Basic Texts,* Rome, Italy: Codex Alimentarius Commission, Joint FAO/WHO Food Standards Programme, pp 53–62

[CR21] Caillabet OS, Krishnasamy K, Khan S (Unpublished). In the soup: a survey of the availability of wild meat in Malaysia. TRAFFIC Southeast Asia, Petaling Jaya, Selangor, Malaysia

[CR22] Capitini CM, Herrero IA, Patel R, Ishitani MB, Boyce TG (2002). Wound Infection with Neisseria weaveri and a Novel Subspecies of *Pasteurella multocida* in a Child Who Sustained a Tiger Bite. Clinical Infectious Diseases.

[CR23] Carme B, Demar M, Ajzenberg D, Dardé ML (2009). Severe acquired toxoplasmosis caused by wild cycle of *Toxoplasma gondii*, French Guiana. Emerging infectious diseases.

[CR24] Carrington M, Choe U, Ubillos S, Stanek D, Campbell M, Wansbrough L, Lee P, Churchwell G, Rosas K, Zaki S (2012). Fatal case of brucellosis misdiagnosed in early stages of Brucella suis infection in a 46-year-old patient with Marfan syndrome. Journal of clinical microbiology.

[CR25] CDC (1987). Epidemiologic notes and reports B-virus infection in humans—Pensacola, Florida. Morbility and Mortality Weekly Report (Centers for Disease Control and Prevention).

[CR26] CDC (1998) Fatal Cercopithecine herpesvirus 1 (B virus) infection following a mucocutaneous exposure and interim recommendations for worker protection. *Morbility and Mortality Weekly Report (Centers for Disease Control and Prevention)* 47:1073–1076 & 1083 9879633

[CR27] Chaber AL, Allebone-Webb S, Lignereux Y, Cunningham AA, Marcus Rowcliffe J (2010). The scale of illegal meat importation from Africa to Europe via Paris. Conservation Letters.

[CR28] Chan J, Baxter C, Wenman WM (1989). Brucellosis in an Inuit child, probably related to caribou meat consumption. Scandinavian journal of infectious diseases.

[CR29] Chase D, Handsfield H, Allard J, Taylor J (1980). Tularemia acquired from a bear: Washington. Morbidity and Mortality Weekly Report.

[CR30] Choi WYNHW, Kwak NH, Huh W, Kim YR, Kang MW, Cho SY, Dubey JP (1997). Foodborne outbreaks of human toxoplasmosis. Journal of Infectious Diseases.

[CR31] Chua KB, Crameri G, Hyatt A, Yu M, Tompang MR, Rosli J, McEachern J, Crameri S, Kumarasamy V, Eaton BT, Wang L-F (2007). A previously unknown reovirus of bat origin is associated with an acute respiratory disease in humans. Proceedings of the National Academy of Sciences.

[CR32] Chua KB, Lek Koh C, Hooi PS, Wee KF, Khong JH, Chua BH, Chan YP, Lim ME, Lam SK (2002). Isolation of Nipah virus from Malaysian Island flying-foxes. Microbes and Infection.

[CR33] Coker RJ, Hunter BM, Rudge JW, Liverani M, Hanvoravongchai P (2011). Emerging infectious diseases in southeast Asia: regional challenges to control. The Lancet.

[CR34] Corrente M, Totaro M, Martella V, Campolo M, Lorusso A, Ricci M, Buonavoglia C (2006). Reptile-associated salmonellosis in man, Italy. Emerging infectious diseases.

[CR35] Cui J, Wang Z, Xu B (2011). The epidemiology of human trichinellosis in China during 2004–2009. Acta tropica.

[CR36] Dalsjö A, Nilsson AC, Ramussen M (2014). Complicated infection caused by Streptococcus suis serotype 14 transmitted from a wild boar. Journal of Medical Microbiology Case Reports.

[CR37] Daszak P, Epstein J, Kilpatrick A, Aguirre A, Karesh W, Cunningham A, Childs JE, Mackenzie JS, Richt JA (2007). Collaborative research approaches to the role of wildlife in zoonotic disease emergence. Wildlife and Emerging Zoonotic Diseases: The Biology, Circumstances and Consequences of Cross-Species Transmission.

[CR38] Davies TJ, Pedersen AB (2008). Phylogeny and geography predict pathogen community similarity in wild primates and humans. Proceedings of the Royal Society B: Biological Sciences.

[CR39] De Bruyne A, Ancelle T, Vallee I, Boireau P, Dupouy-Camet J (2006). Human trichinellosis acquired from wild boar meat: a continuing parasitic risk in France. Euro Surveillance.

[CR40] Deutz A, Fuchs K, Schuller W, Nowotny N, Auer H, Aspöck H, Stünzner D, Kerbl U, Klement C, Köfer J (2002). Seroepidemiological studies of zoonotic infections in hunters in southeastern Austria–prevalences, risk factors, and preventive methods. Berliner und Munchener tierarztliche Wochenschrift.

[CR41] Diesch S, Crawford R, McCulloch W, Top F (1967). Human leptospirosis acquired from squirrels. New England Journal of Medicine.

[CR42] Drury R (2011). Hungry for success: urban consumer demand for wild animal products in Vietnam. Conservation and Society.

[CR43] Durazo A, Lessenger JE, Lessenger JE (2006). Mammal Bites. Agricultural Medicine: A Practical Guide, JE.

[CR44] Eales KM, Norton RE, Ketheesan N (2010). Brucellosis in northern Australia. The American journal of tropical medicine and hygiene.

[CR45] Easterbrook JD, Kaplan JB, Vanasco NB, Reeves WK, Purcell RH, Kosoy MY, Glass GE, Watson J, Klein SL (2007). A survey of zoonotic pathogens carried by Norway rats in Baltimore, Maryland, USA. Epidemiology and infection.

[CR46] Ekanayake DK, Welch DM, Kieft R, Hajduk S, Dittus WP (2007). Transmission dynamics of Cryptosporidium infection in a natural population of non-human primates at Polonnaruwa, Sri Lanka. The American journal of tropical medicine and hygiene.

[CR47] Engels EA, Switzer WM, Heneine W, Viscidi RP (2004). Serologic evidence for exposure to simian virus 40 in North American zoo workers. Journal of Infectious Diseases.

[CR48] Epstein JH, Prakash V, Smith CS, Daszak P, McLaughlin AB, Meehan G, Field HE, Cunningham AA (2008). Henipavirus infection in fruit bats (Pteropus giganteus), India. Emerging infectious diseases.

[CR49] Esmaeili S, Gooya MM, Shirzadi MR, Esfandiari B, Amiri FB, Behzadi MY, Banafshi O, Mostafavi E (2014). Seroepidemiological survey of tularemia among different groups in western Iran. International Journal of Infectious Diseases.

[CR50] Fan PC (1988). Taiwan Taenia and Taeniasis. Parasitology Today.

[CR51] Fan PC, Chung WC, Soh CT, Kosman ML (1992). Eating habits of East Asian people and transmission of taeniasis. Acta tropica.

[CR52] Fanning A, Edwards S (1991). Mycobacterium bovis infection in human beings in contact with elk (Cervus elaphus) in Alberta, Canada. The Lancet.

[CR53] Fasanella A, Palazzo L, Petrella A, Quaranta V, Romanelli B, Garofolo G (2007). Anthrax in red deer (Cervus elaphus), Italy. Emerging infectious diseases.

[CR54] Favoretto SR, de Mattos CC, Morais NB, Araújo FA, de Mattos CA (2001). Rabies in marmosets (Callithrix jacchus), Ceará, Brazil. Emerging infectious diseases.

[CR55] Fazly Z, Nurulaini R, Shafarin M, Fariza N, Zawida Z, Muhamad H, Adnan M, Premaalatha B, Erwanas A, Zaini C (2013). Zoonotic parasites from exotic meat in Malaysia. Tropical biomedicine.

[CR56] Firth C, Bhat M, Firth MA, Williams SH, Frye MJ, Simmonds P, Conte JM, Ng J, Garcia J, Bhuva NP, Lee B, Che X, Quan P-L, Lipkin WI (2014) Detection of zoonotic pathogens and characterization of novel viruses carried by commensal rattus norvegicus in New York City. *mBio* 5:e01933–01914 10.1128/mBio.01933-14 PMC4205793 25316698

[CR57] Forbes LB (1991). Isolates of Brucella suis biovar 4 from animals and humans in Canada, 1982–1990. The Canadian Veterinary Journal.

[CR58] Friedman CR, Torigian C, Shillam PJ, Hoffman RE, Heltze D, Beebe JL, Malcolm G, DeWitt WE, Hutwagner L, Griffin PM (1998). An outbreak of salmonellosis among children attending a reptile exhibit at a zoo. The Journal of pediatrics.

[CR59] Fukushima H, Okuno J, Fujiwara Y, Hosoda T, Kurazono T, Ohtsuka K, Yanagawa K, Yamaguchi M (2008). An outbreak of Salmonella food poisoning at a snapping turtle restaurant. Journal of the Japanese Association for Infectious Diseases.

[CR60] García E, Mora L, Torres P, Jercic MI, Mercado R (2005). First record of human trichinosis in Chile associated with consumption of wild boar (Sus scrofa). Memorias do Instituto Oswaldo Cruz.

[CR61] Garin-Bastuji B, Vaillant V, Albert D, Tourrand B, Danjean M, Lagier A, Rispal P, Benquet B, Maurin M, De Valk H (2006). Is brucellosis due the biovar 2 of Brucella suis an emerging zoonosis in France? Two case reports in wild boar and hare hunters. *Proceedings of the International Society of Chemotherapy Disease Management Meeting, 1st International Meeting on Treatment of Human Brucellosis* Loannina.

[CR62] Gessain A, Rua R, Betsem E, Turpin J, Mahieux R (2013). HTLV-3/4 and simian foamy retroviruses in humans: Discovery, epidemiology, cross-species transmission and molecular virology. Virology.

[CR63] Gill C (2007). Microbiological conditions of meats from large game animals and birds. Meat science.

[CR64] Giurgiutiu D, Banis C, Hunt E, Mincer J, Nicolardi C, Weltman A, Stanek D, Matthews S, Siegenthaler C, Blackmore C (2009). Brucella suis infection associated with feral swine hunting-Three States, 2007–2008. Morbidity and Mortality Weekly Report.

[CR65] Gołab E, Sadkowska-Todys M (2005). Epidemiology of human trichinellosis in Poland–currently and in the past. Wiadomosci parazytologiczne.

[CR66] Greenbloom SL, Martin-Smith P, Isaacs S, Marshall B, Kittle DC, Kain KC, Keystone JS (1996). Outbreak of trichinosis in Ontario secondary to the ingestion of wild boar meat. Canadian journal of public health.

[CR67] Halaby T, Hoitsma E, Hupperts R, Spanjaard L, Luirink M, Jacobs J (2000). Streptococcus suis meningitis, a poacher’s risk. European Journal of Clinical Microbiology and Infectious Diseases.

[CR68] Hall RL, Lindsay A, Hammond C, Montgomery SP, Wilkins PP, da Silva AJ, McAuliffe I, de Almeida M, Bishop H, Mathison B (2012). Outbreak of human trichinellosis in Northern California caused by Trichinella murrelli. The American journal of tropical medicine and hygiene.

[CR69] Hanna JN, Carney IK, Smith GA, Tannenberg A, Deverill JE, Botha JA, Serafin IL, Harrower BJ, Fitzpatrick PF, Searle JW (2000). Australian bat lyssavirus infection: a second human case, with a long incubation period. The Medical Journal of Australia.

[CR70] Harris JR, Bergmire-Sweat D, Schlegel JH, Winpisinger KA, Klos RF, Perry C, Tauxe RV, Sotir MJ (2009). Multistate outbreak of Salmonella infections associated with small turtle exposure, 2007–2008. Pediatrics.

[CR71] Hemmer CJ, Littmann M, Löbermann M, Meyer H, Petschaelis A, Reisinger EC (2010). Human cowpox virus infection acquired from a circus elephant in Germany. *International journal of infectious diseases* 14. Supplement.

[CR72] Hill D, Gamble H, Zarlenga D, Coss C, Finnigan J (2005). Trichinella nativa in a black bear from Plymouth, New Hampshire. Veterinary parasitology.

[CR73] Holmes GP, Hilliard JK, Klontz KC, Rupert AH, Schindler CM, Parrish E, Griffin DG, Ward GS, Bernstein ND, Bean TW (1990). B virus (Herpesvirus simiae) infection in humans: epidemiologic investigation of a cluster. Annals of internal medicine.

[CR74] Huang F, Wang H, Jing S, Zeng W (2012). Simian foamy virus prevalence in Macaca mulatta and zookeepers. AIDS research and human retroviruses.

[CR75] Huff JL, Barry PA (2003). B-virus (Cercopithecine herpesvirus 1) infection in humans and macaques: potential for zoonotic disease. Emerging infectious diseases.

[CR76] Huffman M, Nahallage C, Hasegawa H, Ekanayake S, De Silva L, Athauda I (2013). Preliminary survey of the distribution of four potentially zoonotic parasite species among primates in Sri Lanka. Journal of the National Science Foundation of Sri Lanka.

[CR77] Hussain S, Ram MS, Kumar A, Shivaji S, Umapathy G (2013). Human presence increases parasitic load in endangered lion-tailed macaques (Macaca silenus) in its fragmented rainforest habitats in southern India. PLoS ONE.

[CR78] Ichhpujani R, Rajagopal V, Bhattacharya D, Rana U, Mittal V, Rai A, Ravishankar A, Pasha S, Sokhey J, Biswas S (2004). An outbreak of human anthrax in Mysore (India). The Journal of communicable diseases.

[CR79] Ingram DJ, Coad L, Collen B, Kümpel NF, Breuer T, Fa JE, Gill DJ, Maisels F, Schleicher J, Stokes EJ (2015). Indicators for wild animal offtake: methods and case study for African mammals and birds. Ecology & Society.

[CR80] Irwin MJ, Massey PD, Walker B, Durrheim DN (2010). Feral pig hunting: a risk factor for human brucellosis in north-west NSW?. New South Wales public health bulletin.

[CR81] Isotalo P, Edgar D, Toye B (2000). Polymicrobial tenosynovitis with Pasteurella multocida and other gram negative bacilli after a Siberian tiger bite. Journal of clinical pathology.

[CR82] Jha A, Chalise MK, Shrestha RM, Karki K (2011). Intestinal Parasitic Investigation in Temple Rhesus Monkeys of Kathmandu. The Initiation.

[CR83] Jittapalapong S, Sarataphan N, Maruyama S, Hugot J-P, Morand S, Herbreteau V (2011). Toxoplasmosis in rodents: ecological survey and first evidences in Thailand. Vector-Borne and Zoonotic Diseases.

[CR84] Jones-Engel L, Engel GA, Schillaci MA, Rompis A, Putra A, Suaryana KG, Fuentes A, Beer B, Hicks S, White R (2005). Primate-to-human retroviral transmission in Asia. Emerging infectious diseases.

[CR85] Jones-Engel L, May CC, Engel GA, Steinkraus KA, Schillaci MA, Fuentes A, Rompis A, Chalise MK, Aggimarangsee N, Feeroz MM (2008). Diverse contexts of zoonotic transmission of simian foamy viruses in Asia. Emerging infectious diseases.

[CR86] Jones-Engel L, Steinkraus KA, Murray SM, Engel GA, Grant R, Aggimarangsee N, Lee BP-H, May C, Schillaci MA, Somgird C (2007). Sensitive assays for simian foamy viruses reveal a high prevalence of infection in commensal, free-ranging Asian monkeys. Journal of virology.

[CR87] Jongwutiwes S, Chantachum N, Kraivichian P, Siriyasatien P, Putaporntip C, Tamburrini A, La Rosa G, Sreesunpasirikul C, Yingyourd P, Pozio E (1998). First outbreak of human trichinellosis caused by Trichinella pseudospiralis. Clinical Infectious Diseases.

[CR88] Kaewpitoon N, Kaewpitoon SJ, Pengsaa P (2008). Food-borne parasitic zoonosis: distribution of trichinosis in Thailand. World journal of gastroenterology.

[CR89] Kamins AO, Rowcliffe JM, Ntiamoa-Baidu Y, Cunningham AA, Wood JLN, Restif O (2015). Characteristics and Risk Perceptions of Ghanaians Potentially Exposed to Bat-Borne Zoonoses through Bushmeat. EcoHealth.

[CR90] Karesh WB, Cook RA, Bennett EL, Newcomb J (2005). Wildlife trade and global disease emergence. Emerging infectious diseases.

[CR91] Karesh WB, Cook RA, Gilbert M, Newcomb J (2007). Implications of wildlife trade on the movement of avian influenza and other infectious diseases. Journal of Wildlife Diseases.

[CR92] Karesh WB, Dobson A, Lloyd-Smith JO, Lubroth J, Dixon MA, Bennett M, Aldrich S, Harrington T, Formenty P, Loh EH (2012). Ecology of zoonoses: natural and unnatural histories. The Lancet.

[CR93] Keene WE, Sazie E, Kok J, Rice DH, Hancock DD, Balan VK, Zhao T, Doyle MP (1997). An outbreak of Escherichia coli 0157: H7 infections traced to jerky made from deer meat. The Journal of the American Medical Association.

[CR94] Kelly J, Hopkin R, Rimsza ME (1995). Rattlesnake meat ingestion and Salmonella arizona infection in children: case report and review of the literature. The Pediatric infectious disease journal.

[CR95] Kennedy FM, Astbury J, Needham J, Cheasty T (1993). Shigellosis due to occupational contact with non-human primates. Epidemiology and infection.

[CR96] Khamboonruang C (1991). The present status of trichinellosis in Thailand. Southeast Asian Journal of Tropical Medicine Public Health.

[CR97] Kilonzo C, Stopka TJ, Chomel B, Singh SK (2013). Illegal animal and (bush) meat trade associated risk of spread of viral infections. Viral infections and global change.

[CR98] Kuhl JT, Huerter CJ, Hashish H (2003). A case of human orf contracted from a deer. Cutis.

[CR99] Kuhn K, Torpdahl M, Frank C, Sigsgaard K, Ethelberg S (2011). An outbreak of Salmonella Typhimurium traced back to salami, Denmark, April to June 2010. Euro Surveillance.

[CR100] Kumari PL, Mohanan KR, Kailas L, Chacko KP (2014). A Case of Rabies after Squirrel Bite. The Indian Journal of Pediatrics.

[CR101] Kuroki T, Ishihara T, Furukawa I, Okatani AT, Kato Y (2013). Prevalence of Salmonella in Wild Snakes in Japan. Japanese journal of infectious diseases.

[CR102] Kurpiers LA, Schulte-Herbrüggen B, Ejotre I, Reeder DM, Angelici MF (2016). Bushmeat and Emerging Infectious Diseases: Lessons from Africa. Problematic Wildlife: A Cross-Disciplinary Approach.

[CR103] Kurth A, Wibbelt G, Gerber H-P, Petschaelis A, Pauli G, Nitsche A (2008). Rat-to-elephant-to-human transmission of cowpox virus. Emerging infectious diseases.

[CR104] Kusolsuk T, Kamonrattanakun S, Wesanonthawech A, Dekumyoy P, Thaenkham U, Yoonuan T, Nuamtanong S, Sa-nguankiat S, Pubampen S, Maipanich W (2010). The second outbreak of trichinellosis caused by Trichinella papuae in Thailand. Transactions of the Royal Society of Tropical Medicine and Hygiene.

[CR105] Lane KE, Holley C, Hollocher H, Fuentes A (2011). The anthropogenic environment lessens the intensity and prevalence of gastrointestinal parasites in Balinese long-tailed macaques (Macaca fascicularis). Primates.

[CR106] Latif B, Omar E, Heo CC, Othman N, Tappe D (2011). Human Pentastomiasis Caused by *Armillifer moniliformis* in Malaysian Borneo. The American journal of tropical medicine and hygiene.

[CR107] Lau YL, Chang PY, Tan CT, Fong MY, Mahmud R, Wong KT (2014). Sarcocystis nesbitti infection in human skeletal muscle: possible transmission from snakes. The American journal of tropical medicine and hygiene.

[CR108] LeBreton M, Prosser A, Tamoufe U, Sateren W, Mpoudi-Ngole E, Diffo J, Burke D, Wolfe N (2006). Patterns of bushmeat hunting and perceptions of disease risk among central African communities. Animal Conservation.

[CR109] Lerche NW, Switzer WM, Yee JL, Shanmugam V, Rosenthal AN, Chapman LE, Folks TM, Heneine W (2001). Evidence of infection with simian type D retrovirus in persons occupationally exposed to nonhuman primates. Journal of virology.

[CR110] Leroy EM, Rouquet P, Formenty P, Souquiere S, Kilbourne A, Froment J-M, Bermejo M, Smit S, Karesh W, Swanepoel R (2004). Multiple Ebola virus transmission events and rapid decline of central African wildlife. Science.

[CR111] Li M, Song Y, Li B, Wang Z, Yang R, Jiang L, Yang R (2005). Asymptomatic yersinia pestis infection, China. Emerging infectious diseases.

[CR112] Li T, Chijiwa K, Sera N, Ishibashi T, Etoh Y, Shinohara Y, Kurata Y, Ishida M, Sakamoto S, Takeda N (2005). Hepatitis E virus transmission from wild boar meat. Emerging infectious diseases.

[CR113] Li W, Shi Z, Yu M, Ren W, Smith C, Epstein JH, Wang H, Crameri G, Hu Z, Zhang H (2005). Bats are natural reservoirs of SARS-like coronaviruses. Science.

[CR114] Liss GM, Wong L, Kittle D, Simor A, Naus M, Martiquet P, Misener C (1993). Occupational exposure to Mycobacterium bovis infection in deer and elk in Ontario. Canadian journal of public health.

[CR115] Luby SP, Hossain MJ, Gurley ES, Ahmed B-N, Banu S, Khan SU, Homaira N, Rota PA, Rollin PE, Comer JA (2009). Recurrent zoonotic transmission of Nipah virus into humans, Bangladesh, 2001–2007. Emerging infectious diseases.

[CR116] Luby SP, Rahman M, Hossain MJ, Blum LS, Husain MM, Gurley E, Khan R, Ahmed B-N, Rahman S, Nahar N (2006). Foodborne transmission of Nipah virus, Bangladesh. Emerging infectious diseases.

[CR117] Ludwig B, Kraus FB, Allwinn R, Doerr HW, Preiser W (2003). Viral Zoonoses – A Threat under Control?. Intervirology.

[CR118] Luskin M, Christina E, Kelley L, Potts M (2014). Modern Hunting Practices and Wild Meat Trade in the Oil Palm Plantation-Dominated Landscapes of Sumatra, Indonesia. Human Ecology.

[CR119] Madar CS, Cardile AP, Cunningham S, Magpantay G, Finger D (2012). A case of Salmonella gastroenteritis following ingestion of raw venison sashimi. Hawai’i Journal Of Medicine & Public Health: A Journal Of Asia Pacific Medicine & Public Health.

[CR120] Magee JS, Steele RW, Kelly NR, Jacobs RF (1989). Tularemia transmitted by a squirrel bite. The Pediatric infectious disease journal.

[CR121] Marano N, Arguin PM, Pappaioanou M (2007). Impact of globalization and animal trade on infectious disease ecology. Emerging infectious diseases.

[CR122] Massey P, Polkinghorne B, Durrheim D, Lower T, Speare R (2011). Blood, guts and knife cuts: reducing the risk of swine brucellosis in feral pig hunters in north-west New South Wales, Australia. Rural and remote health.

[CR123] Masuda J-I, Yano K, Tamada Y, Takii Y, Ito M, Omagari K, Kohno S (2005). Acute hepatitis E of a man who consumed wild boar meat prior to the onset of illness in Nagasaki, Japan. Hepatology Research.

[CR124] Masuzawa T, Okamoto Y, Une Y, Takeuchi T, Tsukagoshi K, Koizumi N, Kawabata H, Ohta S, Yoshikawa Y (2006). Leptospirosis in squirrels imported from United States to Japan. Emerging infectious diseases.

[CR125] Matsuda H, Okada K, Takahashi K, Mishiro S (2003). Severe hepatitis E virus infection after ingestion of uncooked liver from a wild boar. Journal of Infectious Diseases.

[CR126] Maxwell S, Fuller R, Brooks T, Watson J (2016). Biodiversity: The ravages of guns, nets and bulldozers. Nature.

[CR127] McDonald JC, Gyorkos TW, Alberton B, MacLean JD, Richer G, Juranek D (1990). An outbreak of toxoplasmosis in pregnant women in northern Quebec. Journal of Infectious Diseases.

[CR128] Meng X, Lindsay D, Sriranganathan N (2009). Wild boars as sources for infectious diseases in livestock and humans. Philosophical Transactions of the Royal Society: Biological Sciences.

[CR129] Meyer M (1966). Identification and virulence studies of Brucella strains isolated from Eskimos and reindeer in Alaska, Canada, and Russia. American journal of veterinary research.

[CR130] Michalak K, Austin C, Diesel S, Bacon M, Zimmerman P, Maslow JN (1998). Mycobacterium tuberculosis infection as a zoonotic disease: transmission between humans and elephants. Emerging infectious diseases.

[CR131] Milner-Gulland EJ, Bennett EL (2003). Wild meat: the bigger picture. Trends in Ecology & Evolution.

[CR132] Min D-Y (1990). Cestode infections in Korea. Korean Journal of Parasitology.

[CR133] Miranda M, Ksiazek T, Retuya T, Khan AS, Sanchez A, Fulhorst CF, Rollin PE, Calaor A, Manalo D, Roces M (1999). Epidemiology of Ebola (subtype Reston) virus in the Philippines, 1996. Journal of Infectious Diseases.

[CR134] Miyazaki S, Ishii T, Matoba S, Awatani T, Toda I (2001). A case of cat-scratch disease from a masked palm civet in Japan. Monthly Community Medicine.

[CR135] Morikawa S, Saijo M, Kurane I (2007). Current knowledge on lower virulence of Reston Ebola virus *Comparative Immunology*. Microbiology and Infectious Diseases.

[CR136] Murphree R, Warkentin JV, Dunn JR, Schaffner W, Jones TF (2011). Elephant-to-human transmission of tuberculosis, 2009. Emerging infectious diseases.

[CR137] Mutalib A, Keirs R, Austin F (1995) Erysipelas in quail and suspected erysipeloid in processing plant employees. *Avian Diseases* 39:191–193 7794182

[CR138] Nagano H, Hirochi T, Fujita K, Wakamori Y, Takeshi K, Yano S (2004). Phenotypic and genotypic characterization of β-d-glucuronidase-positive Shiga toxin-producing Escherichia coli O157: H7 isolates from deer. Journal of Medical Microbiology.

[CR139] Nagel P, Serritella A, Layden TJ (1982). Edwardsiella tarda Gastroenteritis Associated with a Pet Turtle. Gastroenterology.

[CR140] Nation PN, Fanning EA, Hopf HB, Church TL (1999). Observations on animal and human health during the outbreak of Mycobacterium bovis in game farm wapiti in Alberta. The Canadian Veterinary Journal.

[CR141] Nijman V (2010). An overview of international wildlife trade from Southeast Asia. Biodiversity and conservation.

[CR142] Or OC, Leong TF (2011). Orang Asli and Wildlife Conservation in the Belum-Temengor Forest Complex. Malaysia. TRAFFIC Bulletin.

[CR143] Owen IL, Morales MAG, Pezzotti P, Pozio E (2005). Trichinella infection in a hunting population of Papua New Guinea suggests an ancient relationship between Trichinella and human beings. Transactions of the Royal Society of Tropical Medicine and Hygiene.

[CR144] Pandit S (1950). Two instances of proved rabies in the tiger. Indian medical gazette.

[CR145] Park HY, Lee SU, Kim SH, Lee PC, Huh S, Yang YS, Kong Y (2001). Epidemiological significance of sero-positive inhabitants against sparganum in Kangwon-do, Korea. Yonsei medical journal.

[CR146] Patrick ME, Gilbert MJ, Blaser MJ, Tauxe RV, Wagenaar JA, Fitzgerald C (2013). Human infections with new subspecies of Campylobacter fetus. Emerging infectious diseases.

[CR147] Paulsen P, Bauer A, Vodnansky M, Winkelmayer R, Smulders FJM (2011). Hygiene and microbiology of meat from wild game: an Austrian view. *Game meat hygiene in focus: Microbiology, epidemiology, risk analysis and quality assurance,* Paulsen P.

[CR148] Peres CA (2000). Effects of subsistence hunting on vertebrate community structure in Amazonian forests. Conservation Biology.

[CR3000] Pernet O, Schneider BS, Beaty SM, LeBreton M, Yun TE, Park A, Zachariah TT, Bowden TA, Hitchens P, Ramirez CM, Daszak P, Mazet J, Freiberg AN, Wolfe ND, Lee B (2014). Evidence for henipavirus spillover into human populations in Africa. Nature Communications.

[CR149] Prathap K, Lau K, Bolton J (1969). Pentastomiasis: a common finding at autopsy among Malaysian aborigines. The American journal of tropical medicine and hygiene.

[CR150] ProMED-mail. (2001). Anthrax, deer, bison, human - USA (Texas), Archive Number: 20010703.1278. ProMED-mail 3 Jul 2001. http://www.promedmail.org/. Accessed 24 Feb 2015.

[CR151] ProMED-mail. (2009). Rabies - Tanzania (Serengeti National Park), civet, human exp., novel lyssavirus, Archive Number: 20120314.1070293. ProMED-mail 14 Mar 2009. http://www.promedmail.org/. Accessed 24 Feb 2015.

[CR152] ProMED-mail. (2011). Anthrax, human, livestock - India (06): (AP) wildlife, Archive Number: 20110802.2328. ProMED-mail 2 Aug 2011. http://www.promedmail.org/. Accessed 24 Feb 2015.

[CR153] ProMED-mail. (2014a). Australian bat lyssavirus - Australia (03): (NS) flying fox, human exp, Archive Number: 20141025.2900858. ProMED-mail 25 Oct 2014. http://www.promedmail.org/. Accessed 25 Feb 2015.

[CR154] ProMED-mail. (2014b). Rabies - Costa Rica: (Puntarenas) human, squirrel, Archive Number: 20140728.2641077. ProMED-mail 28 Jul 2014. http://www.promedmail.org/. Accessed 24 Feb 2015.

[CR155] Rabatsky-Ehr T, Dingman D, Marcus R, Howard R, Kinney A, Mshar P (2002). Deer meat as the source for a sporadic case of *Escherichia coli* O157: H7 infection. Emerging infectious diseases.

[CR156] Rabinowitz P, Conti L (2013). Links Among Human Health, Animal Health, and Ecosystem Health. Annual Review of Public Health.

[CR157] Radakovic M, Fletcher J, Bauer A, Vodnansky M, Winkelmayer R, Smulders FJM (2011). Risk Management of game: from theory to practice. Game meat hygiene in focus: Microbiology, epidemiology, risk analysis and quality assurance, Paulsen P.

[CR158] Ramasoota T (1991). Current status of food-borne parasitic zoonoses in Thailand. Southeast Asian Journal of Tropical Medicine Public Health.

[CR159] Ranque S, Faugère B, Pozio E, La Rosa G, Tamburrini A, Pellissier J-F, Brouqui P (2000). Trichinella pseudospiralis outbreak in France. Emerging infectious diseases.

[CR160] Robson J, Harrison M, Wood R, Tilse M, McKay A, Brodribb T (1993). Brucellosis: re-emergence and changing epidemiology in Queensland. The Medical Journal of Australia.

[CR161] Rodríguez PE, Rodriguez-Ferrer M, Nieto-Martinez J, Ubeira F, Garate-Ormaechea T (2004). Trichinellosis outbreaks in Spain (1990–2001). Enfermedades infecciosas y microbiologia clinica.

[CR162] Roess AA, Galan A, Kitces E, Li Y, Zhao H, Paddock CD, Adem P, Goldsmith CS, Miller D, Reynolds MG (2010). Novel deer-associated parapoxvirus infection in deer hunters. New England Journal of Medicine.

[CR163] Rosenkranz M, Elsner H-A, Stürenburg HJ, Weiller C, Röther J, Sobottka I (2003). Streptococcus suis meningitis and septicemia contracted from a wild boar in Germany. Journal of neurology.

[CR164] Ross RD, Stec LA, Werner JC, Blumenkrank MS, Glazer L, Williams GA (2001). Presumed acquired ocular toxoplasmosis in deer hunters. Retina.

[CR165] Rounds JM, Rigdon CE, Muhl LJ, Forstner M, Danzeisen GT, Koziol BS, Taylor C, Shaw BT, Short GL, Smith KE (2012). Non-O157 Shiga toxin-producing Escherichia coli associated with venison. Emerging infectious diseases.

[CR166] Sacks JJ, Delgado DG, Lobel HO, Parker RL (1983). Toxoplasmosis infection associated with eating undercooked venison. American journal of epidemiology.

[CR167] Samaratunga, Searle, Hudson (1998) Non–rabies Lyssavirus human encephalitis from fruit bats: Australian bat Lyssavirus (pteropid Lyssavirus) infection. *Neuropathology and Applied Neurobiology* 24:331–335. 10.1046/j.1365-2990.1998.00129.x 9775399

[CR168] Scheffers BR, Corlett RT, Diesmos A, Laurance WF (2012). Local demand drives a bushmeat industry in a Philippine forest preserve. Tropical Conservation Science.

[CR169] Schellenberg RS, Tan BJ, Irvine JD, Stockdale DR, Gajadhar AA, Serhir B, Botha J, Armstrong CA, Woods SA, Blondeau JM (2003). An outbreak of trichinellosis due to consumption of bear meat infected with Trichinella nativa in 2 northern Saskatchewan communities. Journal of Infectious Diseases.

[CR170] Schipper J, Chanson JS, Chiozza F, Cox NA, Hoffmann M, Katariya V, Lamoreux J, Rodrigues AS, Stuart SN, Temple HJ (2008). The status of the world’s land and marine mammals: diversity, threat, and knowledge. Science.

[CR171] Schweizer M, Falcone V, Gänge J, Turek R, Neumann-Haefelin D (1997). Simian foamy virus isolated from an accidentally infected human individual. Journal of virology.

[CR172] Seguchi K, Matsuno M, Kataoka H, Kobayashi T, Maruyama H, Itoh H, Koono M, Nawa Y (1995). A case report of colonic ileus due to eosinophilic nodular lesions caused by Gnathostoma doloresi infection. The American journal of tropical medicine and hygiene.

[CR173] Serrano R, Lacasa J, Velázquez J, Ziad F, Aznar R (1989). Trichinosis: new epidemic outbreak caused by the ingestion of wild-boar sausage. Enfermedades infecciosas y microbiologia clinica.

[CR174] Shah KV (1972). Evidence for an SV40-related papovavirus infection of man. American journal of epidemiology.

[CR175] Sharma DS (1999). TRAFFIC report-Tortoise and freshwater turtle trade and utilisation in Peninsular Malaysia.

[CR176] Sharma DSK, Tisen OB (1999) Freshwater Turtle and Tortoise Utilization and Conservation Status in Malaysia. In: *Asian Turtle Trade: Proceedings of a Workshop on Conservation and Trade of Freshwater Turtles and Tortoises in Asia*. Phnom Penh, Cambodia: Chelonian Research Foundation, pp 120–128

[CR177] Shepherd CR, Krishnasamy K (2014). A Review of the Sun Bear Trade in Sarawak, Malaysia. TRAFFIC Bulletin.

[CR178] Smith KJ, Skelton HG, James WD, Lupton GP (1991). Parapoxvirus infections acquired after exposure to wildlife. Archives of dermatology.

[CR179] Starnes C, Talwani R, Horvath J, Duffus W, Bryan C (2004). Brucellosis in two hunt club members in South Carolina. Journal of the South Carolina Medical Association.

[CR180] Summer R, Ross S, Kiehl W (2004). Imported case of rabies in Germany from India. Euro Surveillance.

[CR181] Takahashi K, Kitajima N, Abe N, Mishiro S (2004). Complete or near-complete nucleotide sequences of hepatitis E virus genome recovered from a wild boar, a deer, and four patients who ate the deer. Virology.

[CR182] Tanaka S, Maruyama H, Ishiwata K, Nawa Y (1997). A case report of pleural sparganosis. Parasitology International.

[CR183] Tappe D, Abdullah S, Heo C, Kannan Kutty M, Latif B (2013). Review Paper Human and animal invasive muscular sarcocystosis in Malaysia–recent cases, review and hypotheses. Tropical biomedicine.

[CR184] Tei S, Kitajima N, Ohara S, Inoue Y, Miki M, Yamatani T, Yamabe H, Mishiro S, Kinoshita Y (2004). Consumption of uncooked deer meat as a risk factor for hepatitis E virus infection: An age-and sex-matched case-control study. Journal of medical virology.

[CR185] Tei S, Kitajima N, Takahashi K, Mishiro S (2003). Zoonotic transmission of hepatitis E virus from deer to human beings. The Lancet.

[CR186] Thayaparan S, Robertson I, Amraan F, Suut L, Abdullah M (2013). Serological prevalence of Leptospiral infection in wildlife in Sarawak, Malaysia. Borneo Journal of Resource Science and Technology.

[CR187] Toyoda K, Furusyo N, Takeoka H, Murata M, Sawayama Y, Hayashi J (2008). Epidemiological study of hepatitis E virus infection in the general population of Okinawa, Kyushu, Japan. Journal Of Gastroenterology And Hepatology.

[CR188] Tu Z-C, Zeitlin G, Gagner J-P, Keo T, Hanna BA, Blaser MJ (2004). Campylobacter fetus of reptile origin as a human pathogen. Journal of clinical microbiology.

[CR189] Une Y, Mori T (2007). Tuberculosis as a zoonosis from a veterinary perspective. Comparative Immunology, Microbiology and Infectious Diseases.

[CR190] Vashi NA, Reddy P, Wayne DB, Sabin B (2010). Bat-associated leptospirosis. Journal of general internal medicine.

[CR191] Wang M, Yan M, Xu H, Liang W, Kan B, Zheng B, Chen H, Zheng H, Xu Y, Zhang E (2005). SARS-CoV infection in a restaurant from palm civet. Emerging infectious diseases.

[CR192] Warrilow D, Smith IL, Harrower B, Smith GA (2002). Sequence Analysis of an Isolate from a Fatal Human Infection of Australian Bat Lyssavirus. Virology.

[CR193] Weigler BJ (1992). Biology of B virus in macaque and human hosts: a review. Clinical Infectious Diseases.

[CR194] Weiss RA, McMichael AJ (2004). Social and environmental risk factors in the emergence of infectious diseases. Nature medicine.

[CR195] Wichmann O, Schimanski S, Koch J, Kohler M, Rothe C, Plentz A, Jilg W, Stark K (2008). Phylogenetic and case-control study on hepatitis E virus infection in Germany. Journal of Infectious Diseases.

[CR196] Wilkins M, Bartlett P, Frawley B, O’Brien D, Miller C, Boulton M (2003). Mycobacterium bovis (bovine TB) exposure as a recreational risk for hunters: results of a Michigan Hunter Survey, 2001. The International Journal of Tuberculosis and Lung Disease.

[CR197] Wilkins MJ, Meyerson J, Bartlett PC, Spieldenner SL, Berry DE, Mosher LB, Kaneene JB, Robinson-Dunn B, Stobierski MG, Boulton ML (2008). Human Mycobacterium bovis infection and bovine tuberculosis outbreak, Michigan, 1994–2007. Emerging infectious diseases.

[CR198] Wisser J, Rudolph M, Frolich K, Pilaski J, Strauss G, Meyer H, Burck G, Truyen U (2001). Cowpox virus infection causing stillbirth in an Asian elephant (Elephas maximus). Veterinary Record.

[CR199] Wiwanitkit V (2005). A review of human sparganosis in Thailand. International journal of infectious diseases.

[CR200] Wolfe ND, Heneine W, Carr JK, Garcia AD, Shanmugam V, Tamoufe U, Torimiro JN, Prosser AT, LeBreton M, Mpoudi-Ngole E (2005). Emergence of unique primate T-lymphotropic viruses among central African bushmeat hunters. Proceedings of the National Academy of Sciences.

[CR201] Wolfe ND, Switzer WM, Carr JK, Bhullar VB, Shanmugam V, Tamoufe U, Prosser AT, Torimiro JN, Wright A, Mpoudi-Ngole E, McCutchan FE, Birx DL, Folks TM, Burke DS, Heneine W (2004). Naturally acquired simian retrovirus infections in central African hunters. The Lancet.

[CR202] Xu H, Wang M, Zhang Z, Zou X, Gao Y, Liu X, Lu E, Pan B, Wu S, Yu S (2004). An epidemiologic investigation on infection with severe acute respiratory syndrome coronavirus in wild animals traders in Guangzhou. Zhonghua yu fang yi xue za zhi [Chinese journal of preventive medicine].

[CR203] Yamaguchi T (1991). Present status of trichinellosis in Japan. Southeast Asian Journal of Tropical Medicine Public Health.

[CR204] Yao MH, Wu F, Tang LF (2008). Human pentastomiasis in China: case report and literature review. Journal of Parasitology.

[CR205] Yapo Ette H, Fanton L, Adou Bryn K, Botti K, Koffi K, Malicier D (2003). Human pentastomiasis discovered postmortem. Forensic science international.

[CR206] Ye F, Sheng Z-K, Li J-J, Sheng J-F (2013). Severe pentastomisasis in children: A report of 2 cases. The Southeast Asian Journal of Tropical Medicine Public Health.

[CR207] Yob JM, Field H, Rashdi AM, Morrissy C, van der Heide B, Rota P, bin Adzhar A, White J, Daniels P, Jamaluddin A (2001). Nipah virus infection in bats (order Chiroptera) in peninsular Malaysia. Emerging infectious diseases.

