# Supplementary material for: A Review of Zoonotic Infection Risks Associated with the Wild Meat Trade in Malaysia
Source: Ecohealth. 2017 Mar 22;14(2):361–88. doi: 10.1007/s10393-017-1229-x (PMC5486459; doi:10.1007/s10393-017-1229-x)
Supplement: Supplementary file 1 — Supplementary material 1 (DOCX 304 kb) [file 10393_2017_1229_MOESM1_ESM.docx]

# Supplementary materials: Appendix

1. Potential zoonotic viral pathogens from wildlife involved in the wild meat trade

Legend: PCR= PCR testing, PM= post mortem examination, S= serology, V= virus isolation, G= genetic sequencing, O= other test.

| **WILDLIFE HOST** | | **ZOONOTIC VIRUSES** | | | | | |
| --- | --- | --- | --- | --- | --- | --- | --- |
| **Animal class** | **Taxonomic order or family** | **Viral pathogen** | **Scientific reference and country** | **Sampling method and wildlife host** | **Potential transmission route from wildlife to human** | **Potential zoonotic risk from hunting, butchering or consumption** | **Actual zoonotic risk (scientific evidence)** |
| Mammalia | Suidae | *Hepatitis E virus* | (de Deus et al. 2008) Spain; (Kaba et al. 2010) France; (Kaci et al. 2008) Germany; (Michitaka et al. 2007) & (Sato et al. 2011) Japan. | S of *Sus scrofa*; PM & PCR of *S. scrofa*; S of *S. scrofa*; S of *S. scrofa* for both. | Ingestion of undercooked or raw meat or liver.  Faeco-oral transmission?  (Meng et al. 2009)  Direct contact with infected blood? (Kaba et al. 2010) | Consumption  Hunting & Butchering?  Butchering? | (Li et al. 2005b), (Masuda et al. 2005), (Matsuda et al. 2003) & (Toyoda et al. 2008) Japan; (Wichmann et al. 2008) Germany. |
|  |  | *Swine influenza virus* | (Kaden et al. 2008) Germany; (Saliki et al. 1998) USA; (Vicente Baños et al. 2002) Spain. | S of *S. scrofa;* S of *S. scrofa;* S of *S. scrofa.* | Contact with infected respiratory secretions e.g. inhalation of aerosolised virus. | Hunting & Butchering |  |
| Mammalia | Cervidae | *Hepatitis E virus* | (Matsuura et al. 2007) & (Tomiyama et al. 2009) Japan; (Rutjes et al. 2010) The Netherlands. | S of *Cervus nippon*; S of *C. nippon yesoensis*; S of *Cervus elaphus.* | Ingestion of undercooked or raw meat.  Direct contact with infected animals? (Teo 2010) | Consumption  Hunting? | (Takahashi et al. 2004), (Tei et al. 2003) & (Tei et al. 2004) Japan. |
|  |  | *Parapoxvirus* | (Horner et al. 1987) New Zealand; (Klein and Tryland 2005) Norway. | PM of farmed *C. elaphus*; PCR of *Rangifer terandus.* | Direct contact via damaged skin or wounds. | Hunting & Butchering | (Kuhl et al. 2003), (Roess et al. 2010) & (Smith et al. 1991) USA. |
| Mammalia | Sciuridae | *Lymphocytic choriomeningitis virus* | (Greenwood and Sanchez 2002) UK. | S from PM *Sciurus carolinensis.* | Contact with infected excretions & secretions.  Animal bite transmission. | Hunting & Butchering  Hunting |  |
|  |  | *Rabies (Lyssavirus* | (Cappucci Jr et al. 1972) USA; (Webster et al. 1988) Canada. | PM of Sciurus niger; PM of unspecified squirrel species. | Animal bite transmission.  Non-bite exposure from contact with infected saliva or tissues via mucous membranes or damaged skin. | Hunting  Hunting & Butchering | (Kumari et al. 2014) India; (ProMED-mail 2014b) Costa Rica. |
| Mammalia | Viverridae | *Highly Pathogenic Avian Influenza virus (e.g. H5N1)* | (ProMED-mail 2005) & (ProMED-mail 2008) Vietnam. | PM of captive *Chrotogale owstoni* for both. | Contact with infected respiratory secretions e.g. inhalation of aerosolised virus | Hunting & Butchering |  |
|  |  | *Rabies (Lyssavirus)* | (Bingham 1992) Zimbabwe; (de Fontes-Pereira et al. 2012) Angola; (Matsumoto et al. 2011) Sri Lanka; (Susetya et al. 2008) Indonesia; (ProMED-mail 2014c) Taiwan. | PM of *Civettictis civetta*; PM of *C. civetta*; PM of *Paradoxus zeylonensis*; PCR from PM of unspecified civet; PM of *Paguma larvata taivana.* | Animal bite transmission.  Non-bite exposure from contact with infected saliva or tissues via mucous membranes or damaged skin. | Hunting  Hunting & Butchering | (ProMED-mail 2009) Tanzania. |
|  |  | *SARS Coronavirus* | (Guan et al. 2003), (Shi and Hu 2008) (Tu et al. 2004a) China. | PCR from *Paguma larvata*; PCR from *P. larvata*; S of unspecified civets. | Contact with infectious respiratory droplets via mucous membranes.  Indirect transmission via fomites. | Hunting & Butchering | (Bell et al. 2004), (Wang et al. 2005) & (Xu et al. 2004) China. |
| Mammalia | Caprinae | *Parapoxvirus* | (Inoshima et al. 1999), (Inoshima et al. 2001) & (Suzuki et al. 1993) Japan. | S of *Capricornis crispis* for all. | Direct contact via damaged skin or wounds. | Hunting & Butchering |  |
| Mammalia | Pteropodidae | *Ebola virus e.g. Reston subtype* | (Olival et al. 2013) Bangladesh;  (Taniguchi et al. 2011) Philippines. | S of *Rousettus leschenaultia*; S of *Rousettus amplexicaudatus*. | Fruit bats are likely reservoir hosts with transmission to human via an intermediate host. Could direct transmission from bat to human occur? | Hunting & Butchering? |  |
|  |  | *Lyssaviruses (including rabies)* | (Arguin et al. 2002) The Philippines; (Baer and Smith 1991) Thailand; (Reynes et al. 2004) Cambodia. | S of *Pteropus hypromelanus & R. amplexicaudatus*; S of *Cynopterus brachyotis*; S of PM *Cynopterus sphinx, Pteropus lylei & R. leschenaultia.* | Animal bites transmission.  Non-bite exposure from contact with saliva or infected tissues via mucous membranes or damaged skin. | Hunting  Hunting & Butchering | (Hanna et al. 2000), (Samaratunga et al. 1998), (Warrilow et al. 2002) & (ProMED-mail 2014a) Australia. |
|  |  | *Nipah virus* | (Chua K B 2002), (Epstein et al. 2006) & (Yob et al. 2001) Malaysia; (Hsu et al. 2004) Bangladesh. | V & PCR of *P. hypromelanus*; S of *P.hypromelanus & Pteropus vampyrus*; S of *P.hypromelanus & P. vampyrus*; S of *P.giganteus.* | Ingestion of urine or saliva contaminated food products (could this include bat meat?)  Contact with infected urine or saliva. | Consumption  Hunting & Butchering | (Epstein et al. 2008) India; (Luby et al. 2006) & (Luby et al. 2009) Bangladesh. |
|  |  | *Reoviruruses (e.g. Melaka & Pulau virus)?* | (Chua et al. 2007) & (Pritchard et al. 2006) Malaysia. | G of reovirus isolates from bats & humans; V & G of *P. hypomelanus.* | Could direct transmission from bat to human occur through close contact? | Hunting & Butchering? | (Chua et al. 2007) Malaysia. |
|  |  | *SARS Coronavirus* | (Li et al. 2005c) China; (Müller et al. 2007) South Africa & Democratic Republic of Congo. | S of *R. leschenaultia*; S of *Rousettus aegyptiacus.* | Fruit bats are reservoir hosts with transmission to human via an intermediate host. Could direct transmission from bat to human occur? | Hunting & Butchering? |  |
| Mammalia | Hystricidae | Not reported |  |  |  |  |  |
| Mammalia | Ursidae | *Rabies (Lyssavirus)* | (Bronson et al. 2014) USA; (Mihai et al. 2006) Romania; (Prestrud et al. 1992) Norway; (Taylor et al. 1991) Canada. | S of *Ursus americanus*; PM of *Ursus arctos*; PM of *Ursus maritimus*; PM of *U. maritimus.* | Animal bite transmission.  Non-bite exposure from contact with infected saliva or tissues via mucous membranes or damaged skin. | Hunting  Hunting & Butchering |  |
| Mammalia | Cercopithecidae | *Cercopithecine Herpesvirus* | (Huff and Barry 2003) & (Weigler 1992) both reviews; (Engel et al. 2002) Indonesia. | *S & PCR of Macaca genus (e.g. M. facicularis, M. artoides, M. nemestrina, M. fuscata, M. radiate, M. cyclopis)*; S of *M. fascicularis.* | Animal bite or scratch transmission.  Non-bite exposure from contact with infected saliva or secretions via mucous membranes or damaged skin.  Respiratory transmission? (Weigler 1992).  Indirect transmission via contaminated fomites. | Hunting  Hunting & Butchering  Hunting?  Butchering | (Holmes et al. 1990), (CDC 1987), (CDC 1998) & (Huff and Barry 2003) USA; (Weigler 1992) USA & UK. |
|  |  | *Ebola virus Reston subtype* | (Miranda et al. 1999) Philippines; (Rollin et al. 1999) USA. | S of captive *M. fascicularis*; S & V of captive *M. fascicularis.* | Contact with infected animals, body fluids & tissues. | Hunting & Butchering | (Miranda et al. 1999), (Morikawa et al. 2007) USA & Philippines. |
|  |  | *Rabies virus (Lyssavirus)* | (Susetya et al. 2008) Indonesia; (Wilde et al. 1991) Thailand. | PM of unspecified monkey species; PM of *Macaca mulatta.* | Transmission from animal bites.  Non-bite exposure from contact with infected animal saliva or tissues via mucous membranes or damaged skin. | Hunting  Hunting & Butchering | (Favoretto et al. 2001) Brazil; (Summer et al. 2004) India. |
|  |  | *Simian Foamy Virus* | (Jones-Engel et al. 2006) Nepal; (Jones-Engel et al. 2007) Thailand & Singapore; (Leendertz et al. 2008) Cote d’Ivoire. | S of *M. mulatta*; S of *M. fascicularis*, *M. arctoides*, *M. mulatta/fascicularis* hybrid, *M. assamensis* & *M. nemestrina*; PCR of *Piliocolobus badius.* | Direct contact with infected saliva from bites, scratches and mucosal splashes. | Hunting & Butchering | (Brooks et al. 2002) Canada; (Huang et al. 2012) China; (Jones-Engel et al. 2005) Indonesia; (Jones-Engel et al. 2008) several Asian countries; (Schweizer et al. 1997) Germany; (Wolfe et al. 2004) Cameroon. |
|  |  | *Simian Type D Retrovirus* (reported in captive macaques) | (Murphy et al. 2006) USA. | S & PCR of captive *Macaca genus.* | Direct contact with infected saliva from bites, scratches and mucosal splashes? | Hunting & Butchering? | (Lerche et al. 2001) USA. |
|  |  | *Simian Virus 40* | (Butel and Lednicky 1999) Asia; (Jones-Engel et al. 2006) Nepal. | S of Asian *Macaca species*; S of *M. mulatta.* | Direct contact with infected animal saliva from bites, scratches and mucosal splashes. | Hunting & Butchering | (Engels et al. 2004) North America; (Shah 1972) India. |
| Mammalia | Felidae | *Highly Pathogenic Avian Influenza virus (e.g. H5N1)* | (Desvaux et al. 2009) Cambodia; (Keawcharoen et al. 2004) & (Thanawongnuwech et al. 2005) Thailand. | S of captive *Panthera tigris, Pantera leo, Panthera pardus, Catopuma temminckii & Neofelis nebulosa*; PM of *P. tigris & P. pardus*; PM of *P. tigris* | Contact with infected respiratory secretions e.g. inhalation of aerosolised virus (Reperant et al. 2009). | Hunting & Butchering |  |
|  |  | *Rabies virus (Lyssavirus)* | (Pandit 1950) India; (Susetya et al. 2008) Indonesia; (Wilde et al. 1991) Thailand. | PM of *P. tigris*; PCR from PM of *P. tigris*; PM of *P. tigris.* | Animal bite transmission.  Non-bite exposure from contact with infected saliva or tissues via mucous membranes or damaged skin. | Hunting  Hunting & Butchering | (Pandit 1950) India. |
| Mammalia | Manidae | Not reported |  |  |  |  |  |
| Mammalia | Elephantidae | *Cowpox virus* (reported in captive elephants) | (Kik et al. 2009) The Netherlands; (Wisser et al. 2001) Germany. | PCR of captive *Elaphus maximus*; PM of captive *E. maximus.* | Direct contact via damaged skin, bites & scratches. | Hunting & butchering | (Hemmer et al. 2010) & (Kurth et al. 2008) Germany. |
| Reptilia | Squamata | Not reported |  |  |  |  |  |
| Reptilia | Testudines | Not reported |  |  |  |  |  |
| Reptilia | Crocodylia | Not reported |  |  |  |  |  |
| Aves | Galliformes | *Avian paramyxovirus-1 (Newcastle disease)* | (Hopkins et al. 1990) USA; (Lee and Amin-Babjee 1990) Malaysia; (Roy et al. 1998) India. | S of *Meleagridis gallopavo silvestris* PM of *Gallus* gallus; V of *G. gallus &Chrysolophus pictus.* | Contact with large amounts of virus from infected birds or their carcasses e.g. inhalation. | Hunting & Butchering |  |
|  |  | *Highly Pathogenic Avian Influenza* | (Desvaux et al. 2009) Cambodia; (Stallknecht et al. 2008) & (Swayne and Suarez 2000) reviews. | V of *G. gallus*; V of *Phasianus colchicus & Alectoris graeca*; V of *Numida meleagris, Colinus virginianus, Phasianus colchicus, Coturnix japonica.* | Contact with infected respiratory secretions e.g. inhalation of aerosolised virus. | Hunting & Butchering |  |

1. Potential zoonotic bacterial pathogens from wildlife involved in the wild meat trade

Legend: F= faecal sampling, PCR= PCR testing, PM= post mortem examination, S= serology, O= other test.

| **WILDLIFE HOST** | | **ZOONOTIC BACTERIA** | | | | | |
| --- | --- | --- | --- | --- | --- | --- | --- |
| **Animal class** | **Taxonomic order or family** | **Bacterial pathogen** | **Scientific reference and country (when known)** | **Sampling method and wildlife host** | **Potential transmission route from wildlife to human** | **Potential zoonotic risk from hunting, butchering or consumption** | **Actual zoonotic risk (scientific evidence)** |
| Mammalia | Suidae | *Bacillus anthracis* | (Bagamian et al. 2014) Ukraine; (Wafula et al. 2008) Uganda. | S of *Sus scrofa*; PM of *Phacochoerus africanus.* | Ingestion of raw or undercooked meat or tissues.  Contact with contaminated animal products (e.g. fur, hide, bone) via broken skin.  Inhalation of spores (rare). | Consumption  Butchering  Butchering | (ProMED-mail 2011) India. |
|  |  | *Brucella spp* | (Al Dahouk et al. 2005) Germany; (Cvetnic et al. 2003) Croatia; (Godfroid et al. 1994) Belgium; (Gresham et al. 2002) USA; (Irwin et al. 2010) Australia; (Watarai et al. 2006) Japan. | S of *S. scrofa* for all. | Ingestion of raw or undercooked meat or tissues.  Direct contact with infected body fluids or tissues via broken skin or mucous membranes. | Consumption  Hunting & Butchering | (Carrington et al. 2012), (Giurgiutiu et al. 2009) & (Starnes et al. 2004) USA; (Eales et al. 2010), (Massey et al. 2011) & (Robson et al. 1993) Australia; (Garin-Bastuji et al. 2006) France. |
|  |  | *Campylobacter spp* | (Carbonero et al. 2014) & (Navarro-Gonzalez et al. 2013) Spain; (Jay and Wiscomb 2008) USA. | F from *S. scrofa*; F from PM of *S. scrofa*; F from *S. scrofa.* | Ingestion of raw or undercooked meat or tissues.  Faeco-oral transmission. | Consumption  Hunting & Butchering |  |
|  |  | *Chlamydophilia spp* | (Hotzel et al. 2004) Germany; (Salinas et al. 2009) Spain. | PCR from PM of *S. scrofa*; S of *S. scrofa.* | Aerogenous or cutaneous  transmission via infected secretions or excretions. | Hunting or Butchering |  |
|  |  | *Erysipelothrix rhusiopathiae* | (Risco et al. 2011) & (Vicente Baños et al. 2002) Spain. | PM of *S.* scrofa; S from PM of *S. scrofa.* | Ingestion of raw or undercooked meat (Kanai et al. 1997).  Direct contact with animal products via damaged skin. | Consumption  Butchering | (Addidle et al. 2009) New Zealand. |
|  |  | *Escherichia coli (Shiga toxin producing e.g. O157 and others)* | (Jay et al. 2007) USA; (Sánchez et al. 2010) Spain; (Schierack et al. 2009) Germany. | F of *S. scrofa*; PM of *S. scrofa*; F of *S. scrofa.* | Ingestion of raw or undercooked meat or tissues (Gill 2007) & (Miko et al. 2009).  Faecal-oral transmission. | Consumption  Hunting & Butchering |  |
|  |  | *Francisella tularensis* | (Al Dahouk et al. 2005) Germany; (Hubálek et al. 2002) Czech Republic. | S of *S. scrofa*; S from PM of *S. scrofa.* | Ingestion of raw or undercooked meat or tissues.  Direct contact with infected animals via mucous membranes or broken skin.  Inhalation of aerosolised bacteria. | Consumption  Hunting & Butchering  Butchering | (Deutz et al. 2002) Austria;  (Esmaeili et al. 2014) Iran. |
|  |  | *Leptospira spp* | (Ebani et al. 2003) Italy; (Jansen et al. 2007) Germany; (Saliki et al. 1998) USA; (Vicente Baños et al. 2002) Spain. | S of *S. scrofa*; S & PM of *S. scrofa*; S of *S. scrofa*; S of *S. scrofa.* | Ingestion of urine contaminated meat.  Contact with infected urine or reproductive fluids via skin or mucous membranes. | Consumption  Hunting & Butchering |  |
|  |  | *Mycobacterium bovis* | (Vicente et al. 2006) Spain; (Nugent et al. 2012) & (Wakelin and Churchman 1991) New Zealand; (Zanella et al. 2008) France. | PM of *S. scrofa*; PM of *S. scrofa* for both; PM of *S. scrofa*. | Ingestion of raw or undercooked meat or tissues.  Inhalation of aerosolised bacteria.  Direct contact via damaged skin or wounds. | Consumption  Hunting & Butchering  Hunting & Butchering |  |
|  |  | *Salmonella spp* | (Bensink et al. 1991) & (Ward et al. 2013) Australia; (Thakur et al. 2011) USA; (Vengust et al. 2006) Slovenia; (Vicente et al. 2006) Spain; (Vieira-Pinto et al. 2011) Portugal. | PM of *S. scrofa* for both; F from PM of *S. scrofa*; S of *S. scrofa*; S from PM of *S. scrofa*; F of *S. scrofa*. | Ingestion of raw or undercooked meat or tissues (Kanai et al. 1997).  Faecal-oral transmission. | Consumption  Hunting & Butchering |  |
|  |  | *Streptococcus suis* | (Baums et al. 2007) Germany; (Higgins et al. 1997) Canada. | PM of *S. scrofa*; PM of *S. scrofa.* | Direct contact via damaged skin or wounds. | Hunting & Butchering | (Dalsjö et al. 2014) Sweden; (Halaby et al. 2000) The Netherlands; (Rosenkranz et al. 2003) Germany. |
|  |  | *Yersinia pseudotuberculosis*  (NB More likely in temperate rather than tropical climates) & *Y. enterocolitica* | (Al Dahouk et al. 2005) Germany; (Fredriksson-Ahomaa et al. 2009) Switzerland; (Hayashidani et al. 2002) Japan. | S of *S. scrofa;* PM of *S. scrofa;* PM of *S. scrofa.* | Ingestion of raw or undercooked meat or tissues. | Consumption |  |
| Mammalia | Cervidae | *Bacillus anthracis* | (Fasanella et al. 2007) Italy; (Mehrotra et al. 2000) India; (Mongoh et al. 2008) USA; | PM of *Cervus elaphus*; PM of *Axis axis*; PCR of *Odocoileus virginianus & Cervus Canadensis.* | Ingestion of raw or undercooked meat or tissues.  Contact with contaminated animal products (e.g. fur, hide, bone) via broken skin.  Inhalation of spores (rare). | Consumption  Butchering  Butchering | (Ichhpujani et al. 2004) India; (ProMED-mail 2001) USA; (Fasanella et al. 2007) Italy. |
|  |  | *Brucella spp* | (Forbes 1991) & (Tessaro 1986) Canada; (Kim et al. 2014) South Korea; (Muñoz et al. 2010) Spain. | PM of *Rangifer tarandus* subspp*,* PM & S of *C. Canadensis*; S of *Hydropotes inermis* & introduced *Capreolus capreolus*; S of *C. Elaphus.* | Ingestion of raw or undercooked meat or tissues.  Direct contact with infected body fluids or tissues via broken skin or mucous membranes. | Consumption  Hunting & Butchering | (Brody et al. 1966) Alaska, USA; (Chan et al. 1989) Arctic region; (Forbes 1991) Canada; (Meyer 1966) Alaska, Canada & Russia. |
|  |  | *Campylobacter spp* | (Carbonero et al. 2014) Spain; (Khoshbakht et al. 2014) Iran. | F from *C. elpahus*; F from *Dama mesopotamica* | Ingestion raw or undercooked meat or tissues.  Faeco-oral transmission. | Consumption  Hunting & Butchering. |  |
|  |  | *Chlamydophilia spp* | (Cubero-Pablo et al. 2000) & (Salinas et al. 2009) Spain; (Di Francesco et al. 2012) Italy. | S of *C. elaphus & Dama dama*; S of *C. elaphus, D. dama & C. capreolus*; S of *C. elaphus.* | Aerogenous or cutaneous  transmission via infected secretions or excretions. | Hunting & Butchering |  |
|  |  | *Erysipelothrix rhusiopathiae* | (Bruner et al. 1984) USA; (Eskens and Zschock 1991) Germany. | PM of *O. virginianus;* PM of *C. capreolus.* | Ingestion of raw or undercooked meat (Kanai et al. 1997).  Direct contact with animal products via damaged skin. | Consumption  Butchering |  |
|  |  | *Escherichia coli Shiga toxin producing (e.g. O157 & others)* | (Asakura et al. 1998) Japan; (Eggert et al. 2013) Germany; (García-Sánchez et al. 2007) & (Sánchez et al. 2009) Spain; (Obwegeser et al. 2012) Switzerland; (Renter et al. 2001) USA. | F from unspecified wild deer; F and PM of *C. elaphus* & *C. capreolus*; PM of *C. elaphus, C. capreolus* & *D. dama* for both; F for PCR of *C. elaphus* & *C. capreolus*; F of *O. virginianus.* | Ingestion of raw or undercooked meat or tissues (Gill 2007) &  (Miko et al. 2009).  Faecal-oral transmission. | Consumption  Hunting & Butchering | (Keene et al. 1997), (Rabatsky-Ehr et al. 2002) & (Rounds et al. 2012) USA; (Nagano et al. 2004) Japan. |
|  |  | *Leptospirosis spp* | (Ayanegui-Alcerreca et al. 2007) New Zealand; (Bender and Hall 1996) USA; (Uhart et al. 2003) Argentina. | S & PM of *C. elaphus, Cervus nippon, D. dama, C. canadensis*; S of *C. canadensis*; S of *Ozotoceros bezoarticus celer*. | Ingestion of urine contaminated meat.  Contact with infected urine via skin or mucous membranes. | Consumption  Hunting or Butchering | (Brown 2005) New Zealand. |
|  |  | *Mycobacterium bovis* | (Baviskar and Bhandarkar 2010) India; (Delahay et al. 2001) UK; (Pavlik et al. 2002) Czech Republic & Hungary; (Martín-Hernando et al. 2010) Spain; (Rhyan and Saari 1995) & (Schmitt et al. 1997) USA. | PM of *Muntiacus muntjak*; PM of *M*. *muntjak*; PM of *C. elaphus*; PM of *C. elaphus elaphus, D. dama;* PM of *D. dama, C. nippon, C. elaphus elaphus, Cervus elpahus nelsoni*; PM of *O. virginianus.* | Ingestion of raw or undercooked meat or tissues.  Inhalation of aerosolised bacteria.  Direct contact via damaged skin or wounds. | Consumption  Hunting & Butchering  Hunting & Butchering | (Baker et al. 2006) New Zealand; (Fanning and Edwards 1991), (Liss et al. 1993) & (Nation et al. 1999) Canada; (Wilkins et al. 2003) (Wilkins et al. 2008) USA. |
|  |  | *Salmonella spp* | (Aschfalk et al. 2002) Norway; (Branham et al. 2005) & (Renter et al. 2006) USA; (Sato et al. 2000) Japan. | S of *Alces alces*; PM of *O. virginianus*; F from *O. virginianus*; PM of *C. nippon.* | Ingestion of raw or undercooked meat or tissues.  Faecal-oral transmission. | Consumption  Hunting & Butchering | (Kuhn et al. 2011) Denmark; (Madar et al. 2012) Hawaii. |
|  |  | *Yersinia pestis* | (ProMED-mail 2003) USA. | PM of *O. hemionus* | Contact with infected secretions or tissues via bites, scratches, damaged skin or mucous membranes.  Inhalation of aerosolised bacteria. | Hunting & Butchering  Butchering |  |
|  |  | *Yersinia pseudotuberculosis*  (NB More likely in temperate rather than tropical climates) and *Y. enterocolitica* | (Aschfalk et al. 2008) Norway; (Henderson 1984) New Zealand; (Fukushima and Gomyoda 1991) Japan. | F of *C. elaphus*; F of *C. elaphus, D. dama & C. Canadensis*; F of *C. nippon* | Ingestion of raw or undercooked meat or tissues. | Consumption |  |
| Mammalia | Sciuridae | *Francisella tularensis* | (Wobeser et al. 2009) Canada. | S and PM of Spermophilus richardsonii & Spermophilus franklinii. | Ingestion of raw or undercooked meat or tissues.  Direct contact with infected animals via mucous membranes or broken skin.  Inhalation of aerosolised bacteria. | Consumption  Hunting & Butchering  Butchering | (Bow and Brown 1946) Canada; (Magee et al. 1989) USA. |
|  |  | *Leptospira spp.* | (Dirsmith et al. 2013) USA; (Gozzi et al. 2013) Argentina; (Montes et al. 2011) Peru; (Thayaparan et al. 2013) Malaysia. | PM of *Sciurus niger*; PM of introduced *Callosciurus erythraeus*; S of *Sciurus stramineus*; S of *Callosciurus notatus.* | Ingestion of urine contaminated meat.  Contact with infected urine via skin & mucous membranes. | Consumption  Hunting & Butchering | (Diesch et al. 1967) USA;  (Masuzawa et al. 2006) Japan. |
|  |  | *Yersinia pestis* | (Cui et al. 2008) China;  (Stevenson et al. 2003) & (ProMED-mail 2013) USA; (Wobeser et al. 2009) Canada. | PM of *Marmota himalayana*;  PM of *Cynomis ludovicianus*; PM of Spermophilus beecheyi; PM of Spermophilus species, not reported which ones. | Contact with infected secretions or tissues via bites, scratches, damaged skin or mucous membranes.  Inhalation of aerosolised bacteria. | Hunting & Butchering  Butchering | (Li et al. 2005a) China. |
| Mammalia | Viverridae | *Bartonella henselae* | (Sato et al. 2013) Japan. | PM of *Paguma larvata.* | Animal bite or scratch transmission. | Hunting | (Miyazaki et al. 2001) Japan. |
|  |  | *Campylobacter spp* | (Lee et al. 2011b) Japan. | F from PM of *P. larvata.* | Ingestion of raw or undercooked meat or tissues.  Faeco-oral transmission. | Consumption  Hunting & Butchering |  |
|  |  | *Salmonella spp* | (Lee et al. 2011b) Japan. | F from PM of *P. larvata.* | Ingestion of raw or undercooked meat or tissues.  Faecal-oral transmission. | Consumption  Hunting & Butchering |  |
|  |  | *Streptococcus dysgalactiae* | (Li et al. 2012) China. | PM of *P. larvata.* | Direct contact with infected animals or carcasses, often via broken skin. | Hunting & Butchering |  |
|  |  | *Yersinia pseudotuberculosis* | (Lee et al. 2011b) Japan. | F from PM of *P. larvata.* | Ingestion of raw or undercooked meat or tissues. | Consumption |  |
| Mammalia | Caprinae | *Brucella spp* | (Ferroglio et al. 1998) Italy; (Garin-Bastuji et al. 1990) France; (Wazed Ali Mollah and McKinney 2002) UAE. | S of *Capra ibex*; S of *Rupicapra rupicapra*; PM of captive *capra ibex nubiana.* | Ingestion of raw or undercooked meat or tissues.  Direct contact with infected body fluids or tissues via broken skin or mucous membranes. | Consumption  Hunting & Butchering |  |
|  |  | *Campylobacter spp* | (Carbonero et al. 2014) Spain | F from *Ovis mussimon.* | Ingestion of raw or undercooked meat.  Faeco-oral transmission. | Consumption  Hunting & Butchering |  |
|  |  | *Chlamydophilia spp* | (Cubero-Pablo et al. 2000) & (Salinas et al. 2009) Spain; (Kinjo and Minamoto 1987) Japan. | S of *O. mussimon & Capra pyrenaica*; S of *O. mussimon*; *C pyrenaica & R. pyrenaica*; S of *Capricornis crispis.* | Aerogenous or cutaneous  transmission via infected secretions or excretions. | Hunting & Butchering |  |
|  |  | *Escherichia coli Shiga toxin producing* | (Obwegeser et al. 2012) Switzerland. | F for PCR of *C. ibex* | Ingestion of raw or undercooked meat or tissues.  Faecal-oral transmission. | Consumption  Hunting & Butchering |  |
|  |  | *Leptospira spp* | (Kinjo and Minamoto 1987) Japan. | S of *C. crispis.* | Ingestion of urine contaminated meat.  Contact with infected urine via skin & mucous membranes. | Consumption  Hunting & Butchering |  |
|  |  | *Mycobacterium bovis* | (Pavlik et al. 2002) Czech Republic. | PM of *Capra aegagrus.* | Ingestion of raw or undercooked or meat or tissues.  Inhalation of aerosolised bacteria.  Direct contact via damaged skin or wounds. | Consumption  Hunting & Butchering  Hunting & Butchering |  |
|  |  | *Salmonella spp* | (Billinis 2013) Europe; (Pioz et al. 2008) France. | PM of *Ammotragus lervia*; S of *R. rupicapra.* | Ingestion of raw or undercooked meat or tissues.  Faecal-oral transmission. | Consumption  Hunting & Butchering |  |
| Mammalia | Pteropodidae | *Leptospira spp* | (Cox et al. 2005) & (Smythe et al. 2002) Australia; (Thayaparan et al. 2013) Malaysia. | PCR of *Pteropus conspicillatus, P. alecto, P. poliocephalus & P. scapulatus*; S of *P. conspicillatus, P. alecto, P. poliocephalus & P. scapulatus*; S of *Cynopterus brachyotis & Penthetor lucasi.* | Ingestion of urine contaminated meat.  Contact with via skin & mucous membranes. | Consumption  Hunting & Butchering | (Vashi et al. 2010) USA. |
|  |  | *Salmonella spp* | (Mühldorfer 2013) Madagascar. | PM of subfamily *Pteropodinae spp.* | Ingestion of raw or uncooked meat or tissues.  Faecal-oral transmission. | Consumption  Hunting & Butchering |  |
|  |  | *Yersinia pseudotuberculosis* | (Childs-Sanford et al. 2009) USA. | PM of captive *Rousettus aegyptiacus.* | Ingestion of raw or undercooked meat. | Consumption |  |
| Mammalia | Hystricidae | *Leptospira spp* | (Bahaman and Ibrahim 1988), (Kadir et al. 2012) & (Smith et al. 1961) Malaysia. | S of *Atherrurus macrourus*; S of *Hystrix brachyuran*; S of *A. macrourus.* | Ingestion of urine contaminated meat.  Contact with infected urine via skin & mucous membranes. | Consumption  Hunting & Butchering |  |
| Mammalia | Ursidae | *Brucella spp* | (Chomel et al. 1998) & (O'Hara et al. 2010) USA; (Tryland et al. 2001) Norway. | S of *Ursus arctos & Ursus americanus*; S of *Ursus maritimus*; S of *U. maritimus.* | Ingestion of raw or undercooked meat or tissues.  Direct contact with infected body fluids or tissues via broken skin or mucous membranes. | Consumption  Hunting & Butchering |  |
|  |  | *Francisella tularensis* | (Chomel et al. 1998) USA; (Hotta et al. 2012) Japan. | S of *U. arctos & U. americanus*; S of *Ursus thibetanus japonicas.* | Ingestion of raw or undercooked meat or tissues.  Direct contact with infected animals via mucous membranes or broken skin.  Inhalation of aerosolised bacteria. | Consumption  Hunting & Butchering  Butchering | (Chase et al. 1980) USA. |
|  |  | *Leptospira spp* | (Bronson et al. 2014) & (Zarnke 1983) USA;  (Modric and Huber 1993) & (Slavica et al. 2010) Croatia. | S of *U. americanus*; S of *U. arctos*; S of *U. arctos*; S of *U. arctos.* | Ingestion of urine contaminated meat.  Contact with infected urine via skin & mucous membranes. | Consumption  Hunting & Butchering | (Anderson et al. 1978) USA. |
|  |  | *Yersinia pestis* | (Clover et al. 1989) & (Ruppanner et al. 1982) USA. | S of *U. americanus* for both. | Contact with infected secretions or tissues via bites, scratches, damaged skin or mucous membranes.  Inhalation of aerosolised bacteria. | Hunting & Butchering  Hunting & Butchering |  |
| Mammalia | Cercopithecidae | *Campylobacter spp* | (Andrade et al. 2007) Brazil; (Kalashnikova et al. 2002) Russia; (Sestak et al. 2003) USA; (Tribe and Fleming 1983) UK. | F from captive *Macaca mulatta* for first 2 studies; F from captive *M. mulatta & M. fascicularis*; F from captive *M. fascicularis.* | Ingestion of raw or undercooked or raw meat or tissues.  Faeco-oral transmission. | Consumption  Hunting & Butchering |  |
|  |  | *Leptospira spp* | (Thayaparan et al. 2013) Malaysia. | S of captive & free-ranging monkeys including *M. nemestrina.* | Ingestion of urine contaminated meat.  Contact with infected urine via skin & mucous membranes. | Consumption  Hunting & Butchering |  |
|  |  | *Mycobacterium avium* | (Goodwin et al. 1988) USA; (Singh et al. 2011) India. | PM of captive *M. mulatta & M. fascicularis*; PM of wild *M. mulatta.* | Ingestion of contaminated meat.  Faeco-oral transmission. | Consumption  Hunting & Butchering |  |
|  |  | *Mycobacterium bovis or M. tuberculosis* | (Hasselschwert and Ostrowski 1999) & (Janssen et al. 1989) USA; (Wilbur et al. 2012) Gibraltar, Indonesia, Nepal, Singapore, Thailand. | PM of captive *M. fascicularis*; PM of *Macaca thibetans*; O of 11 different *Macaca* species (captive and wild) | Ingestion of raw or undercooked meat or tissues.  Inhalation of aerosolised bacteria.  Direct contact via damaged skin or wounds. | Consumption  Hunting & Butchering  Hunting & Butchering | (Une and Mori 2007) Japan. |
|  |  | *Salmonella spp* | (Tribe and Fleming 1983) UK. | F from captive *M. fascicularis.* | Ingestion of raw or undercooked meat or tissues.  Faecal-oral transmission. | Consumption  Hunting & Butchering |  |
|  |  | *Shigella spp* (reported in captive monkeys) | (Lee et al. 2011a) Japan; (Tribe and Fleming 1983) UK. | F from captive *M. mulatta*; F from captive *M. fascicularis.* | Ingestion of faecal contaminated meat.  Faeco-oral transmission. | Consumption  Hunting & Butchering | (Kennedy et al. 1993) UK. |
| Mammalia | Felidae | *Bartonella henselae* | (Chomel et al. 2004), (Rotstein et al. 2000) & (Yamamoto et al. 1998) USA; (Molia et al. 2004) Kenya, South Africa & Tanzania. | S of wild and captive *Puma concolor & Lynx rufus*; S of wild *P. concolor subspecies*; S of 26 wild felid species & subspecies (wild and captive); S of wild *Panthera leo & Acinonyx jubatus.* | Animal bite or scratch transmission. | Hunting |  |
|  |  | *Leptospira spp* | (Labelle et al. 2000) Canada; (Millán et al. 2009) Spain; (Ullmann et al. 2012) Brazil. | S of *Lynx Canadensis and L. rufus*; S of *Lynx pardinus*; S of *Leopardus pardalis & Leopardus wiedii..* | Ingestion of urine contaminated meat.  Contact with infected urine via skin & mucous membranes. | Consumption  Hunting & Butchering |  |
|  |  | *Pasteurella spp* | (Saxena et al. 2006) India; (Woolfrey et al. 1985) USA. | PCR of *Panthera tigris*; O of *P. tigris.* | Animal bite transmission. | Hunting | (Capitini et al. 2002) & (Durazo and Lessenger 2006) USA; (Isotalo et al. 2000) Canada. |
|  |  | *Yersinia pestis* | (Salkeld and Stapp 2006) & (ProMED-mail 2010a) USA. | S of *L. rufus, L. canadensis & Felis cougar*; PM of *Puma concolor.* | Contact with infected secretions or tissues via bites, scratches, damaged skin or mucous membranes.  Inhalation of aerosolised bacteria. | Hunting & Butchering  Butchering |  |
| Mammalia | Manidae | Not reported |  |  |  |  |  |
| Mammalia | Elephantidae | *Bacillus anthracis* | (Priya et al. 2009) & (Yasothai and Shamsudeen 2014) India | PM of *Elephas maximus* for both. | Ingestion of raw or undercooked meat or tissues.  Contact with contaminated animal products (e.g. fur, hide, bone) via broken skin.  Inhalation of spores (rare). | Consumption  Butchering  Butchering |  |
|  |  | *Mycobacterium spp* (*M. tuberculosis* reported in captive elephants) | (Angkawanish et al. 2010) Thailand; (Mikota et al. 2001) USA; (Ong et al. 2013) Malaysia; (Sarma et al. 2006) India; (Obanda et al. 2013) Kenya; (ProMED-mail 2010b) Nepal. | PM of captive *E. maximus*; PM of captive *E. maximus*; S & PCR of captive *E. maximus*; O & PM of captive *E. maximus*; PM of wild *Loxodonta Africana*; PM of free-ranging and captive *E. maximus*. | Ingestion of raw or undercooked meat or tissues.  Inhalation of aerosolised bacteria.  Direct contact via damaged skin or wounds. | Consumption  Hunting & butchering  Hunting & butchering | (Michalak et al. 1998) & (Murphree et al. 2011) USA. |
| Reptilia | Squamata | *Campylobacter spp (e.g. C. fetus)* | (Gilbert et al. 2014) The Netherlands; (Wang et al. 2013a) Taiwan. | F from a variety of captive lizards and snakes; F from a variety of captive & wild Squamata. | Ingestion of raw or undercooked meat or tissues.  Faeco-oral transmission. | Consumption  Hunting & Butchering | (Patrick et al. 2013) USA. |
|  |  | *Dermatophilus congolensis* | (Montali et al. 1975) & (Wellehan et al. 2004) USA. | O of captive *Pogona barbata*; O of captive *Ophiophagus hannah.* | Direct contact with infected reptile skin. | Hunting & Butchering |  |
|  |  | *Edwardsiella tarda* | (Ferreira Junior et al. 2009) Brazil; (Koeboelkuti et al. 2013) Romania. | O of wild and captive *Crotalus durissus terrificus*; O of captive *Natrix natrix.* | Ingestion of contaminated meat.  Faeco-oral transmission.  Transcutaneous transmission via a wound. | Consumption  Hunting & Butchering for both |  |
|  |  | *Salmonella spp* | (Briones et al. 2004) Spain; (Burnham et al. 1998) USA;(Kuroki et al. 2013) Japan. | F from wild snakes & lizards including *Elaphe scalaris, Malpolon monspessulannus, Tarentola mauritanica Timon lepida*; F from captive *Iguana iguana*; F of 6 different species of snakes. | Ingestion of raw or undercooked meat.  Faeco-oral transmission.  Animal bite and scratch transmission. | Consumption  Hunting & Butchering  Hunting | (Bhatt et al. 1989, Kelly et al. 1995, Friedman et al. 1998, Corrente et al. 2006) USA. |
| Reptilia | Testudines | *Campylobacter spp (e.g. C. fetus)* | (Gilbert et al. 2014) The Netherlands;(Wang et al. 2013b) Taiwan. | F from a variety of captive testudines; F from a variety of captive & wild testudines, including *Trachemys scripta elegans, Emydidae sp.* | Ingestion of raw or undercooked meat.  Faeco-oral transmission. | Consumption  Hunting & Butchering | (Patrick et al. 2013) & (Tu et al. 2004b) USA. |
|  |  | *Dermatophilus congolensis* | (Bemis et al. 1999) USA. | O of captive tortoises *Chersina angulate & Testudo kleinmanni.* | Direct contact with infected reptile skin. | Hunting & Butchering |  |
|  |  | *Edwardsiella tarda* | (Cai et al. 1997) & (Yan et al. 2013) China; (Ferronato et al. 2009) Brazil; (Otis and Behler 1973) USA. | PM of *Trionyx sinensis* for both; O of wild *Phrynops geoffroanus*; F from 8 different captive turtle species. | Ingestion of contaminated meat.  Faeco-oral transmission.  Transcutaneous transmission via a wound. | Consumption  Hunting & Butchering for both | (Nagel et al. 1982) USA. |
|  |  | *Salmonella spp* | (Briones et al. 2004) & (Hidalgo-Vila et al. 2007) Spain. | F from wild tortoises including *Testudo graeca & T. marginata*; F from wild *Testudo graeca, Emys orbicularis & Mauremys leprosa.* | Ingestion of raw or undercooked meat.  Faeco-oral transmission.  Animal bite and scratch transmission. | Consumption  Hunting & Butchering.  Hunting | (Fukushima et al. 2008) Japan.  (Harris et al. 2009) USA. |
| Reptilia | Crocodylia | *Dermatophilus congolensis* | (Buenviaje et al. 1998) Australia. | O of captive *Crocodylus porosus.* | Direct contact with infected animal skin. | Hunting & Butchering |  |
|  |  | *Edwardsiella tarda* | (Charruau et al. 2012) Mexico; (Johnston et al. 2010) USA. | O of *Crocodylus acutus & C. moreletii*; F from *Alligator mississippiensis.* | Ingestion of contaminated meat.  Faeco-oral transmission.  Transcutaneous transmission via a wound. | Consumption  Hunting & Butchering for both. |  |
|  |  | *Salmonella spp* | (Huchzermeyer 1991) South Africa; (Manolis et al. 1991) Australia. | F from captive *Crocodylus niloticus* PM of captive *Crocodylus johnstoni and C. porosus.* | Ingestion of raw or undercooked meat (Magnino et al. 2009).  Faeco-oral transmission.  Animal bite and scratch transmission. | Consumption  Hunting & Butchering  Hunting |  |
| Aves | Galliformes | *Campylobacter spp* | (Díaz-Sánchez et al. 2012) Spain; (Taema et al. 2008) UK; (Tresierra-Ayala et al. 2006) Peru; (Yogasundram et al. 1989) USA. | F from captive & wild *Alectoris rufa*; F from captive *Gallus gallus*; F from wild galliformes; PM of variety of galliformes including *G. gallus,* *Phasianus colchicus, Pavo cristanus.* | Ingestion of raw or undercooked meat.  Faeco-oral transmission. | Consumption  Hunting & Butchering |  |
|  |  | *Chlamydia psittaci* | (Trávniček et al. 2000) Slovakia; (Vanrompay et al. 1993) USA. | S of captive *P. colchicus*; S of a variety of galliformes including *P. colchicus.* | Inhalation of aerosolised bacteria from infected respiratory secretions or dried faeces. | Hunting & Butchering  NB A risk for immune-compromised humans |  |
|  |  | *Erysipelothrix rhusiopathiae* | (Hennig et al. 2002) & (Mutalib et al. 1995) USA; (Pettit et al. 1976) Canada. | PM for PCR of captive *P. colchicus*; PM of captive *Coturnix japonica*; PM of captive *Alectoris graeca.* | Direct contact with animal products via damaged skin or wounds. | Hunting & Butchering | (Mutalib et al. 1995) USA. |
|  |  | *Mycobacterium spp (e.g. M.avium, M. genovense)* | (Gyimesi et al. 1999) USA; (Keymer et al. 1982) UK; (Portaels et al. 1996) Belgium. | PM of a variety of captive galliformes; PM of a variety of captive galliformes; PM of captive *Coturnix chinensis & Lophortyx californica.* | Inhalation or ingestion of aerosolised bacteria. | Hunting & Butchering  NB A risk for immune-compromised humans. |  |
|  |  | *Salmonella spp* | (Joseph et al. 1988) Malaysia; (Samaha et al. 2012) Egypt. | PM of captive *G. gallus*; S of a variety of wild Galliformes. | Ingestion of raw or undercooked meat.  Faeco-oral transmission. | Consumption  Hunting & Butchering |  |
|  |  | *Yersinia enterocolitica* | (Kato et al. 1985) Japan; (Shayegani et al. 1986) USA. | PM of wild *P. colchicus tohkaidi & Bumbusicola thoracica thoracica*; PM of wild *Meleagris gallopavo & Bonasa umbellus.* | Ingestion of raw or undercooked meat. | Consumption |  |

1. Potential zoonotic parasitic pathogens from wildlife sold in the wild meat trade

Legend: F= faecal sampling, PCR= PCR testing, PM= post mortem examination, S= serology, O= other.

| **WILDLIFE HOST** | | **ZOONOTIC PARASITES** | | | | | |
| --- | --- | --- | --- | --- | --- | --- | --- |
| **Animal class** | **Taxonomic order or family** | **Parasitic pathogen** | **Scientific reference and country (when known)** | **Sampling method and wildlife host** | **Potential transmission route from wildlife to human** | **Potential zoonotic risk from hunting, butchering or consumption** | **Actual zoonotic risk (scientific evidence)** |
| Mammalia | Suidae | *Balantidium coli* | (Nakauchi 1999) Japan; (Solaymani-Mohammadi et al. 2004) Iran. | F from *Sus scrofa*; PM of *S. scrofa.* | Ingestion of faecal contaminated meat.  Faeco-oral transmission of cysts (e.g. contamination of fomites or hands). | Consumption  Hunting &  Butchering |  |
|  |  | *Cryptosporidium spp* | (Atwill et al. 1997) USA; (Castro-Hermida et al. 2011) Spain; (Němejc et al. 2012) Czech Republic. | F from *S. scrofa*; F from *S. scrofa*; PCR of F from *S. scrofa*. | Ingestion of faecal contaminated meat.  Faeco-oral transmission of oocysts (e.g. contamination of fomites or hands). | Consumption  Hunting & Butchering |  |
|  |  | *Giardia spp* | (Atwill et al. 1997) USA; (Castro-Hermida et al. 2011) Spain. | F from *S. scrofa*; F from *S. scrofa*. | Ingestion of faecal contaminated meat.  Faeco-oral transmission of cysts (e.g. contamination of fomites or hands). | Consumption  Hunting & Butchering |  |
|  |  | *Spirometra spp* | (Bengtson and Rogers 2001) USA; (Lee et al. 2013) Korea; (Pavlov 1988) Australia. | PM of *S. scrofa* for all. | Ingestion of plerocercoid larva from raw or undercooked meat. | Consumption | (Tanaka et al. 1997) Japan. |
|  |  | *Taenia spp* | (Fan 1988) Taiwan; (Ito et al. 2003) Asian countries; (Solaymani-Mohammadi et al. 2003) Iran | PM of *S. scrofa* for all. | Ingestion of infective larvae from raw or undercooked meat & viscera. | Consumption | (Fan 1988) & (Fan et al. 1992) Taiwan. |
|  |  | *Toxoplasma gondii* | (Bártová et al. 2006) Czech Republic; (Gauss et al. 2005) Spain; (Gresham et al. 2002) USA; (Kang et al. 2013) South Korea; (Puvanesuaran et al. 2013) Malaysia. | S from PM of *S. scrofa* for all. | Ingestion of tissue cysts from raw or undercooked meat & viscera.  Faeco-oral transmission from carcass preparation. | Consumption  Butchering | (Choi W Y 1997) South Korea. |
|  |  | *Trichinella spp* | (García-Sánchez et al. 2009) Spain; (Gresham et al. 2002) USA; (Kang et al. 2013) South Korea; (Nöckler et al. 2006) Germany; (Pozio et al. 1999) Papua New Guinea. | PM of *S.* scrofa; S from PM of *S. scrofa*; S from PM of *S. scrofa*; PM of *S. scrofa*; S from *S. scrofa.* | Ingestion of tissue cysts from raw or undercooked meat & viscera. | Consumption | (Cui et al. 2011) China; (De Bruyne et al. 2006) & (Ranque et al. 2000) France; (García et al. 2005) Chile; (Gołab and Sadkowska-Todys 2005) Poland; (Greenbloom et al. 1996) Canada; (Jongwutiwes et al. 1998) & (Kusolsuk et al. 2010) Thailand; (Owen et al. 2005) Papua New Guinea; (Rodríguez et al. 2004) Spain. |
|  |  | *Trichuris spp* | (Gipson et al. 1999) USA; (Järvis et al. 2007) Estonia; (Solaymani-Mohammadi et al. 2003) Iran. | PM of *S. scrofa* for all. | Ingestion of faecal contaminated meat.  Faeco-oral transmission of embryonated eggs (e.g. contamination of hands or equipment). | Consumption  Hunting &  Butchering |  |
| Mammalia | Cervidae | *Cryptosporidium spp* | (Castro-Hermida et al. 2011) Spain; (Perz and Le Blancq 2001) & (Rickard et al. 1999) USA. | F from *Capreolus capreolus*; F from *Odocoileus virginianus* for both. | Ingestion of faecal contaminated meat.  Faeco-oral transmission of oocysts. | Consumption  Hunting & Butchering |  |
|  |  | *Giardia spp* | (Castro-Hermida et al. 2011) Spain. | F from *C. capreolus.* | Ingestion of faecal contaminated meat.  Faeco-oral transmission of cysts. | Consumption  Hunting & Butchering |  |
|  |  | *Sarcocystis spp* | (Duncan et al. 2000) USA; (Hernández-Rodríguez et al. 1992) & (López et al. 2003) Spain; (Vinhas 2014) Portugal. | PM of *O. virginianus*; PM of *Dama dama*; PM of *C. capreolus*; PM of *Cervus elaphus & D. dama.* | Ingestion of tissue cysts from raw or undercooked meat (Lee et al. 2014). | Consumption |  |
|  |  | *Toxoplasma gondii* | (Dubey et al. 2008) USA; (Gaffuri et al. 2006) Italy; (Gamarra et al. 2008) Spain; (Vikøren et al. 2004) Norway. | PM of *O. virginianus*; S of *C. capreolus*; S of *C. capreolus*; S of *C. capreolus, C. elaphus, Alces alces & R. tarandus.* | Ingestion of tissue cysts from raw or undercooked meat & viscera.  Faeco-oral transmission from carcass preparation. | Consumption  Butchering | (McDonald et al. 1990) Canada; (Ross et al. 2001) & (Sacks et al. 1983) USA. |
|  |  | *Trichinella spp* | (Bessonov 1981) Russia. | PM of *R. tarandus.* | Ingestion of tissue cysts from raw or undercooked meat & viscera. | Consumption | (Ramasoota 1991) Thailand. |
| Mammalia | Sciuridae | *Sarcocystis spp* | (Latif et al. 2010) Malaysia; (Lindsay et al. 2000) USA. | PM of *Sciurus carolinensis & Xerus* inauris; PM of *Spermophilus tridecemlineatus.* | Ingestion of tissue cysts from raw or undercooked meat ([Fazly et al., 2013](#_ENREF_114)). | Consumption |  |
|  |  | *Toxoplasma gondii* | (Carrasco et al. 2006) Spain; (Dubey et al. 2006) USA; (Jittapalapong et al. 2011) Thailand; (Jokelainen and Nylund 2012) Finland. | PM of imported *Sciurus vulgaris*; PM of *S. carolinensis*;  S of *Callosciurus sp.* & *Menetes berdmorei;* PM of *S. vulgaris.* | Ingestion of tissue cysts from raw or undercooked meat (Fazly et al. 2013).  Faeco-oral transmission from carcass preparation. | Consumption  Butchering | (Alvarado-Esquivel et al. 2008) Mexico. |
| Mammalia | Viverridae | *Gnathostoma spp* | (Colon and Patton 2013) Malaysia. | F from *Viverra tangalunga.* | Ingestion of parasite from raw or undercooked meat & tissues. | Consumption |  |
|  |  | *Sarcocystis spp* | (Fazly et al. 2013) Malaysia. | PM of unspecified civet species. | Ingestion of tissue cysts from raw or undercooked meat (Fazly et al. 2013). | Consumption  (Fazly et al. 2013) |  |
|  |  | *Toxoplasma gondii* | (Oronan et al. 2013) The Philippines. | S of *V. tangalunga & Paradoxurus hermaphrodites.* | Ingestion of tissue cysts from raw or undercooked meat.  Faeco-oral transmission from carcass preparation. | Consumption  Butchering |  |
| Mammalia | Caprinae | *Toxoplasma gondii* | (Kinjo and Minamoto 1987) &  (Sakae and Ishida 2012) Japan. | S of *Capricornis crispis*; S from PM of *C. crispis* | Ingestion of tissue cysts from raw or undercooked meat.  Faeco-oral transmission from carcass preparation. | Consumption  Butchering |  |
| Mammalia | Pteropodidae | Not reported |  |  |  |  |  |
| Mammalia | Hystricidae | Not reported |  |  |  |  |  |
| Mammalia | Ursidae | *Sarcocystis spp* | (Latif et al. 2010) Malaysia. | PM of *Helarctos malayanus.* | Ingestion of tissue cysts from raw or undercooked meat. | Consumption |  |
|  |  | *Toxoplasma gondii* | (Bronson et al. 2014), (Chomel et al. 1995) & (Dubey et al. 2013) USA; (Oksanen et al. 2009) Greenland. | S of *Ursus americanus*; S of *U. americanus*; S of *Ursus arctos & U. americanus*; S of *Ursus maritimus* | Ingestion of tissue cysts from raw or undercooked meat.  Faeco-oral transmission from carcass preparation. | Consumption  Butchering |  |
|  |  | *Trichinella spp* | (Åsbakk et al. 2010) Norway; (Born and Henriksen 1990) Greenland; (Chomel et al. 1998) USA; (Wang et al. 2007) China. | S of *U. maritimus*; PM of *U. maritimus*; S of *U. arctos & U. americanus*; S of unspecified bears. | Ingestion of tissue cysts from raw or undercooked meat. | Consumption | (Ancelle et al. 2005) & (Schellenberg et al. 2003) Canada; (Hall et al. 2012) & (Hill et al. 2005) USA;  (Khamboonruang 1991) Thailand; (Yamaguchi 1991) Japan. |
| Mammalia | Cercopithecidae | *Ancylostoma spp* | (Hussain et al. 2013) India; (Lane et al. 2011) Indonesia; (Lim et al. 2008) Malaysia; (Pourrut et al. 2011) Cameroon. | F from wild *Macaca silenus*; F from *M. fascicularis*; F from captive *M. arctoides, M. radiata & M. nemestrina*; F from 14 different Cercopithecidae species (wild & pets). | Transcutaneous transmission:  penetration of infective larvae through the skin. | Butchering |  |
|  |  | *Balantidium coli* | (Jha et al. 2011) Nepal; (Lim et al. 2008) Malaysia. | F from *M. mulatta*; F from captive *M. arctoides,M. radiata, M. nemestrina & M. fascicularis.* | Ingestion of faecal contaminated meat.  Faeco-oral transmission of cysts. | Consumption  Hunting & Butchering |  |
|  |  | *Cryptosporidium spp* | (Ekanayake et al. 2007) Sri Lanka; (Karim et al. 2014) China; (Lane et al. 2011) Indonesia; (Lim et al. 2008) Malaysia. | F from wild *M. sinica sinica*; F for PCR from wild & captive *M. mulatta & M. fascicularis*; F from wild *M. fascicularis*; F from captive *M. arctoides, M. radiata & M. nemestrina.* | Ingestion of faecal contaminated meat.  Faeco-oral transmission of oocysts. | Consumption  Hunting & Butchering |  |
|  |  | *Entamoeba histolytica* | (Huffman et al. 2013) Sri Lanka; (Jha et al. 2011) Nepal; (Takano et al. 2005) Japan. | F from wild *M. sinica sinica*; F from *M. mulatta*; F from captive *M. fascicularis*. | Ingestion of faecal contaminated meat.  Faeco-oral transmission of cysts (e.g. contamination of fomites or hands). | Consumption  Hunting & Butchering |  |
|  |  | *Giardia spp* | (Karim et al. 2014) China; (Lane et al. 2011) Indonesia. | F for PCR from captive & wild *M. assamensis, M. fuscata, M. mulatta & M. fascicularis*; F from wild *M. fascicularis.* | Ingestion of faecal contaminated meat.  Faeco-oral transmission of cysts. | Consumption  Hunting & Butchering |  |
|  |  | *Oesophagostomum spp* | (Arizono et al. 2012) Japan; (Gotoh et al. 2001) Indonesia. | F from wild *M. fuscata*; F from wild *M. hecki & M. hecki hybrids*. | Ingestion of faecal contaminated meat.  Faeco-oral transmission of filariform larvae (e.g. contamination of hands or equipment). | Consumption  Hunting & Butchering |  |
|  |  | *Sarcocystis spp* | (Tappe et al. 2013) Malaysia; (Tian et al. 2012) & (Yang et al. 2005) China. | PM of wild *M. fascicularis*, PM of *M. fascicularis*; PM of captive *M. fascicularis.* | Ingestion of tissue cysts from raw or undercooked meat. | Consumption |  |
|  |  | *Strongyloides spp* | (Dewit et al. 1991) Sri Lanka; (Gotoh et al. 2001) Indonesia; (Hussain et al. 2013) & (Sharma et al. 2013) India; (Jha et al. 2011) Nepal; (Pourrut et al. 2011) Cameroon; (Wenz-Muecke et al. 2013) Thailand. | F from wild *M. sinica*; F from wild *M. hecki & M. hecki hybrids*; F from wild *M. silenus*; F from wild *M. mulatta*; F from *M. mulatta*; F from 14 different Cercopithecidae species (wild & pets); F from wild *M. fascicularis.* | Direct penetration of infective larvae through the skin (transcutaneous transmission) or oral mucosa (faeco-oral transmission). | Hunting & Butchering |  |
|  |  | *Toxoplasma gondii* | (Ekanayake et al. 2004) Sri Lanka. | S of *M. sinica* | Ingestion of tissue cysts from raw or undercooked meat (Fazly et al. 2013).  Faeco-oral transmission from carcass preparation. | Consumption  Butchering |  |
|  |  | *Trichuris spp* | (Arizono et al. 2012) Japan; (Dewit et al. 1991) & (Huffman et al. 2013) Sri Lanka; (Gotoh et al. 2001) Indonesia; (Jha et al. 2011) Nepal; (Lim et al. 2008) Malaysia; (Pourrut et al. 2011) Cameroon; (Sharma et al. 2013) India; (Wenz-Muecke et al. 2013) Thailand. | F from wild *M. fuscata*; F from wild *M. sinica* for both surveys in Sri Lanka; F from wild *M. hecki & M. hecki hybrids*; F from *M. mulatta*; F from 14 different Cercopithecidae species (wild & pets); F from a variety of captive Cercopithecidae species; F from wild *M. mulatta;* F from wild *M. fascicularis*. | Ingestion of contaminated meat.  Faeco-oral transmission (e.g. contamination of hands or equipment). | Consumption  Hunting & Butchering |  |
| Mammalia | Felidae | *Cryptosporidium spp* | (Lim et al. 2008) Malaysia. | F from captive *Panthera tigris sumatrae, P.tigris. corbetti & P.tigris jacksoni.* | Ingestion of faecal contaminated meat.  Faeco-oral transmission of oocysts (e.g. contamination of fomites or hands). | Consumption  Hunting & Butchering |  |
|  |  | *Toxoplasma gondii* | (Demar et al. 2008) French Guiana; (Goodrich et al. 2012) Russia; (Paul-Murphy et al. 1994) USA; (Thiangtum et al. 2006) Thailand. | PCR of *Panthera onca*; S of *P. tigris altaica*; S of *Puma concolor*; S of captive *P. tigris, Panthera pardua & Neofelis nebulosa.* | Ingestion of faecal contaminated meat.  Faeco-oral transmission through the ingestion of oocysts (e.g. contamination of fomites or hands). | Consumption  Hunting & Butchering | (Carme et al. 2009) French Guiana. |
|  |  | *Trichinella spp* | (Blaga et al. 2009) Romania; (Ribicich et al. 2010) Argentina. | PM of wild *Felis silvestris & Lynx lynx*; PM of wild *P. concolor.* | Ingestion of tissue cysts from raw or undercooked meat. | Consumption |  |
| Mammalia | Manidae | Not reported |  |  |  |  |  |
| Mammalia | Elephantidae | *Cryptosporidium parvum* | (Fayer 2004) review; (Samra et al. 2011) South Africa. | F from *Elaphus maximus & Loxodonta africana*; F from *L. africana* | Ingestion of faecal contaminated meat.  Faeco-oral transmission of oocysts. | Consumption  Hunting & Butchering |  |
| Reptilia | Squamata | *Gnathostoma genus (nematode)* | In snakes: (Cho et al. 2007) China; (Sohn and Lee 1998) Korea. | PM of *Dinodon rufozonatum rufozonatum*; PM of *Agkistrodon brevicaudus*. | Ingestion of larvae from raw or undercooked snake meat. | Consumption | (Akahane et al. 1998) Japan & Thailand; (Seguchi et al. 1995) Japan. |
|  |  | *Pentastomidia (crustacean)* | In snakes: (Ayinmode et al. 2010) Nigeria; (Riley and Self 1981, Kelehear et al. 2014) SE Asia and Australia; (Witchaya Tongtako 2013) Malaysia. | *Pythonidae family*; PM of 7 different snake species; PM of *Python regius*; PM of *Python curtus.* | Ingestion of larvae from raw or undercooked meat.  Direct contact with infected reptile respiratory secretions and tissues.  Faeco-oral transmission of eggs (e.g. contaminated reptile carcasses, hands). | Consumption  Hunting &  Butchering  Butchering | (Latif et al. 2011) & (Prathap et al. 1969) Malaysia; (Yao et al. 2008) & (Ye et al. 2013) China; (Yapo Ette et al. 2003) Ivory Coast. |
|  |  | *Sarcocystis spp* | In snakes: (Daszak and Cunningham 1995) UK; (Lau et al. 2013) Malaysia; (More et al. 2014) Germany.  In lizards: (Bannert 1992) Spain. | PM of *Pituophis melanoleucus sayi*; F from *Naja kaouthia, Braghammerus reticulatus, Ovophis convictus & Ptyas cainata*; F from *Morelia viridis*); O of *Gallotia simonyi.* | Ingestion of contaminated meat.  Faeco-oral transmission through the ingestion of oocysts from this definitive host. | Consumption  Hunting &  Butchering | (Lau et al. 2014) & (Tappe et al. 2013) Malaysia. |
|  |  | *Spirometra spp (cestode)* | (Cho et al. 1982) South Korea; (Sato et al. 2000) Japan. | PM of a variety of snake species; PM of *Elephe quadrivirgata & Rhabdophis tigrinus tigrinus.* | Ingestion of plerocercoid larva from raw or undercooked meat. | Consumption | (Anantaphruti et al. 2011) & (Wiwanitkit 2005) Thailand; (Min 1990) & (Park et al. 2001) South Korea. |
|  |  | *Trichinella spp* | (Pozio et al. 2007) Zimbabwe. | PM of *Varanus varius.* | Ingestion of tissue cysts from raw or undercooked meat. | Consumption | (Khamboonruang 1991) Thailand. |
| Reptilia | Testudines | *Pentastomidia (crustacean)* | (Curran et al. 2014) USA. | PM of softshell turtles *Apalone species.* | Ingestion of larvae from undercooked or raw meat.  Direct contact with infected reptile respiratory secretions and tissues.  Faeco-oral transmission of eggs. | Consumption  Hunting &  Butchering  Butchering |  |
| Reptilia | Crocodylia | *Anisakidae (nematode) e.g. genus Contracaecum* | (Goldberg et al. 1991) Paraguay; (Moravec 2001) Mexico. | PM of *Caiman* yacare; PM of *Crocodylus moreletii.* | Ingestion of larvae from raw or undercooked meat. | Consumption |  |
|  |  | *Pentastomidia* | (Junker et al. 1999) South Africa; (Riley and Huchzermeyer 1995) Republic of Congo. | PM of *Crocodylus niloticus*; PM of *Osteolaemus tetraspis.* | Ingestion of larvae from undercooked or raw meat.  Direct contact with infected reptile respiratory secretions and tissues.  Faeco-oral transmission of eggs. | Consumption  Hunting &  Butchering  Butchering |  |
|  |  | *Trichinella spp* | (Foggin et al. 1997) Zimbabwe; (La Grange et al. 2013) South Africa; (Pozio et al. 2007) Mozambique; (Pozio et al. 2004) Papua New Guinea, | PM of *C. niloticus* for first 3 studies; PM of *Crocodylus porosus.* | Ingestion of tissue cysts from undercooked or raw meat. | Consumption |  |
| Aves | Galliformes | *Cryptosporidium spp* | (Ng et al. 2006) Australia; (Pagès-Manté et al. 2007) Spain; (Randall 1986) UK; (Rohela et al. 2005) Malaysia. | F from captive *Gallus gallus, Rolulus roul roul & Tetrastes bonasia rupestris*; PM of captive *Alectoris rufa;* PM of captive *Gallus sonneratii*; F from captive *Argusianus argus.* | Ingestion of faecal contaminated meat.  Faeco-oral transmission of oocysts. | Consumption  Hunting & Butchering |  |
|  |  | *Sarcocystis spp* | (Latif et al. 2010) Malaysia. | PM of captive *Numida meleagris.* | Ingestion of tissue cysts from raw or undercooked meat. | Consumption |  |

# References for appendix

Addidle M, Grimwade K, Tie S, Rahman H, and Sorenson R (2009) “Pigs might fly”—a case of Erysipelothrix endocarditis. *Journal of the New Zealand Medical Association* 122:78-81.

Akahane H, Sano M, and Kobayashi M (1998) Three cases of human gnathostomiasis caused by Gnathostoma hispidum, with particular reference to the identification of parasitic larvae. *Southeast Asian Journal of Tropical Medicine and Public Health* 29:611-614.

Al Dahouk S, Nöckler K, Tomaso H, Splettstoesser W, Jungersen G, Riber U, Petry T, Hoffmann D, Scholz H, and Hensel A (2005) Seroprevalence of Brucellosis, Tularemia, and Yersiniosis in Wild Boars (Sus scrofa) from North‐Eastern Germany. *Journal of Veterinary Medicine, Series B* 52:444-455.

Alvarado-Esquivel C, Cruz-Magallanes H, Esquivel-Cruz R, Estrada-Martínez S, Rivas-González M, Liesenfeld O, Martínez-García S, Ramírez E, Torres-Castorena A, and Castañeda A (2008) Seroepidemiology of Toxoplasma gondii infection in human adults from three rural communities in Durango State, Mexico. *Journal of Parasitology* 94:811-816.

Anantaphruti MT, Nawa Y, and Vanvanitchai Y (2011) Human sparganosis in Thailand: an overview. *Acta tropica* 118:171-176.

Ancelle T, De Bruyne A, Poisson D, and Dupouy-Camet J (2005) Outbreak of trichinellosis due to consumption of bear meat from Canada, France, September 2005. *Euro Surveillance* 10:2809.

Anderson DC, Geistfeld JG, Maetz HM, Patton CM, and Kaufmann AF (1978) Leptospirosis in zoo workers associated with bears. *The American journal of tropical medicine and hygiene* 27:210-211.

Andrade MCR, Gabeira SCdO, Abreu-Lopes D, Esteves WTC, Vilardo MdCB, Thomé J, Cabello PH, and Lauria-Filgueiras AL (2007) Circulation of Campylobacter spp. in rhesus monkeys (Macaca mulatta) held in captivity: a longitudinal study. *Memorias do Instituto Oswaldo Cruz* 102:53-57.

Angkawanish T, Wajjwalku W, and Sirimalaisuwan A (2010) Mycobacterium tuberculosis infection of domesticated Asian elephants, Thailand. *Emerging infectious diseases* 16:1949-1951.

Arguin PM, Murray-Lillibridge K, Miranda M, Smith JS, Calaor AB, and Rupprecht CE (2002) Serologic evidence of Lyssavirus infections among bats, the Philippines. *Emerging infectious diseases* 8:258-262.

Arizono N, Yamada M, Tegoshi T, and Onishi K (2012) Molecular Identification of Oesophagostomum and Trichuris Eggs Isolated from Wild Japanese Macaques. *The Korean journal of parasitology* 50:253-257.

Asakura H, Makino S-i, Shirahata T, Tsukamoto T, Kurazono H, Ikeda T, and Takeshi K (1998) Detection and Genetical Characterization of Shiga Toxin-Producing Escherichia coli from Wild Deer. *Microbiology and Immunology* 42:815-822.

Åsbakk K, Aars J, Derocher AE, Wiig Ø, Oksanen A, Born EW, Dietz R, Sonne C, Godfroid J, and Kapel CMO (2010) Serosurvey for Trichinella in polar bears (Ursus maritimus) from Svalbard and the Barents Sea. *Veterinary parasitology* 172:256-263.

Aschfalk A, Hundertmark K, Bendiksen H, Arnemo J, and Denzin N (2002) Serosurvey for antibodies against Salmonella species in free-ranging moose (Alces alces) from Norway. *Berliner und Munchener tierarztliche Wochenschrift* 116:417-420.

Aschfalk A, Kemper N, Arnemo J, Veiberg V, Rosef O, and Neubauer H (2008) Prevalence of Yersinia species in healthy free-ranging red deer (Cervus elaphus) in Norway. *Veterinary Record* 163:27-28.

Atwill ER, Sweitzer RA, Pereira M, Gardner I, Van Vuren D, and Boyce WM (1997) Prevalence of and associated risk factors for shedding Cryptosporidium parvum oocysts and Giardia cysts within feral pig populations in California. *Applied and environmental microbiology* 63:3946-3949.

Ayanegui-Alcerreca M, Wilson P, Mackintosh C, Collins-Emerson J, Heuer C, Midwinter A, and Castillo-Alcala F (2007) Leptospirosis in farmed deer in New Zealand: A review. *New Zealand Veterinary Journal* 55:102-108.

Ayinmode A, Adedokun A, Aina A, and Taiwo V (2010) The zoonotic implications of pentastomiasis in the royal python (Python regius). *Ghana medical journal* 44:115-118.

Baer GM, and Smith J (1991) Rabies in nonhematophagous bats. *The natural history of rabies* 2:105-120.

Bagamian KH, Skrypnyk A, Rodina Y, Bezymennyi M, Nevolko O, Skrypnyk V, and Blackburn JK (2014) Serological anthrax surveillance in wild boar (Sus scrofa) in Ukraine. *Vector-Borne and Zoonotic Diseases* 14:618-620.

Bahaman AR, and Ibrahim AL (1988) A review of leptospirosis in Malaysia. *Veterinary research communications* 12:179-189.

Baker M, Lopez L, Cannon M, De Lisle G, and Collins D (2006) Continuing Mycobacterium bovis transmission from animals to humans in New Zealand. *Epidemiology and infection* 134:1068-1073.

Bannert B (1992) Sarcocystis simonyi sp. nov.(Apicomplexa: Sarcocystidae) from the endangered Hierro giant lizardGallotia simonyi (Reptilia: Lacertidae). *Parasitology research* 78:142-145.

Bártová E, Sedlák K, and Literák I (2006) Prevalence of Toxoplasma gondii and Neospora caninum antibodies in wild boars in the Czech Republic. *Veterinary parasitology* 142:150-153.

Baums CG, Verkühlen GJ, Rehm T, Silva LM, Beyerbach M, Pohlmeyer K, and Valentin-Weigand P (2007) Prevalence of Streptococcus suis genotypes in wild boars of Northwestern Germany. *Applied and environmental microbiology* 73:711-717.

Baviskar BS, and Bhandarkar A (2010) Tuberculosis in free ranging Barking Deer (Muntiacus muntjak). *Zoos' Print* 25:18-19.

Bell D, Roberton S, and Hunter PR (2004) Animal origins of SARS coronavirus: possible links with the international trade in small carnivores. *Philosophical Transactions of the Royal Society of London. Series B: Biological Sciences* 359:1107-1114.

Bemis DA, Patton CS, and Ramsay EC (1999) Dermatophilosis in captive tortoises. *Journal of veterinary diagnostic investigation* 11:553-557.

Bender LC, and Hall PB (1996) Leptospira interrogans exposure in free-ranging elk in Washington. *Journal of Wildlife Diseases* 32:121-124.

Bengtson SD, and Rogers F (2001) Prevalence of sparganosis by county of origin in Florida feral swine. *Veterinary parasitology* 97:241-244.

Bensink J, Ekaputra I, and Taliotis C (1991) The isolation of Salmonella from kangaroos and feral pigs processed for human consumption. *Australian Veterinary Journal* 68:106-107.

Bessonov AS (1981) Changes in the epizootic and epidemic situation of trichinellosis in the USSR Review. In: *Trichinellosis: Proceedings of the Fifth International Conference on Trichinellosis*. The Netherlands: Reedbooks, Surrey, England, pp 365-368

Bhatt BD, Zuckerman MJ, Foland JA, Polly SM, and Marwah RK (1989) Disseminated Salmonella arizona infection associated with rattlesnake meat ingestion. *The American journal of gastroenterology* 84:433-435.

Billinis C (2013) Wildlife diseases that pose a risk to small ruminants and their farmers. *Small Ruminant Research* 110:67-70.

Bingham J (1992) Rabies in Zimbabwe. In: *Proceedings of the International Conference on Epidemiology, Control and Prevention of Rabies in Eastern and Southern Africa*. Lusaka, Zambia: Editions Fondation Marcel Mérieux, pp 53-57

Blaga R, Gherman C, Cozma V, Zocevic A, Pozio E, and Boireau P (2009) Trichinella species circulating among wild and domestic animals in Romania. *Veterinary parasitology* 159:218-221.

Born E, and Henriksen S (1990) Prevalence of Trichinella sp. in polar bears (Ursus maritimus) from northeastern Greenland. *Polar Research* 8:313-315.

Bow MR, and Brown JH (1946) Tularemia. A report on 40 cases in Alberta, Canada, 1931-1944. *American Journal of Public Health and the Nations Health* 36:494-500.

Branham LA, Carr MA, Scott CB, and Callaway TR (2005) E. coli O157 and Salmonella spp. in white-tailed deer and livestock. *Current Issues in Intestinal Microbiology* 6:25-29.

Briones V, Téllez S, Goyache J, Ballesteros C, del Pilar Lanzarot M, Domínguez L, and Fernández‐Garayzábal JF (2004) Salmonella diversity associated with wild reptiles and amphibians in Spain. *Environmental microbiology* 6:868-871.

Brody JA, Huntley B, Overfield TM, and Maynard J (1966) Studies of human brucellosis in Alaska. *The Journal of infectious diseases* 116:263-269.

Bronson E, Spiker H, and Driscoll CP (2014) Serosurvey For Selected Pathogens In Free-Ranging American Black Bears (Ursus americanus) In Maryland, USA. *Journal of Wildlife Diseases* 50:829-836.

Brooks JI, Rud EW, Pilon RG, Smith JM, Switzer WM, and Sandstrom PA (2002) Cross-species retroviral transmission from macaques to human beings. *The Lancet* 360:387-388.

Brown R (2005) Leptospirosis in deer slaughter premises. In: *Proceedings of the Deer Branch of the New Zealand Veterinary Association*. New Zealand, p 120

Bruner JA, Griffith R, Greve JH, and Wood RL (1984) Erysipelothrix rhusiopathiae serotype 5 isolated from a white-tailed deer in Iowa. *Journal of Wildlife Diseases* 20:235-236.

Buenviaje G, Ladds P, and Martin Y (1998) Pathology of skin diseases in crocodiles. *Australian Veterinary Journal* 76:357-363.

Burnham B, Atchley D, DeFusco R, Ferris K, Zicarelli J, Lee J, and Angulo F (1998) Prevalence of fecal shedding of Salmonella organisms among captive green iguanas and potential public health implications. *Journal of the American Veterinary Medical Association* 213:48-50.

Butel JS, and Lednicky JA (1999) Cell and Molecular Biology of Simian Virus 40: Implications for Human Infections and Disease. *Journal of the National Cancer Institute* 91:119-134.

Cai W-Q, Sun P-F, and Liu Z-Z (1997) Studies on the Pathogeny and Tissue Pathology of Edwardsielliasis in Trionyx sinensis *Journal of fisheries of China* 4:012.

Capitini CM, Herrero IA, Patel R, Ishitani MB, and Boyce TG (2002) Wound Infection with Neisseria weaveri and a Novel Subspecies of Pasteurella multocida in a Child Who Sustained a Tiger Bite. *Clinical Infectious Diseases* 34:e74-e76.

Cappucci Jr D, Emmons R, and Sampson W (1972) Rabies in an eastern fox squirrel. *Journal of Wildlife Diseases* 8:340-342.

Carbonero A, Paniagua J, Torralbo A, Arenas-Montes A, Borge C, and García-Bocanegra I (2014) Campylobacter infection in wild artiodactyl species from southern Spain: Occurrence, risk factors and antimicrobial susceptibility. *Comparative Immunology, Microbiology and Infectious Diseases* 37:115-121.

Carme B, Demar M, Ajzenberg D, and Dardé ML (2009) Severe acquired toxoplasmosis caused by wild cycle of Toxoplasma gondii, French Guiana. *Emerging infectious diseases* 15:656-658.

Carrasco L, Raya A, Nunez A, Gómez-Laguna J, Hernández S, and Dubey J (2006) Fatal toxoplasmosis and concurrent Calodium hepaticum infection in Korean squirrels (Tanias sibericus). *Veterinary parasitology* 137:180-183.

Carrington M, Choe U, Ubillos S, Stanek D, Campbell M, Wansbrough L, Lee P, Churchwell G, Rosas K, and Zaki S (2012) Fatal case of brucellosis misdiagnosed in early stages of Brucella suis infection in a 46-year-old patient with Marfan syndrome. *Journal of clinical microbiology* 50:2173-2175.

Castro-Hermida JA, García-Presedo I, González-Warleta M, and Mezo M (2011) Prevalence of Cryptosporidium and Giardia in roe deer (Capreolus capreolus) and wild boars (Sus scrofa) in Galicia (NW, Spain). *Veterinary parasitology* 179:216-219.

CDC (1987) Epidemiologic notes and reports B-virus infection in humans—Pensacola, Florida. *Morbility and Mortality Weekly Report (Centers for Disease Control and Prevention)* 36:289–290, 295–296.

CDC (1998) Fatal Cercopithecine herpesvirus 1 (B virus) infection following a mucocutaneous exposure and interim recommendations for worker protection. *Morbility and Mortality Weekly Report (Centers for Disease Control and Prevention)* 47:1073-1076 &1083.

Chan J, Baxter C, and Wenman WM (1989) Brucellosis in an Inuit child, probably related to caribou meat consumption. *Scandinavian journal of infectious diseases* 21:337-338.

Charruau P, Perez-Flores J, Perez-Juarez J, Cedeno-Vazquez J, and Rosas-Carmona R (2012) Oral and cloacal microflora of wild crocodiles Crocodylus acutus and C. moreletii in the Mexican Caribbean. *Diseases of aquatic organisms* 98:27-39.

Chase D, Handsfield H, Allard J, and Taylor J (1980) Tularemia acquired from a bear: Washington. *Morbidity and Mortality Weekly Report* 29:57.

Childs-Sanford SE, Kollias GV, Abou-Madi N, McDonough PL, Garner MM, and Mohammed HO (2009) Yersinia pseudotuberculosis in a closed colony of Egyptian fruit bats (Rousettus aegyptiacus). *Journal of Zoo and Wildlife Medicine* 40:8-14.

Cho S, Song K, and Lee S (1982) Cestode parasites of terrestrial snakes in Korea. *Chung-Ang Journal of Medicine* 7:321-332.

Cho S-H, Kim T-S, Kong Y, Na B-K, and Sohn W-M (2007) Larval Gnathostoma hispidum detected in the red banded odd-tooth snake, Dinodon rufozonatum rufozonatum, from China. *The Korean journal of parasitology* 45:191-198.

Choi W Y NHW, Kwak N H, Huh W, Kim Y R, Kang M W, Cho S Y, Dubey J P (1997) Foodborne outbreaks of human toxoplasmosis. *Journal of Infectious Diseases* 175:1280-1282.

Chomel B, Kasten R, Chappuis G, Soulier M, and Kikuchi Y (1998) Serological survey of selected canine viral pathogens and zoonoses in grizzly bears (Ursus arctos horribilis) and black bears (Ursus americanus) from Alaska. *Revue scientifique et technique (International Office of Epizootics)* 17:756-766.

Chomel BB, Kikuchi Y, Martenson JS, Roelke-Parker ME, Chang C-C, Kasten RW, Foley JE, Laudre J, Murphy K, and Swift PK (2004) Seroprevalence of Bartonella infection in American free-ranging and captive pumas (Felis concolor) and bobcats (Lynx rufus). *Veterinary Research* 35:233-241.

Chomel BB, Zarnke RL, Kasten RW, Kass PH, and Mendes E (1995) Serologic survey of Toxoplasma gondii in grizzly bears (Ursus arctos) and black bears (Ursus americanus), from Alaska, 1988 to 1991. *Journal of Wildlife Diseases* 31:472-479.

Chua K B KCL, Hooi P S, Wee K F, Khong J H, Chua B H, Chan Y P, Lim M E, Lam S K (2002) Isolation of Nipah virus from Malaysian Island flying-foxes. *Microbes and Infection* 4:145-151.

Chua KB, Crameri G, Hyatt A, Yu M, Tompang MR, Rosli J, McEachern J, Crameri S, Kumarasamy V, Eaton BT, and Wang L-F (2007) A previously unknown reovirus of bat origin is associated with an acute respiratory disease in humans. *Proceedings of the National Academy of Sciences* 104:11424-11429.

Clover J, Hofstra T, Kuluris B, Schroeder M, Nelson B, Barnes A, and Botzler R (1989) Serologic evidence of Yersinia pestis infection in small mammals and bears from a temperate rainforest of north coastal California. *Journal of Wildlife Diseases* 25:52-60.

Colon CP, and Patton S (2013) Parasites of civets (Mammalia, Viverridae) in Sabah, Borneo: A caprological survey. *The Malayan Nature Journal* 64:87-94.

Corrente M, Totaro M, Martella V, Campolo M, Lorusso A, Ricci M, and Buonavoglia C (2006) Reptile-associated salmonellosis in man, Italy. *Emerging infectious diseases* 12:358-359.

Cox T, Smythe L, and Leung L-P (2005) Flying foxes as carriers of pathogenic Leptospira species. *Journal of Wildlife Diseases* 41:753-757.

Cubero-Pablo MJ, Plaza M, Pérez L, González M, and León-Vizcaíno L (2000) Seroepidemiology of Chlamydial infections of wild ruminants in Spain. *Journal of Wildlife Diseases* 36:35-47.

Cui B-z, Jin L-x, and Li C-x (2008) Identification of plaque strains isolated from Dege county, Ganz state of Sichuan province by molecular biology assay. *Chinese Journal of Zoonoses* 8:016.

Cui J, Wang Z, and Xu B (2011) The epidemiology of human trichinellosis in China during 2004–2009. *Acta tropica* 118:1-5.

Curran SS, Overstreet RM, Collins DE, and Benz GW (2014) Levisunguis subaequalis ng, n. sp., a tongue worm (Pentastomida: Porocephalida: Sebekidae) infecting softshell turtles, Apalone spp.(Testudines: Trionychidae) in the southeastern United States. *Systematic Parasitology* 87:33-45.

Cvetnic Z, Mitak M, Ocepek M, Lojkic M, Terzic S, Jemersic L, Humski A, Habrun B, Sostaric B, and Brstilo M (2003) Wild boars (Sus scrofa) as reservoirs of Brucella suis biovar 2 in Croatia. *Acta Veterinaria Hungarica* 51:465-473.

Dalsjö A, Nilsson AC, and Ramussen M (2014) Complicated infection caused by Streptococcus suis serotype 14 transmitted from a wild boar. *Journal of Medical Microbiology Case Reports* 1:1-3.

Daszak P, and Cunningham A (1995) A Report of Intestinal Sarcocystosis in the Bullsnake (Pituophis melanoleucus sayi) and a Re-evaluation of Sarcocystis sp. from Snakes of the Genus Pituophis. *Journal of Wildlife Diseases* 31:400-403.

De Bruyne A, Ancelle T, Vallee I, Boireau P, and Dupouy-Camet J (2006) Human trichinellosis acquired from wild boar meat: a continuing parasitic risk in France. *Euro Surveillance* 11:3048.

de Deus N, Peralta B, Pina S, Allepuz A, Mateu E, Vidal D, Ruiz-Fons F, Martín M, Gortázar C, and Segalés J (2008) Epidemiological study of hepatitis E virus infection in European wild boars Sus scrofa in Spain. *Veterinary Microbiology* 129:163-170.

de Fontes-Pereira A, Esponda L, Ley M, Abeledo M, Mafuca J, Agostinho A, and Cañele B (2012) Rabies in an African civet (Civettictis civetta) in Huambo province, Angola. *Revista de Salud Animal* 34:196-199.

Delahay R, De Leeuw A, Claridge M, Harris A, Cheeseman C, Barlow A, Millar M, and Clifton-Hadley R (2001) First report of Mycobacterium bovis in a muntjac deer. *The Veterinary Record* 149:95-96.

Demar M, Ajzenberg D, Serrurier B, Dardé M-L, and Carme B (2008) Atypical Toxoplasma gondii strain from a free-living jaguar (Panthera onca) in French Guiana. *The American journal of tropical medicine and hygiene* 78:195-197.

Desvaux S, Marx N, Ong S, Gaidet N, Hunt M, Manuguerra J-C, Sorn S, Peiris M, Van der Werf S, and Reynes J-M (2009) Highly pathogenic avian influenza virus (H5N1) outbreak in captive wild birds and cats, Cambodia. *Emerging infectious diseases* 15:475-478.

Deutz A, Fuchs K, Schuller W, Nowotny N, Auer H, Aspöck H, Stünzner D, Kerbl U, Klement C, and Köfer J (2002) Seroepidemiological studies of zoonotic infections in hunters in southeastern Austria--prevalences, risk factors, and preventive methods. *Berliner und Munchener tierarztliche Wochenschrift* 116:306-311.

Dewit I, Dittus WP, Vercruysse J, Harris EA, and Gibson DI (1991) Gastro-intestinal helminths in a natural population of Macaca sinica and Presbytis spp. at Polonnaruwa, Sri Lanka. *Primates* 32:391-395.

Di Francesco A, Donati M, Nicoloso S, Orlandi L, Baldelli R, Salvatore D, Sarli G, Cevenini R, and Morandi F (2012) Chlamydiosis: Seroepidemiologic Survey in a Red Deer (Cervus elaphus) Population in Italy. *Journal of Wildlife Diseases* 48:488-491.

Díaz-Sánchez S, Moriones AM, Casas F, and Höfle U (2012) Prevalence of Escherichia coli, Salmonella sp. and Campylobacter sp. in the intestinal flora of farm-reared, restocked and wild red-legged partridges (Alectoris rufa): is restocking using farm-reared birds a risk? *European Journal of Wildlife Research* 58:99-105.

Diesch S, Crawford R, McCulloch W, and Top F (1967) Human leptospirosis acquired from squirrels. *New England Journal of Medicine* 276:838-842.

Dirsmith K, VanDalen K, Fry T, Charles B, VerCauteren K, and Duncan C (2013) Leptospirosis in Fox Squirrels (Sciurus niger) of Larimer County, Colorado, USA. *Journal of Wildlife Diseases* 49:641-645.

Dubey J, Hill D, Zarlenga D, Choudhary S, Ferreira L, Oliveira S, Verma S, Kwok O, Driscoll C, and Spiker H (2013) Isolation and characterization of new genetic types of Toxoplasma gondii and prevalence of Trichinella murrelli from black bear (Ursus americanus). *Veterinary parasitology* 196:24-30.

Dubey J, Hodgin E, and Hamir A (2006) Acute fatal toxoplasmosis in squirrels (Sciurus carolensis) with bradyzoites in visceral tissues. *Journal of Parasitology* 92:658-659.

Dubey JP, Velmurugan GV, Ulrich V, Gill J, Carstensen M, Sundar N, Kwok OCH, Thulliez P, Majumdar D, and Su C (2008) Transplacental toxoplasmosis in naturally-infected white-tailed deer: Isolation and genetic characterisation of Toxoplasma gondii from foetuses of different gestational ages. *International journal for parasitology* 38:1057-1063.

Duncan RB, Fox JH, Lindsay DS, Dubey JP, and Zuccaro ME (2000) Acute Sarcocystosis in a Captive White-tailed Deer in Virginia. *Journal of Wildlife Diseases* 36:357-361.

Durazo A, and Lessenger JE (2006) Mammal Bites. In: *Agricultural Medicine: A Practical Guide,* Lessenger JE (editor), New York, USA: Springer pp 430-439

Eales KM, Norton RE, and Ketheesan N (2010) Brucellosis in northern Australia. *The American journal of tropical medicine and hygiene* 83:876-878.

Ebani VV, Cerri D, Poli A, and Andreani E (2003) Prevalence of Leptospira and Brucella antibodies in wild boars (Sus scrofa) in Tuscany, Italy. *Journal of Wildlife Diseases* 39:718-722.

Eggert M, Stuber E, Heurich M, Fredriksson-Ahomaa M, Burgos Y, Beutin L, and Martlbauer E (2013) Detection and characterization of Shiga toxin-producing Escherichia coli in faeces and lymphatic tissue of free-ranging deer. *Epidemiology and infection* 141:251-259.

Ekanayake D, Rajapakse RPV, Dubey J, and Dittus WPJ (2004) Seroprevalence of Toxoplasma gondii in wild toque macaques (Macaca sinica) at Polonnaruwa, Sri Lanka. *Journal of Parasitology* 90:870-871.

Ekanayake DK, Welch DM, Kieft R, Hajduk S, and Dittus WP (2007) Transmission dynamics of Cryptosporidium infection in a natural population of non-human primates at Polonnaruwa, Sri Lanka. *The American journal of tropical medicine and hygiene* 77:818-822.

Engel GA, Jones-Engel L, Schillaci MA, Suaryana KG, Putra A, Fuentes A, and Henkel R (2002) Human exposure to herpesvirus B-seropositive macaques, Bali, Indonesia. *Emerging infectious diseases* 8:789-795.

Engels EA, Switzer WM, Heneine W, and Viscidi RP (2004) Serologic evidence for exposure to simian virus 40 in North American zoo workers. *Journal of Infectious Diseases* 190:2065-2069.

Epstein JH, Luby S, and Pulliam JR (2006) Nipah virus: impact, origins, and causes of emergence. *Current infectious disease reports* 8:59-65.

Epstein JH, Prakash V, Smith CS, Daszak P, McLaughlin AB, Meehan G, Field HE, and Cunningham AA (2008) Henipavirus infection in fruit bats (Pteropus giganteus), India. *Emerging infectious diseases* 14:1309-1311.

Eskens U, and Zschock M (1991) Rotlaufinfektion beim Reh-ein Fallbericht. *Tierärztliche Praxis* 19:52-53.

Esmaeili S, Gooya MM, Shirzadi MR, Esfandiari B, Amiri FB, Behzadi MY, Banafshi O, and Mostafavi E (2014) Seroepidemiological survey of tularemia among different groups in western Iran. *International journal of infectious diseases* 18:27-31.

Fan PC (1988) Taiwan Taenia and Taeniasis. *Parasitology Today* 4:86-88.

Fan PC, Chung WC, Soh CT, and Kosman ML (1992) Eating habits of East Asian people and transmission of taeniasis. *Acta tropica* 50:305-315.

Fanning A, and Edwards S (1991) Mycobacterium bovis infection in human beings in contact with elk (Cervus elaphus) in Alberta, Canada. *The Lancet* 338:1253-1255.

Fasanella A, Palazzo L, Petrella A, Quaranta V, Romanelli B, and Garofolo G (2007) Anthrax in red deer (Cervus elaphus), Italy. *Emerging infectious diseases* 13:1118-1119.

Favoretto SR, de Mattos CC, Morais NB, Araújo FA, and de Mattos CA (2001) Rabies in marmosets (Callithrix jacchus), Ceará, Brazil. *Emerging infectious diseases* 7:1062-1065.

Fayer R (2004) Cryptosporidium: a water-borne zoonotic parasite. *Veterinary parasitology* 126:37-56.

Fazly Z, Nurulaini R, Shafarin M, Fariza N, Zawida Z, Muhamad H, Adnan M, Premaalatha B, Erwanas A, and Zaini C (2013) Zoonotic parasites from exotic meat in Malaysia. *Tropical biomedicine* 30:535-542.

Ferreira Junior R, S, Rui S, Siqueira AK, Campagner MV, Salerno T, Soares T, Lucheis SB, Paes AC, and Barraviera B (2009) Comparison of wildlife and captivity rattlesnakes (Crotalus durissus terrificus) microbiota. *Pesquisa Veterinária Brasileira* 29:999-1003.

Ferroglio E, Tolari F, Bollo E, and Bassano B (1998) Isolation of Brucella melitensis from Alpine Ibex. *Journal of Wildlife Diseases* 34:400-402.

Ferronato BO, Marques TS, Souza FL, Verdade LM, and Matushima ER (2009) Oral bacterial microbiota and traumatic injuries of free-ranging Phrynops geoffroanus (Testudines, Chelidae) in southeastern Brazil. *Phyllomedusa: Journal of Herpetology* 8:19-25.

Foggin C, Vassilev G, and Widdowson M (1997) Infection with Trichinella in farmed crocodiles (Crocodylus niloticus) in Zimbabwe. In: *Abstract book on the 16th International Conference of The World Association for the Advancement of Veterinary Parasitology*. Sun City, South Africa: The World Association for the Advancement of Veterinary Parasitology, p 15

Forbes LB (1991) Isolates of Brucella suis biovar 4 from animals and humans in Canada, 1982-1990. *The Canadian Veterinary Journal* 32:686-688.

Fredriksson-Ahomaa M, Wacheck S, Koenig M, Stolle A, and Stephan R (2009) Prevalence of pathogenic Yersinia enterocolitica and Yersinia pseudotuberculosis in wild boars in Switzerland. *International journal of food microbiology* 135:199-202.

Friedman CR, Torigian C, Shillam PJ, Hoffman RE, Heltze D, Beebe JL, Malcolm G, DeWitt WE, Hutwagner L, and Griffin PM (1998) An outbreak of salmonellosis among children attending a reptile exhibit at a zoo. *The Journal of pediatrics* 132:802-807.

Fukushima H, and Gomyoda M (1991) Intestinal carriage of Yersinia pseudotuberculosis by wild birds and mammals in Japan. *Applied and environmental microbiology* 57:1152-1155.

Fukushima H, Okuno J, Fujiwara Y, Hosoda T, Kurazono T, Ohtsuka K, Yanagawa K, and Yamaguchi M (2008) An outbreak of Salmonella food poisoning at a snapping turtle restaurant. *Journal of the Japanese Association for Infectious Diseases* 61:328.

Gaffuri A, Giacometti M, Tranquillo VM, Magnino S, Cordioli P, and Lanfranchi P (2006) Serosurvey of roe deer, chamois and domestic sheep in the central Italian Alps. *Journal of Wildlife Diseases* 42:685-690.

Gamarra J, Cabezón O, Pabón M, Arnal M, Luco D, Dubey J, Gortázar C, and Almeria S (2008) Prevalence of antibodies against Toxoplasma gondii in roe deer from Spain. *Veterinary parasitology* 153:152-156.

García E, Mora L, Torres P, Jercic MI, and Mercado R (2005) First record of human trichinosis in Chile associated with consumption of wild boar (Sus scrofa). *Memorias do Instituto Oswaldo Cruz* 100:17-18.

García-Sánchez A, Sánchez S, Rubio R, Pereira G, Alonso J, Hermoso de Mendoza J, and Rey J (2007) Presence of Shiga toxin-producing E. coli O157: H7 in a survey of wild artiodactyls. *Veterinary Microbiology* 121:373-377.

García-Sánchez R, Nogal-Ruiz J, Manzano-Lorenzo R, Díaz J, Lopez GP, Ruano F, Casas AR, Bascon CC, Bolás-Fernández F, and Martínez-Fernández A (2009) Trichinellosis survey in the wild boar from the Toledo mountains in south-western Spain (2007–2008): molecular characterization of Trichinella isolates by ISSR-PCR. *Journal of helminthology* 83:117-120.

Garin-Bastuji B, Oudar J, Richard Y, and Gastellu J (1990) Isolation of Brucella melitensis Biovar 3 from a Chamois (Rupicapra rupicapra) in the Southern French Alps. *Journal of Wildlife Diseases* 26:116-118.

Garin-Bastuji B, Vaillant V, Albert D, Tourrand B, Danjean M, Lagier A, Rispal P, Benquet B, Maurin M, and De Valk H (2006) Is brucellosis due the biovar 2 of Brucella suis an emerging zoonosis in France? Two case reports in wild boar and hare hunters. In: *Proceedings of the International Society of Chemotherapy Disease Management Meeting, 1st International Meeting on Treatment of Human Brucellosis* Loannina, Greece: International Society of Chemotherapy Disease Management, pp 7-10

Gauss C, Dubey J, Vidal D, Ruiz F, Vicente J, Marco I, Lavin S, Gortazar C, and Almeria S (2005) Seroprevalence of Toxoplasma gondii in wild pigs (Sus scrofa) from Spain. *Veterinary parasitology* 131:151-156.

Gilbert MJ, Kik M, Timmerman AJ, Severs TT, Kusters JG, Duim B, and Wagenaar JA (2014) Occurrence, diversity, and host association of intestinal Campylobacter, Arcobacter, and Helicobacter in reptiles. *PLoS ONE* 9:e101599.

Gill C (2007) Microbiological conditions of meats from large game animals and birds. *Meat science* 77:149-160.

Gipson PS, Veatch JK, Matlack RS, and Jones DP (1999) Health Status of a Recently Discovered Population of Feral Swine in Kansas. *Journal of Wildlife Diseases* 35:624-627.

Giurgiutiu D, Banis C, Hunt E, Mincer J, Nicolardi C, Weltman A, Stanek D, Matthews S, Siegenthaler C, and Blackmore C (2009) Brucella suis infection associated with feral swine hunting-Three States, 2007-2008. *Morbidity and Mortality Weekly Report* 58:618-621.

Godfroid J, Michel P, Uytterhaegen L, Smedt Cd, Rasseneur F, Boelaert F, Saegerman C, and Patigny X (1994) Brucella suis biotype 2 infection of wild boars (Sus scrofa) in Belgium. *Annales de Medecine Veterinaire (Belgium)* 138.

Gołab E, and Sadkowska-Todys M (2005) Epidemiology of human trichinellosis in Poland--currently and in the past. *Wiadomosci parazytologiczne* 52:181-187.

Goldberg SR, Bursey CR, and Aquino-Shuster A (1991) Gastric nematodes of the Paraguayan caiman, Caiman yacare (Alligatoridae). *The Journal of parasitology* 77:1009-1011.

Goodrich JM, Quigley KS, Lewis JCM, Astafiev AA, Slabi EV, Miquelle DG, Smirnov EN, Kerley LL, Armstrong DL, Quigley HB, and Hornocker MG (2012) Serosurvey of Free-ranging Amur Tigers in the Russian Far East. *Journal of Wildlife Diseases* 48:186-189.

Goodwin B, Jerome C, and Bullock B (1988) Unusual lesion morphology and skin test reaction for Mycobacterium avium complex in macaques. *Laboratory animal science* 38:20-24.

Gotoh S, Takenaka O, Watanabe K, Hamada Y, Kawamoto Y, Watanabe T, Suryobroto B, and Sajuthi D (2001) Hematological values and parasite fauna in free-rangingMacaca hecki and theM. hecki/M. tonkeana hybrid group of Sulawesi Island, Indonesia. *Primates* 42:27-34.

Gozzi AC, Guichón ML, Benitez VV, Romero GN, Auteri C, and Brihuega B (2013) First isolation of Leptospira interrogans from the arboreal squirrel Callosciurus erythraeus introduced in Argentina. *Wildlife Biology* 19:483-489.

Greenbloom SL, Martin-Smith P, Isaacs S, Marshall B, Kittle DC, Kain KC, and Keystone JS (1996) Outbreak of trichinosis in Ontario secondary to the ingestion of wild boar meat. *Canadian journal of public health* 88:52-56.

Greenwood A, and Sanchez S (2002) Serological evidence of murine pathogens in wild grey squirrels (Sciurus carolinensis) in North Wales. *The Veterinary Record* 150:543-546.

Gresham CS, Gresham CA, Duffy MJ, Faulkner CT, and Patton S (2002) Increased prevalence of Brucella suis and pseudorabies virus antibodies in adults of an isolated feral swine population in coastal South Carolina. *Journal of Wildlife Diseases* 38:653-656.

Guan Y, Zheng B, He Y, Liu X, Zhuang Z, Cheung C, Luo S, Li P, Zhang L, and Guan Y (2003) Isolation and characterization of viruses related to the SARS coronavirus from animals in southern China. *Science* 302:276-278.

Gyimesi ZS, Stalis IH, Miller JM, and Thoen CO (1999) Detection of Mycobacterium avium Subspecies avium in Formalin-Fixed, Paraffin-Embedded Tissues of Captive Exotic Birds Using Polymerase Chain Reaction. *Journal of Zoo and Wildlife Medicine* 30:348-353.

Halaby T, Hoitsma E, Hupperts R, Spanjaard L, Luirink M, and Jacobs J (2000) Streptococcus suis meningitis, a poacher's risk. *European Journal of Clinical Microbiology and Infectious Diseases* 19:943-945.

Hall RL, Lindsay A, Hammond C, Montgomery SP, Wilkins PP, da Silva AJ, McAuliffe I, de Almeida M, Bishop H, and Mathison B (2012) Outbreak of human trichinellosis in Northern California caused by Trichinella murrelli. *The American journal of tropical medicine and hygiene* 87:297-302.

Hanna JN, Carney IK, Smith GA, Tannenberg A, Deverill JE, Botha JA, Serafin IL, Harrower BJ, Fitzpatrick PF, and Searle JW (2000) Australian bat lyssavirus infection: a second human case, with a long incubation period. *The Medical Journal of Australia* 172:597-599.

Harris JR, Bergmire-Sweat D, Schlegel JH, Winpisinger KA, Klos RF, Perry C, Tauxe RV, and Sotir MJ (2009) Multistate outbreak of Salmonella infections associated with small turtle exposure, 2007–2008. *Pediatrics* 124:1388-1394.

Hasselschwert DL, and Ostrowski SR (1999) An Atypical Case of Mycobacterium bovis in a Cynomolgus Macaque (Macaca fascicularis) Imported From the Philippines. *Journal of the American Association for Laboratory Animal Science* 38:36-38.

Hayashidani H, Kanzaki N, Kaneko Y, Okatani AT, Taniguchi T, Kaneko K-i, and Ogawa M (2002) Occurrence of Yersiniosis and Listeriosis in wild boars in Japan. *Journal of Wildlife Diseases* 38:202-205.

Hemmer CJ, Littmann M, Löbermann M, Meyer H, Petschaelis A, and Reisinger EC (2010) Human cowpox virus infection acquired from a circus elephant in Germany. *International journal of infectious diseases* 14, Supplement 3:e338-e340.

Henderson TG (1984) The isolation of Yersinia sp. from feral and farmed deer faeces. *New Zealand Veterinary Journal* 32:88-90.

Hennig GE, Goebel HD, Fabis JJ, and Khan MI (2002) Diagnosis by Polymerase Chain Reaction of Erysipelas Septicemia in a Flock of Ring-Necked Pheasants. *Avian Diseases* 46:509-514.

Hernández-Rodríguez S, Acosta I, and Navarrete I (1992) Sarcocystis jorrini sp. nov. from the fallow deer Cervus dama. *Parasitology research* 78:557-562.

Hidalgo-Vila J, Díaz-Paniagua C, de Frutos-Escobar C, Jiménez-Martínez C, and Pérez-Santigosa N (2007) Salmonella in free living terrestrial and aquatic turtles. *Veterinary Microbiology* 119:311-315.

Higgins R, Lagacé A, Messier S, and Julien L (1997) Isolation of Streptococcus suis from a young wild boar. *The Canadian Veterinary Journal* 38:114.

Hill D, Gamble H, Zarlenga D, Coss C, and Finnigan J (2005) Trichinella nativa in a black bear from Plymouth, New Hampshire. *Veterinary parasitology* 132:143-146.

Holmes GP, Hilliard JK, Klontz KC, Rupert AH, Schindler CM, Parrish E, Griffin DG, Ward GS, Bernstein ND, and Bean TW (1990) B virus (Herpesvirus simiae) infection in humans: epidemiologic investigation of a cluster. *Annals of internal medicine* 112:833-839.

Hopkins B, Skeeles J, Houghten G, Slagle D, and Gardner K (1990) A survey of infectious diseases in wild turkeys (Meleagridis gallopavo silvestris) from Arkansas. *Journal of Wildlife Diseases* 26:468-472.

Horner G, Robinson A, Hunter R, Cox B, and Smith R (1987) Parapoxvirus infections in New Zealand farmed red deer (Cervus elaphus). *New Zealand Veterinary Journal* 35:41-45.

Hotta A, Tanabayashi K, Yamamoto Y, Fujita O, Uda A, Mizoguchi T, and Yamada A (2012) Seroprevalence of tularemia in wild bears and hares in Japan. *Zoonoses and Public Health* 59:89-95.

Hotzel H, Berndt A, Melzer F, and Sachse K (2004) Occurrence of Chlamydiaceae spp. in a wild boar (Sus scrofa L.) population in Thuringia (Germany). *Veterinary Microbiology* 103:121-126.

Hsu VP, Hossain MJ, Parashar UD, Ali MM, Ksiazek TG, Kuzmin I, Niezgoda M, Rupprecht C, Bresee J, and Breiman RF (2004) Nipah virus encephalitis reemergence, Bangladesh. *Emerging infectious diseases* 10:2082-2087.

Huang F, Wang H, Jing S, and Zeng W (2012) Simian foamy virus prevalence in Macaca mulatta and zookeepers. *AIDS research and human retroviruses* 28:591-593.

Hubálek Z, Treml F, Juricova Z, Hunady M, Halouzka J, Janik V, and Bill D (2002) Serological survey of the wild boar (Sus scrofa) for tularaemia and brucellosis in South Moravia, Czech Republic. *Veterinari Medicina Praha* 47:60-66.

Huchzermeyer K (1991) Treatment and control of an outbreak of salmonellosis in hatchling Nile crocodiles (Crocodylus niloticus). *Journal of the South African Veterinary Association* 62:23-25.

Huff JL, and Barry PA (2003) B-virus (Cercopithecine herpesvirus 1) infection in humans and macaques: potential for zoonotic disease. *Emerging infectious diseases* 9:246-250.

Huffman M, Nahallage C, Hasegawa H, Ekanayake S, De Silva L, and Athauda I (2013) Preliminary survey of the distribution of four potentially zoonotic parasite species among primates in Sri Lanka. *Journal of the National Science Foundation of Sri Lanka* 41:319-326.

Hussain S, Ram MS, Kumar A, Shivaji S, and Umapathy G (2013) Human presence increases parasitic load in endangered lion-tailed macaques (Macaca silenus) in its fragmented rainforest habitats in southern India. *PLoS ONE* 8:e63685-e63685.

Ichhpujani R, Rajagopal V, Bhattacharya D, Rana U, Mittal V, Rai A, Ravishankar A, Pasha S, Sokhey J, and Biswas S (2004) An outbreak of human anthrax in Mysore (India). *The Journal of communicable diseases* 36:199-204.

Inoshima Y, Shimizu S, Minamoto N, Hirai K, and Sentsui H (1999) Use of protein AG in an enzyme-linked immunosorbent assay for screening for antibodies against parapoxvirus in wild animals in Japan. *Clinical and diagnostic laboratory immunology* 6:388-391.

Inoshima Y, Yamamoto Y, Takahashi T, Shino M, Katsumi A, Shimizu S, and Sentsui H (2001) Serological survey of parapoxvirus infection in wild ruminants in Japan in 1996–9. *Epidemiology and infection* 126:153-156.

Irwin MJ, Massey PD, Walker B, and Durrheim DN (2010) Feral pig hunting: a risk factor for human brucellosis in north-west NSW? *New South Wales public health bulletin* 20:192-194.

Isotalo P, Edgar D, and Toye B (2000) Polymicrobial tenosynovitis with Pasteurella multocida and other gram negative bacilli after a Siberian tiger bite. *Journal of clinical pathology* 53:871-872.

Ito A, Nakao M, and Wandra T (2003) Human taeniasis and cysticercosis in Asia. *The Lancet* 362:1918-1920.

Jansen A, Luge E, Guerra B, Wittschen P, Gruber AD, Loddenkemper C, Schneider T, Lierz M, Ehlert D, and Appel B (2007) Leptospirosis in urban wild boars, Berlin, Germany. *Emerging infectious diseases* 13:739-742.

Janssen DL, Anderson MP, Abildgaard S, and Silverman S (1989) Tuberculosis in Newly Imported Tibetan Macaques (Macaca thibetans). *Journal of Zoo and Wildlife Medicine* 20:315-321.

Järvis T, Kapel C, Moks E, Talvik H, and Mägi E (2007) Helminths of wild boar in the isolated population close to the northern border of its habitat area. *Veterinary parasitology* 150:366-369.

Jay MT, Cooley M, Carychao D, Wiscomb GW, Sweitzer RA, Crawford-Miksza L, Farrar JA, Lau DK, O’Connell J, and Millington A (2007) Escherichia coli O157: H7 in feral swine near spinach fields and cattle, central California coast. *Emerging infectious diseases* 13:1908-1911.

Jay MT, and Wiscomb GW (2008) Food safety risks and mitigation strategies for feral swine (Sus scrofa) near agriculture fields. In: *Proceedings of the 23rd Vertebrate Pest Conference* San Diego, California, USA: University of California, Davis, pp 21-25

Jha A, Chalise MK, Shrestha RM, and Karki K (2011) Intestinal Parasitic Investigation in Temple Rhesus Monkeys of Kathmandu. *The Initiation* 4:1-7.

Jittapalapong S, Sarataphan N, Maruyama S, Hugot J-P, Morand S, and Herbreteau V (2011) Toxoplasmosis in rodents: ecological survey and first evidences in Thailand. *Vector-Borne and Zoonotic Diseases* 11:231-237.

Johnston MA, Porter DE, Scott GI, Rhodes WE, and Webster LF (2010) Isolation of faecal coliform bacteria from the American alligator (Alligator mississippiensis). *Journal of Applied Microbiology* 108:965-973.

Jokelainen P, and Nylund M (2012) Acute Fatal Toxoplasmosis in Three Eurasian Red Squirrels (Sciurus vulgaris) Caused by Genotype II of Toxoplasma gondii. *Journal of Wildlife Diseases* 48:454-457.

Jones-Engel L, Engel GA, Heidrich J, Chalise M, Poudel N, Viscidi R, Barry PA, Allan JS, Grant R, and Kyes R (2006) Temple monkeys and health implications of commensalism, Kathmandu, Nepal. *Emerging infectious diseases* 12:900-906.

Jones-Engel L, Engel GA, Schillaci MA, Rompis A, Putra A, Suaryana KG, Fuentes A, Beer B, Hicks S, and White R (2005) Primate-to-human retroviral transmission in Asia. *Emerging infectious diseases* 11(7):1028-1035.

Jones-Engel L, May CC, Engel GA, Steinkraus KA, Schillaci MA, Fuentes A, Rompis A, Chalise MK, Aggimarangsee N, and Feeroz MM (2008) Diverse contexts of zoonotic transmission of simian foamy viruses in Asia. *Emerging infectious diseases* 14:1200-1208.

Jones-Engel L, Steinkraus KA, Murray SM, Engel GA, Grant R, Aggimarangsee N, Lee BP-H, May C, Schillaci MA, and Somgird C (2007) Sensitive assays for simian foamy viruses reveal a high prevalence of infection in commensal, free-ranging Asian monkeys. *Journal of virology* 81:7330-7337.

Jongwutiwes S, Chantachum N, Kraivichian P, Siriyasatien P, Putaporntip C, Tamburrini A, La Rosa G, Sreesunpasirikul C, Yingyourd P, and Pozio E (1998) First outbreak of human trichinellosis caused by Trichinella pseudospiralis. *Clinical Infectious Diseases* 26:111-115.

Joseph P, Sivanandan S, and Yee HT (1988) Animal salmonella surveillance in Peninsular Malaysia, 1981–1985. *Epidemiology and infection* 100:351-359.

Junker K, Boomker JDF, and Bolton LA (1999) Pentastomid infections in Nile crocodiles (Crocodylus niloticus) in the Kruger National Park, South Africa, with a description of the males of Alofia simpsoni. *The Onderstepoort journal of veterinary research* 66:65-71.

Kaba M, Davoust B, Marié J-L, and Colson P (2010) Detection of hepatitis E virus in wild boar Sus scrofa livers. *The Veterinary Journal* 186:259-261.

Kaci S, Nöckler K, and Johne R (2008) Detection of hepatitis E virus in archived German wild boar serum samples. *Veterinary Microbiology* 128:380-385.

Kaden V, Lange E, Starick E, Bruer W, Krakowski W, and Klopries M (2008) Epidemiological survey of swine influenza A virus in selected wild boar populations in Germany. *Veterinary Microbiology* 131:123-132.

Kadir HA, Nurdiyana S, Bahaman AR, Kumar Sharma RS, Sugnaseelan S, Che’Amat A, Razak A, and Ariff MF (2012) Serological Prevalence Of Leptospira Infection In Malayan Porcupines (Hystrix Brachyura) In Captivity. In: *Proceedings of the 7th Seminar in Veterinary Sciences*. Universiti Putra Malaysia, Selangor, Malaysia, pp 66-69

Kalashnikova V, Dzhikidze E, Stasilevich Z, and Chikobava M (2002) Detection of Campylobacter jejuni in healthy monkeys and monkeys with enteric infections by PCR. *Bulletin of experimental biology and medicine* 134:299-300.

Kanai Y, Hayashidani H, Kaneko K-I, Ogawa M, Takahashi T, and Nakamurat M (1997) Occurrence of zoonotic bacteria in retail game meat in Japan with special reference to Erysipelothrix. *Journal of Food Protection* 60:328-331.

Kang SW, Doan HTT, Noh JH, Choe SE, Yoo MS, Kim YH, Reddy KE, Nguyen TTD, Van Quyen D, Nguyen LTK, Kweon CH, and Jung SC (2013) Seroprevalence of Toxoplasma gondii and Trichinella spiralis infections in wild boars (Sus scrofa) in Korea. *Parasitology International* 62:583-585.

Karim MR, Zhang S, Jian F, Li J, Zhou C, Zhang L, Sun M, Yang G, Zou F, Dong H, Li J, Rume FI, Qi M, Wang R, Ning C, and Xiao L (2014) Multilocus typing of Cryptosporidium spp. and Giardia duodenalis from non-human primates in China. *International journal for parasitology* 44:1039-1047.

Kato Y, Ito K, Kubokura Y, Maruyama T, Kaneko K, and Ogawa M (1985) Occurrence of Yersinia enterocolitica in wild-living birds and Japanese serows. *Applied and environmental microbiology* 49:198-200.

Keawcharoen J, Oraveerakul K, Kuiken T, Fouchier R, Amonsin A, Payungporn S, Noppornpanth S, Wattanodorn S, Theambooniers A, and Tantilertcharoen R (2004) Avian influenza H5N1 in tigers and leopards. *Emerging infectious diseases* 10:2189-2191.

Keene WE, Sazie E, Kok J, Rice DH, Hancock DD, Balan VK, Zhao T, and Doyle MP (1997) An outbreak of Escherichia coli 0157: H7 infections traced to jerky made from deer meat. *The Journal of the American Medical Association* 277:1229-1231.

Kelehear C, Spratt DM, O’Meally D, and Shine R (2014) Pentastomids of wild snakes in the Australian tropics. *International Journal for Parasitology: Parasites and Wildlife* 3:20-31.

Kelly J, Hopkin R, and Rimsza ME (1995) Rattlesnake meat ingestion and Salmonella arizona infection in children: case report and review of the literature. *The Pediatric infectious disease journal* 14:320-321.

Kennedy FM, Astbury J, Needham J, and Cheasty T (1993) Shigellosis due to occupational contact with non-human primates. *Epidemiology and infection* 110:247-251.

Keymer I, Jones D, Pugsley S, and Wadsworth P (1982) A survey of tuberculosis in birds in the Regent's Park Gardens of the Zoological Society of London. *Avian Pathology* 11:563-569.

Khamboonruang C (1991) The present status of trichinellosis in Thailand. *Southeast Asian Journal of Tropical Medicine Public Health* 22 (Supplement):312-325.

Khoshbakht R, Tabatabaei M, Aski HS, and Shayegh H (2014) Distribution of Salmonella, Arcobacter, and thermophilic Campylobacter spp. among Persian fallow deer (Dama mesopotamica) population in Dasht-e-Arzhan Wildlife refuge, southern Iran. *Comparative Clinical Pathology* 24:777-781.

Kik M, Luten H, Wibbelt G, Kretschmar P, Hofer H, and Seet S (2009) Cowpoxvirus infection in Asian elephants (Elephas maximus) in a zoo. In: *Proceedings of the International Conference on Diseases of Zoo and Wild Animals*. Beekse Bergen, The Netherlands, : Leibniz Institute for Zoo and Wildlife Research, pp 7-10

Kim S-H, Choi H, Yoon J, Woo C, Chung H-M, Kim J-T, and Shin J-H (2014) Pathogens in Water deer (Hydropotes Inermis) in South Korea, 2010-2012. *Journal of Wildlife Diseases* 50:478-483.

Kinjo T, and Minamoto N (1987) Serological survey for selected microbial pathogens in Japanese serow (Capricornis crispus) in Gifu Prefecture, Japan. In: *The Biology and Management of Capricornis and Related Mountain Antelopes,* Soma H (editor), The Netherlands: Springer pp 299-311

Klein J, and Tryland M (2005) Characterisation of parapoxviruses isolated from Norwegian semi-domesticated reindeer (Rangifer tarandus tarandus). *Virology journal* 2:79.

Koeboelkuti LB, Czirjak GA, Tenk M, Szakacs A, Kelemen A, and Spinu M (2013) Edwardsiella tarda Associated Subcutaneous Abscesses in A Captive Grass Snake (Natrix natrix, Squamata: Colubridae). *Kafkas Üniversitesi Veteriner Fakültesi Dergisi* 19:1061-1063.

Kuhl JT, Huerter CJ, and Hashish H (2003) A case of human orf contracted from a deer. *Cutis* 71:288-290.

Kuhn K, Torpdahl M, Frank C, Sigsgaard K, and Ethelberg S (2011) An outbreak of Salmonella Typhimurium traced back to salami, Denmark, April to June 2010. *Euro Surveillance* 16:1-4.

Kumari PL, Mohanan KR, Kailas L, and Chacko KP (2014) A Case of Rabies after Squirrel Bite. *The Indian Journal of Pediatrics* 81:198-198.

Kuroki T, Ishihara T, Furukawa I, Okatani AT, and Kato Y (2013) Prevalence of Salmonella in Wild Snakes in Japan. *Japanese journal of infectious diseases* 66:295-298.

Kurth A, Wibbelt G, Gerber H-P, Petschaelis A, Pauli G, and Nitsche A (2008) Rat-to-elephant-to-human transmission of cowpox virus. *Emerging infectious diseases* 14:670-671.

Kusolsuk T, Kamonrattanakun S, Wesanonthawech A, Dekumyoy P, Thaenkham U, Yoonuan T, Nuamtanong S, Sa-nguankiat S, Pubampen S, and Maipanich W (2010) The second outbreak of trichinellosis caused by Trichinella papuae in Thailand. *Transactions of the Royal Society of Tropical Medicine and Hygiene* 104:433-437.

La Grange L, Govender D, and Mukaratirwa S (2013) The occurrence of Trichinella zimbabwensis in naturally infected wild crocodiles (Crocodylus niloticus) from the Kruger National Park, South Africa. *Journal of helminthology* 87:91-96.

Labelle P, Mikaelian I, Martineau D, Beaudin S, Blanchette N, Lafond R, and St-Onge S (2000) Seroprevalence of leptospirosis in lynx and bobcats from Quebec. *The Canadian Veterinary Journal* 41:319.

Lane KE, Holley C, Hollocher H, and Fuentes A (2011) The anthropogenic environment lessens the intensity and prevalence of gastrointestinal parasites in Balinese long-tailed macaques (Macaca fascicularis). *Primates* 52:117-128.

Latif B, Omar E, Heo CC, Othman N, and Tappe D (2011) Human Pentastomiasis Caused by Armillifer moniliformis in Malaysian Borneo. *The American journal of tropical medicine and hygiene* 85:878-881.

Latif B, Vellayan S, Omar E, Abdullah S, and Mat Desa N (2010) Sarcocystosis among Wild Captive and Zoo Animals in Malaysia. *Korean Journal of Parasitology* 48:213-217.

Lau YL, Chang PY, Subramaniam V, Ng YH, Mahmud R, Ahmad AF, and Fong MY (2013) Genetic assemblage of Sarcocystis spp. in Malaysian snakes. *Parasites & Vectors* 6:257.

Lau YL, Chang PY, Tan CT, Fong MY, Mahmud R, and Wong KT (2014) Sarcocystis nesbitti infection in human skeletal muscle: possible transmission from snakes. *The American journal of tropical medicine and hygiene* 90:361-364.

Lee C, and Amin-Babjee S (1990) Lemdana sonneretta in the Malayan red jungle fowl (Gallus gallus spadiceus). *Malaysian Applied Biology* 19:91-94.

Lee J-I, Kim S-J, and Park C-G (2011a) Shigella flexneri infection in a newly acquired rhesus macaque (Macaca mulatta). *Laboratory animal research* 27:343-346.

Lee K, Iwata T, Nakadai A, Kato T, Hayama S, Taniguchi T, and Hayashidani H (2011b) Prevalence of Salmonella, Yersinia and Campylobacter spp. in Feral Raccoons (Procyon lotor) and Masked Palm Civets (Paguma larvata) in Japan. *Zoonoses and Public Health* 58:424-431.

Lee S-H, Choe E-Y, Shin H-D, and Seo M (2013) Spargana in a weasel, Mustela sibirica manchurica, and a wild boar, Sus scrofa, from Gangwon-do, Korea. *The Korean journal of parasitology* 51:379-381.

Lee SC, Ngui R, Tan TK, Muhammad Aidil R, and Lim YAL (2014) Neglected Tropical Diseases among Two Indigenous Subtribes in Peninsular Malaysia: Highlighting Differences and Co-Infection of Helminthiasis and Sarcocystosis. *PLoS ONE* 9:e107980-e107980.

Leendertz FH, Zirkel F, Couacy-Hymann E, Ellerbrok H, Morozov VA, Pauli G, Hedemann C, Formenty P, Jensen SA, and Boesch C (2008) Interspecies transmission of simian foamy virus in a natural predator-prey system. *Journal of virology* 82:7741-7744.

Lerche NW, Switzer WM, Yee JL, Shanmugam V, Rosenthal AN, Chapman LE, Folks TM, and Heneine W (2001) Evidence of infection with simian type D retrovirus in persons occupationally exposed to nonhuman primates. *Journal of virology* 75:1783-1789.

Li M, Song Y, Li B, Wang Z, Yang R, Jiang L, and Yang R (2005a) Asymptomatic yersinia pestis infection, China. *Emerging infectious diseases* 11:1494-1496.

Li R-C, Xiao C-T, Qian X, Luo W, Ge M, Jiang D-L, and Yu X-L (2012) Occurrence of Streptococcus dysgalactiae subsp. equisimilis in Masked Palm Civet (Paguma larvata). *Journal of Animal and Veterinary Advances* 11:2020-2023.

Li T, Chijiwa K, Sera N, Ishibashi T, Etoh Y, Shinohara Y, Kurata Y, Ishida M, Sakamoto S, and Takeda N (2005b) Hepatitis E virus transmission from wild boar meat. *Emerging infectious diseases* 11:1958-1960.

Li W, Shi Z, Yu M, Ren W, Smith C, Epstein JH, Wang H, Crameri G, Hu Z, and Zhang H (2005c) Bats are natural reservoirs of SARS-like coronaviruses. *Science* 310:676-679.

Lim Y, Ngui R, Shukri J, Rohela M, and Mat Naim H (2008) Intestinal parasites in various animals at a zoo in Malaysia. *Veterinary parasitology* 157:154-159.

Lindsay DS, McKown RD, and Dubey J (2000) Sarcocystis campestris from naturally infected 13-lined ground squirrels, Spermophilus tridecemlineatus tridecemlineatus, from Nebraska. *Journal of Parasitology* 86:1159-1161.

Liss GM, Wong L, Kittle D, Simor A, Naus M, Martiquet P, and Misener C (1993) Occupational exposure to Mycobacterium bovis infection in deer and elk in Ontario. *Canadian journal of public health* 85:326-329.

López C, Panadero R, Bravo A, Paz A, Sánchez-Andrade R, Díez-Banos P, and Morrondo P (2003) Sarcocystis spp. infection in roe deer (Capreolus capreolus) from the north-west of Spain. *Zeitschrift für Jagdwissenschaft* 49:211-218.

Luby SP, Hossain MJ, Gurley ES, Ahmed B-N, Banu S, Khan SU, Homaira N, Rota PA, Rollin PE, and Comer JA (2009) Recurrent zoonotic transmission of Nipah virus into humans, Bangladesh, 2001–2007. *Emerging infectious diseases* 15:1229-1235.

Luby SP, Rahman M, Hossain MJ, Blum LS, Husain MM, Gurley E, Khan R, Ahmed B-N, Rahman S, and Nahar N (2006) Foodborne transmission of Nipah virus, Bangladesh. *Emerging infectious diseases* 12:1888-1894.

Madar CS, Cardile AP, Cunningham S, Magpantay G, and Finger D (2012) A case of Salmonella gastroenteritis following ingestion of raw venison sashimi. *Hawai'i Journal Of Medicine & Public Health: A Journal Of Asia Pacific Medicine & Public Health* 71:49-50.

Magee JS, Steele RW, Kelly NR, and Jacobs RF (1989) Tularemia transmitted by a squirrel bite. *The Pediatric infectious disease journal* 8:123-125.

Magnino S, Colin P, Dei-Cas E, Madsen M, McLauchlin J, Nöckler K, Prieto Maradona M, Tsigarida E, Vanopdenbosch E, and Van Peteghem C (2009) Biological risks associated with consumption of reptile products. *International journal of food microbiology* 134:163-175.

Manolis SC, Webb GJW, Pinch D, Melville L, and Hollis G (1991) Salmonella in captive crocodiles (Crocodylus johnstoni and C. porosus). *Australian Veterinary Journal* 68:102-105.

Martín-Hernando M, Torres MJ, Aznar J, Negro JJ, Gandía A, and Gortázar C (2010) Distribution of Lesions in Red and Fallow Deer Naturally Infected with Mycobacterium bovis. *Journal of Comparative Pathology* 142:43-50.

Massey P, Polkinghorne B, Durrheim D, Lower T, and Speare R (2011) Blood, guts and knife cuts: reducing the risk of swine brucellosis in feral pig hunters in north-west New South Wales, Australia. *Rural and remote health* 11:1-9.

Masuda J-I, Yano K, Tamada Y, Takii Y, Ito M, Omagari K, and Kohno S (2005) Acute hepatitis E of a man who consumed wild boar meat prior to the onset of illness in Nagasaki, Japan. *Hepatology Research* 31:178-183.

Masuzawa T, Okamoto Y, Une Y, Takeuchi T, Tsukagoshi K, Koizumi N, Kawabata H, Ohta S, and Yoshikawa Y (2006) Leptospirosis in squirrels imported from United States to Japan. *Emerging infectious diseases* 12:1153-1155.

Matsuda H, Okada K, Takahashi K, and Mishiro S (2003) Severe hepatitis E virus infection after ingestion of uncooked liver from a wild boar. *Journal of Infectious Diseases* 188:944-944.

Matsumoto T, Ahmed K, Wimalaratne O, Nanayakkara S, Perera D, Karunanayake D, and Nishizono A (2011) Novel sylvatic rabies virus variant in endangered golden palm civet, Sri Lanka. *Emerging infectious diseases* 17:2346-2349.

Matsuura Y, Suzuki M, Yoshimatsu K, Arikawa J, Takashima I, Yokoyama M, Igota H, Yamauchi K, Ishida S, and Fukui D (2007) Prevalence of antibody to hepatitis E virus among wild sika deer, Cervus nippon, in Japan. *Archives of Virology* 152:1375-1381.

McDonald JC, Gyorkos TW, Alberton B, MacLean JD, Richer G, and Juranek D (1990) An outbreak of toxoplasmosis in pregnant women in northern Quebec. *Journal of Infectious Diseases* 161:769-774.

Mehrotra P, Mathur B, Bhargava S, and Chaudhary S (2000) Anthrax in Artiodactylids (Spotted deer-Axis axis) at Zoological Park, Jaipur (Rajasthan)-A Report. *Indian Journal of Comparative Microbiology, Immunology and Infectious Diseases* 21:81-82.

Meng X, Lindsay D, and Sriranganathan N (2009) Wild boars as sources for infectious diseases in livestock and humans. *Philosophical Transactions of the Royal Society: Biological Sciences* 364:2697-2707.

Meyer M (1966) Identification and virulence studies of Brucella strains isolated from Eskimos and reindeer in Alaska, Canada, and Russia. *American journal of veterinary research* 27:353-358.

Michalak K, Austin C, Diesel S, Bacon M, Zimmerman P, and Maslow JN (1998) Mycobacterium tuberculosis infection as a zoonotic disease: transmission between humans and elephants. *Emerging infectious diseases* 4:283-287.

Michitaka K, Takahashi K, Furukawa S, Inoue G, Hiasa Y, Horiike N, Onji M, Abe N, and Mishiro S (2007) Prevalence of hepatitis E virus among wild boar in the Ehime area of western Japan. *Hepatology Research* 37:214-220.

Mihai D, Puchianu G, and Ignea G (2006) Epidemiologic and laboratory diagnostic aspects on rabies in bear. In: *Lucrările sesiuni ştiinţifice Pădurea şi dezvoltarea durabilă*. Braşov, Romania,: Transilvania University of Braşov, pp 285-292

Miko A, Pries K, Haby S, Steege K, Albrecht N, Krause G, and Beutin L (2009) Assessment of Shiga toxin-producing Escherichia coli isolates from wildlife meat as potential pathogens for humans. *Applied and environmental microbiology* 75:6462-6470.

Mikota SK, Peddie L, Peddie J, Isaza R, Dunker F, West G, Lindsay W, Larsen RS, Chatterjee D, and Payeur J (2001) Epidemiology and diagnosis of Mycobacterium tuberculosis in captive Asian elephants (Elephas maximus). *Journal of Zoo and Wildlife Medicine* 32:1-16.

Millán J, Candela MG, López-Bao JV, Pereira M, Jiménez MÁ, and León-Vizcaíno L (2009) Leptospirosis in wild and domestic carnivores in natural areas in Andalusia, Spain. *Vector-Borne and Zoonotic Diseases* 9:549-554.

Min D-Y (1990) Cestode infections in Korea. *Korean Journal of Parasitology* 28:123-144.

Miranda M, Ksiazek T, Retuya T, Khan AS, Sanchez A, Fulhorst CF, Rollin PE, Calaor A, Manalo D, and Roces M (1999) Epidemiology of Ebola (subtype Reston) virus in the Philippines, 1996. *Journal of Infectious Diseases* 179:S115-S119.

Miyazaki S, Ishii T, Matoba S, Awatani T, and Toda I (2001) A case of cat-scratch disease from a masked palm civet in Japan. *Monthly Community Medicine* 15:564-566.

Modric Z, and Huber D (1993) Serologic survey for leptospirae in European brown bears (Ursus arctos) in Croatia. *Journal of Wildlife Diseases* 29:608-611.

Molia S, Chomel BB, Kasten RW, Leutenegger CM, Steele BR, Marker L, Martenson JS, Keet DF, Bengis RG, Peterson RP, Munson L, and O’Brien SJ (2004) Prevalence of Bartonella infection in wild African lions (Panthera leo) and cheetahs (Acinonyx jubatus). *Veterinary Microbiology* 100:31-41.

Mongoh MN, Dyer NW, Stoltenow CL, and Khaitsa ML (2008) Risk factors associated with anthrax outbreak in animals in North Dakota, 2005: A retrospective case-control study. *Public Health Reports* 123:352-359.

Montali R, Smith E, Davenport M, and Bush M (1975) Dermatophilosis in Australian bearded lizards. *Journal of the American Veterinary Medical Association* 167:553-555.

Montes A, Rivera G, Ramírez V, Ríos M, Angulo J, and Muñoz D (2011) Frequency of infection by Leptospira sp. in white neck squirrel (Sciurus stramineus) in a zoo of Lima city. *Revista de Investigaciones Veterinarias del Perú (RIVEP)* 22:66-71.

Moravec F (2001) Some helminth parasites from Morelet's crocodile, Crocodylus moreletii, from Yucatan, Mexico. *Folia Parasitologica* 48:47-62.

More G, Pantchev N, Hermann DC, Vrhovec MG, Öfner S, Conraths FJ, and Schares G (2014) Molecular identification of Sarcocystis spp. helped to define the origin of green pythons (Morelia viridis) confiscated in Germany. *Parasitology* 141:646-651.

Morikawa S, Saijo M, and Kurane I (2007) Current knowledge on lower virulence of Reston Ebola virus *Comparative Immunology, Microbiology and Infectious Diseases* 30:391-398.

Mühldorfer K (2013) Bats and Bacterial Pathogens: A Review. *Zoonoses and Public Health* 60:93-103.

Müller MA, Paweska JT, Leman PA, Drosten C, Grywna K, Kemp A, Braack L, Sonnenberg K, Niedrig M, and Swanepoel R (2007) Coronavirus antibodies in African bat species. *Emerging infectious diseases* 13:1367-1370.

Muñoz PM, Boadella M, Arnal M, de Miguel MJ, Revilla M, Martínez D, Vicente J, Acevedo P, Oleaga Á, and Ruiz-Fons F (2010) Spatial distribution and risk factors of Brucellosis in Iberian wild ungulates. *BMC Infectious Diseases* 10:46.

Murphree R, Warkentin JV, Dunn JR, Schaffner W, and Jones TF (2011) Elephant-to-human transmission of tuberculosis, 2009. *Emerging infectious diseases* 17:366-371.

Murphy HW, Miller M, Ramer J, Travis D, Barbiers R, Wolfe ND, and Switzer WM (2006) Implications of simian retroviruses for captive primate population management and the occupational safety of primate handlers. *Journal of Zoo and Wildlife Medicine* 37:219-233.

Mutalib A, Keirs R, and Austin F (1995) Erysipelas in Quail and Suspected Erysipeloid in Processing Plant Employees. *Avian Diseases* 39:191-193.

Nagano H, Hirochi T, Fujita K, Wakamori Y, Takeshi K, and Yano S (2004) Phenotypic and genotypic characterization of β-d-glucuronidase-positive Shiga toxin-producing Escherichia coli O157 : H7 isolates from deer. *Journal of Medical Microbiology* 53:1037-1043.

Nagel P, Serritella A, and Layden TJ (1982) Edwardsiella tarda Gastroenteritis Associated with a Pet Turtle. *Gastroenterology* 82:1436-1437.

Nakauchi K (1999) The prevalence of Balantidium coli infection in fifty-six mammalian species. *The Journal of veterinary medical science/the Japanese Society of Veterinary Science* 61:63-65.

Nation PN, Fanning EA, Hopf HB, and Church TL (1999) Observations on animal and human health during the outbreak of Mycobacterium bovis in game farm wapiti in Alberta. *The Canadian Veterinary Journal* 40:113-117.

Navarro-Gonzalez N, Casas-Díaz E, Porrero CM, Mateos A, Domínguez L, Lavín S, and Serrano E (2013) Food-borne zoonotic pathogens and antimicrobial resistance of indicator bacteria in urban wild boars in Barcelona, Spain. *Veterinary Microbiology* 167:686-689.

Němejc K, Sak B, Květoňová D, Hanzal V, Jeníková M, and Kváč M (2012) The first report on Cryptosporidium suis and Cryptosporidium pig genotype II in Eurasian wild boars (Sus scrofa) (Czech Republic). *Veterinary parasitology* 184:122-125.

Ng J, Pavlasek I, and Ryan U (2006) Identification of novel Cryptosporidium genotypes from avian hosts. *Applied and environmental microbiology* 72:7548-7553.

Nöckler K, Reckinger S, and Pozio E (2006) Trichinella spiralis and Trichinella pseudospiralis mixed infection in a wild boar (Sus scrofa) of Germany. *Veterinary parasitology* 137:364-368.

Nugent G, Whitford J, Yockney I, and Cross M (2012) Reduced spillover transmission of Mycobacterium bovis to feral pigs (Sus scofa) following population control of brushtail possums (Trichosurus vulpecula). *Epidemiology and infection* 140:1036-1047.

O'Hara TM, Holcomb D, Elzer P, Estepp J, Perry Q, Hagius S, and Kirk C (2010) Brucella species survey in polar bears (Ursus maritimus) of northern Alaska. *Journal of Wildlife Diseases* 46:687-694.

Obanda V, Poghon J, Yongo M, Mulei I, Ngotho M, Waititu K, Makumi J, Gakuya F, Omondi P, and Soriguer RC (2013) First reported case of fatal tuberculosis in a wild African elephant with past human–wildlife contact. *Epidemiology and infection* 141:1476-1480.

Obwegeser T, Stephan R, Hofer E, and Zweifel C (2012) Shedding of foodborne pathogens and microbial carcass contamination of hunted wild ruminants. *Veterinary Microbiology* 159:149-154.

Oksanen A, Åsbakk K, Prestrud K, Aars J, Derocher A, Tryland M, Wiig Ø, Dubey J, Sonne C, and Dietz R (2009) Prevalence of antibodies against Toxoplasma gondii in polar bears (Ursus maritimus) from Svalbard and East Greenland. *Journal of Parasitology* 95:89-94.

Olival KJ, Islam A, Yu M, Anthony SJ, Epstein JH, Khan SA, Khan SU, Crameri G, Wang L-F, and Lipkin WI (2013) Ebola virus antibodies in fruit bats, Bangladesh. *Emerging infectious diseases* 19:270-273.

Ong B, Ngeow Y, RAZAK MA, Yakubu Y, Zakaria Z, Mutalib A, Hassan L, Ng H, and Verasahib K (2013) Tuberculosis in captive Asian elephants (Elephas maximus) in Peninsular Malaysia. *Epidemiology and infection* 141:1481-1487.

Oronan RB, Licuan DA, Licuan DA, Santos JPSD, and Lastica EA (2013) Detection of antibodies against Toxoplasma gondii and Chlamydophila felis in Malayan civets (Viverra tangalunga), Palawan bearcats (Arctictis binturong whitei) and Asian palm civets (Paradoxurus hermaphroditus) at a wildlife facility in Quezon City, Philippines. *Philippine Journal of Veterinary and Animal Sciences* 39:287-292.

Otis V, and Behler J (1973) The occurrence of Salmonellae and Edwardsiella in the turtles of the New York Zoological park. *Journal of Wildlife Diseases* 9:4-6.

Owen IL, Morales MAG, Pezzotti P, and Pozio E (2005) Trichinella infection in a hunting population of Papua New Guinea suggests an ancient relationship between Trichinella and human beings. *Transactions of the Royal Society of Tropical Medicine and Hygiene* 99:618-624.

Pagès-Manté A, Pagès-Bosch M, Majó-Masferrer N, Gómez-Couso H, and Ares-Mazás E (2007) An outbreak of disease associated with cryptosporidia on a red-legged partridge (Alectoris rufa) game farm. *Avian Pathology* 36:275-278.

Pandit S (1950) Two instances of proved rabies in the tiger. *Indian medical gazette* 85:441.

Park HY, Lee SU, Kim SH, Lee PC, Huh S, Yang YS, and Kong Y (2001) Epidemiological significance of sero-positive inhabitants against sparganum in Kangwon-do, Korea. *Yonsei medical journal* 42:371-374.

Patrick ME, Gilbert MJ, Blaser MJ, Tauxe RV, Wagenaar JA, and Fitzgerald C (2013) Human infections with new subspecies of Campylobacter fetus. *Emerging infectious diseases* 19:1678-1680.

Paul-Murphy J, Work T, Hunter D, McFie E, and Fjelline D (1994) Serologic survey and serum biochemical reference ranges of the free-ranging mountain lion (Felis concolor) in California. *Journal of Wildlife Diseases* 30:205-215.

Pavlik I, Machackova M, Yayo Ayele W, Lamka J, Parmova I, Melicharek I, Hanzlikova M, Kormendy B, Nagy G, and Cvetnic Z (2002) Incidence of bovine tuberculosis in wild and domestic animals other than cattle in six Central European countries during 1990-1999. *Veterinari Medicina Praha* 47:122-131.

Pavlov PM (1988) Health risks to humans and domestic livestock posed by feral pigs (Sus scrofa) in north Queensland. In: *Proceedings of the 13th Vertebrate Pest Conference*. San Diego, California, USA: University of California, Davies, pp 141-144

Perz JF, and Le Blancq SM (2001) Cryptosporidium parvum infection involving novel genotypes in wildlife from lower New York State. *Applied and environmental microbiology* 67:1154-1162.

Pettit J, Gough A, and Truscott R (1976) Erysipelothrix rhusiopathiae infection in chukar partridge (Alectoris graeca). *Journal of Wildlife Diseases* 12:254-255.

Pioz M, Loison A, Gibert P, Jullien J-M, Artois M, and Gilot-Fromont E (2008) Antibodies against Salmonella is associated with reduced reproductive success in female alpine chamois (Rupicapra rupicapra). *Canadian journal of zoology* 86:1111-1120.

Portaels F, Realini L, Bauwens L, Hirschel B, Meyers W, and De Meurichy W (1996) Mycobacteriosis caused by Mycobacterium genavense in birds kept in a zoo: 11-year survey. *Journal of clinical microbiology* 34:319-323.

Pourrut X, Diffo J, Somo R, Bilong Bilong C, Delaporte E, LeBreton M, and Gonzalez J-P (2011) Prevalence of gastrointestinal parasites in primate bushmeat and pets in Cameroon. *Veterinary parasitology* 175:187-191.

Pozio E, Foggin CM, Gelanew T, Marucci G, Hailu A, Rossi P, and Morales MAG (2007) Trichinella zimbabwensis in wild reptiles of Zimbabwe and Mozambique and farmed reptiles of Ethiopia. *Veterinary parasitology* 143:305-310.

Pozio E, Owen I, La Rosa G, Sacchi L, Rossi P, and Corona S (1999) Trichinella papuae n. sp.(Nematoda), a new non-encapsulated species from domestic and sylvatic swine of Papua New Guinea. *International journal for parasitology* 29:1825-1839.

Pozio E, Owen IL, Marucci G, and La Rosa G (2004) Trichinella papuae in saltwater crocodiles (Crocodylus porosus) of Papua New Guinea. *Emerging infectious diseases* 10:1507-1509.

Prathap K, Lau K, and Bolton J (1969) Pentastomiasis: a common finding at autopsy among Malaysian aborigines. *The American journal of tropical medicine and hygiene* 18:20-27.

Prestrud P, Krogsrud J, and Gjertz I (1992) The occurrence of rabies in the Svalbard Islands of Norway. *Journal of Wildlife Diseases* 28:57-63.

Pritchard LI, Chua KB, Cummins D, Hyatt A, Crameri G, Eaton BT, and Wang LF (2006) Pulau virus; a new member of the Nelson Bay orthoreovirus species isolated from fruit bats in Malaysia. *Archives of Virology* 151:229-239.

Priya P, Mini M, Rameshkumar P, and Jayesh V (2009) A case of anthrax in wild elephant from the Western Ghats region of Kerala, India. *Journal of Threatened Taxa* 1:192-193.

ProMED-mail. 2001. Anthrax, deer, bison, human - USA (Texas), Archive Number: 20010703.1278. ProMED-mail 3 Jul 2001. <http://www.promedmail.org/>. (Accessed 24 Feb 2015).

ProMED-mail. 2003. Plague, mule deer - USA (Montana), Archive Number: 20030911.2286. ProMED-mail 11 Sep 2003. <http://www.promedmail.org/>. (Accessed 24 Feb 2015).

ProMED-mail. 2005. Avian influenza - Asia (12): Viet Nam, civets, H5N1, Archive Number: 20050826.2527. ProMED-mail 26 Aug 2005. <http://www.promedmail.org/>. (Accessed 24 Feb 2015).

ProMED-mail. 2008. Avian influenza (47): Viet Nam, civet , Archive Number: 20080316.1035. ProMed-mail 16 Mar 2008. <http://www.promedmail.org/>. (Accessed 24 Feb 2015).

ProMED-mail. 2009. Rabies - Tanzania (Serengeti National Park), civet, human exp., novel lyssavirus, Archive Number: 20120314.1070293. ProMED-mail 14 Mar 2009. <http://www.promedmail.org/>. (Accessed 24 Feb 2015).

ProMED-mail. 2010a. Plague, cougar - USA (Wyoming). <http://www.promedmail.org/> Accessed 25 Feb 2015., Archive Number: 20100208.0429.

ProMED-mail. 2010b. Tuberculosis, elephants – Nepal. <http://www.promedmail.org/> Accessed 25 Feb 2015., Archive Number: 20100520.1665.

ProMED-mail. 2011. Anthrax, human, livestock - India (06): (AP) wildlife, Archive Number: 20110802.2328. ProMED-mail 2 Aug 2011. <http://www.promedmail.org/>. (Accessed 24 Feb 2015).

ProMED-mail. 2013. Plague, animal - USA (05): (California) squirrel, Archive Number: 20130927.1971423. ProMed-mail 27 Sep 2013. <http://www.promedmail.org/>. (Accessed 24 Feb 2015).

ProMED-mail. 2014a. Australian bat lyssavirus - Australia (03): (NS) flying fox, human exp, Archive Number: 20141025.2900858**.** ProMED-mail 25 Oct 2014. <http://www.promedmail.org/> (Accessed 25 Feb 2015).

ProMED-mail. 2014b. Rabies - Costa Rica: (Puntarenas) human, squirrel, Archive Number: 20140728.2641077. ProMED-mail 28 Jul 2014. <http://www.promedmail.org/> (Accessed 24 Feb 2015). .

ProMED-mail. 2014c. Rabies - Taiwan (03): (PT) Formosan gem-faced civet, OIE, Archive Number: 20141230.3063405. ProMED-mail 30 Dec 2014. <http://www.promedmail.org/>. (Accessed 24 Feb 2015).

Puvanesuaran VR, Noordin R, and Balakrishnan V (2013) Genotyping of Toxoplasma gondii isolates from wild boars in Peninsular Malaysia. *PLoS ONE* 8:e61730.

Rabatsky-Ehr T, Dingman D, Marcus R, Howard R, Kinney A, and Mshar P (2002) Deer meat as the source for a sporadic case of Escherichia coli O157: H7 infection. *Emerging infectious diseases* 8:525-527.

Ramasoota T (1991) Current status of food-borne parasitic zoonoses in Thailand. *Southeast Asian Journal of Tropical Medicine Public Health* 22:23-26.

Randall C (1986) Renal and nasal cryptosporidiosis in a junglefowl (Gallus sonneratii). *Veterinary Record* 119:130-131.

Ranque S, Faugère B, Pozio E, La Rosa G, Tamburrini A, Pellissier J-F, and Brouqui P (2000) Trichinella pseudospiralis outbreak in France. *Emerging infectious diseases* 6:543-547.

Renter DG, Gnad DP, Sargeant JM, and Hygnstrom SE (2006) Prevalence and Serovars of Salmonella in the Feces of Free-Ranging White-Tailed Deer Odocoileus virginianus in Nebraska. *Journal of Wildlife Diseases* 42:699-703.

Renter DG, Sargeant JM, Hygnstorm SE, and Hoffman JD (2001) Escherichia coli O157: H7 in free-ranging deer in Nebraska. *Journal of Wildlife Diseases* 37:755-760.

Reperant L, Rimmelzwaan G, and Kuiken T (2009) Avian influenza viruses in mammals. *Revue scientifique et technique* 28:137-159.

Reynes J-M, Molia S, Audry L, Hout S, Ngin S, Walston J, and Bourhy H (2004) Serologic evidence of lyssavirus infection in bats, Cambodia. *Emerging infectious diseases* 10:2231-2234.

Rhyan J, and Saari D (1995) A comparative study of the histopathologic features of bovine tuberculosis in cattle, fallow deer (Dama dama), sika deer (Cervus nippon), and red deer and elk (Cervus elaphus). *Veterinary Pathology Online* 32:215-220.

Ribicich M, Gamble H, Bolpe J, Scialfa E, Krivokapich S, Cardillo N, Betti A, Holzmann MLC, Pasqualetti M, and Fariña F (2010) Trichinella infection in wild animals from endemic regions of Argentina. *Parasitology research* 107:377-380.

Rickard LG, Siefker C, Boyle CR, and Gentz EJ (1999) The prevalence of Cryptosporidium and Giardia spp. in fecal samples from free-ranging white-tailed deer (Odocoileus virginianus) in the southeastern United States. *Journal of veterinary diagnostic investigation* 11:65-72.

Riley J, and Huchzermeyer FW (1995) Pentastomid parasites of the family Sebekidae Fain, 1961 in West African dwarf crocodiles Osteolaemus tetraspis Cope, 1851 from the Congo, with a description of Alofia parva n. sp. *Onderstepoort Journal of Veterinary Research* 62:151-162.

Riley J, and Self J (1981) Some observations on the taxonomy and systematics of the pentastomid genus Armillifer (Sambon, 1922) in South East Asian and Australian snakes. *Systematic Parasitology* 2:171-179.

Risco D, Llario P, Velarde R, García W, Benítez J, García A, Bermejo F, Cortés M, Rey J, and De Mendoza J (2011) Outbreak of Swine Erysipelas in a Semi‐Intensive Wild Boar Farm in Spain. *Transboundary and Emerging Diseases* 58:445-450.

Robson J, Harrison M, Wood R, Tilse M, McKay A, and Brodribb T (1993) Brucellosis: re-emergence and changing epidemiology in Queensland. *The Medical Journal of Australia* 159:153-158.

Rodríguez dlPE, Rodriguez-Ferrer M, Nieto-Martinez J, Ubeira F, and Garate-Ormaechea T (2004) Trichinellosis outbreaks in Spain (1990-2001). *Enfermedades infecciosas y microbiologia clinica* 22:70-76.

Roess AA, Galan A, Kitces E, Li Y, Zhao H, Paddock CD, Adem P, Goldsmith CS, Miller D, and Reynolds MG (2010) Novel deer-associated parapoxvirus infection in deer hunters. *New England Journal of Medicine* 363:2621-2627.

Rohela M, Lim Y, Jamaiah I, Khadijah P, Laang S, Nazri M, and Nurulhuda Z (2005) Occurrence of Cryptosporidium oocysts in Wrinkled Hornbill and other birds in the Kuala Lumpur National Zoo. *Southeast Asian Journal of Tropical Medicine Public Health* 36:34-40.

Rollin PE, Williams RJ, Bressler DS, Pearson S, Cottingham M, Pucak G, Sanchez A, Trappier SG, Peters RL, and Greer PW (1999) Ebola (subtype Reston) virus among quarantined nonhuman primates recently imported from the Philippines to the United States. *Journal of Infectious Diseases* 179:S108-S114.

Rosenkranz M, Elsner H-A, Stürenburg HJ, Weiller C, Röther J, and Sobottka I (2003) Streptococcus suis meningitis and septicemia contracted from a wild boar in Germany. *Journal of neurology* 250:869-870.

Ross RD, Stec LA, Werner JC, Blumenkrank MS, L. G, and Williams GA (2001) Presumed acquired ocular toxoplasmosis in deer hunters. *Retina* 21:226-229.

Rotstein DS, Taylor SK, Bradley J, and Breitschwerdt EB (2000) Prevalence of Bartonella henselae antibody in Florida panthers. *Journal of Wildlife Diseases* 36:157-160.

Rounds JM, Rigdon CE, Muhl LJ, Forstner M, Danzeisen GT, Koziol BS, Taylor C, Shaw BT, Short GL, and Smith KE (2012) Non-O157 Shiga toxin-producing Escherichia coli associated with venison. *Emerging infectious diseases* 18:279-282.

Roy P, Venugopalan AT, Selvarangam R, and Ramaswamy V (1998) Velogenic Newcastle Disease Virus in Captive Wild Birds. *Tropical animal health and production* 30:299-303.

Ruppanner R, Jessup D, Ohishi I, Behymer D, and Franti C (1982) Serologic survey for certain zoonotic diseases in black bears in California. *Journal of the American Veterinary Medical Association* 181:1288-1291.

Rutjes S, Lodder-Verschoor F, Lodder W, Van der Giessen J, Reesink H, Bouwknegt M, and de Roda Husman A (2010) Seroprevalence and molecular detection of hepatitis E virus in wild boar and red deer in The Netherlands. *Journal of virological methods* 168:197-206.

Sacks JJ, Delgado DG, Lobel HO, and Parker RL (1983) Toxoplasmosis infection associated with eating undercooked venison. *American journal of epidemiology* 118:832-838.

Sakae C, and Ishida T (2012) Direct Evidence for Toxoplasma gondii Infection in a Wild Serow (Capricornis crispus) From Mainland Japan. *Journal of Parasitology* 98:224-225.

Saliki JT, Rodgers SJ, and Eskew G (1998) Serosurvey of selected viral and bacterial diseases in wild swine from Oklahoma. *Journal of Wildlife Diseases* 34:834-838.

Salinas J, Caro MR, Vicente J, Cuello F, Reyes-Garcia AR, Buendía AJ, Rodolakis A, and Gortázar C (2009) High prevalence of antibodies against Chlamydiaceae and Chlamydophila abortus in wild ungulates using two “in house” blocking-ELISA tests. *Veterinary Microbiology* 135:46-53.

Salkeld D, and Stapp P (2006) Seroprevalence rates and transmission of plague (Yersinia pestis) in mammalian carnivores. *Vector-Borne & Zoonotic Diseases* 6:231-239.

Samaha H, Haggag Y, Nossair M, and Hashim A (2012) Serological invesstigation of Salmonellosis among some wild birds and human contacts. *Alexandria Journal of Veterinary Sciences* 35:103-111.

Samaratunga, Searle, and Hudson (1998) Non–rabies Lyssavirus human encephalitis from fruit bats: Australian bat Lyssavirus (pteropid Lyssavirus) infection. *Neuropathology and Applied Neurobiology* 24:331-335.

Samra NA, Jori F, Samie A, and Thompson P (2011) The prevalence of Cryptosporidium spp. oocysts in wild mammals in the Kruger National Park, South Africa. *Veterinary parasitology* 175:155-159.

Sánchez S, García-Sánchez A, Martínez R, Blanco J, Blanco J, Blanco M, Dahbi G, Mora A, Hermoso de Mendoza J, and Alonso J (2009) Detection and characterisation of Shiga toxin-producing Escherichia coli other than Escherichia coli O157: H7 in wild ruminants. *The Veterinary Journal* 180:384-388.

Sánchez S, Martínez R, García A, Vidal D, Blanco J, Blanco M, Blanco J, Mora A, Herrera-León S, and Echeita A (2010) Detection and characterisation of O157: H7 and non-O157 Shiga toxin-producing Escherichia coli in wild boars. *Veterinary Microbiology* 143:420-423.

Sarma K, Bhawal A, Yadav V, Saikia G, and Das J (2006) Investigation of tuberculosis in captive Asian elephants of Assam vis-a-vis its cross infections with the handlers. *Intas Polivet* 7:269-274.

Sato S, Kabeya H, Shigematsu Y, Sentsui H, Une Y, Minami M, Murata K, Ogura G, and Maruyama S (2013) Small Indian mongooses and masked palm civets serve as new reservoirs of Bartonella henselae and potential sources of infection for humans. *Clinical Microbiology and Infection* 19:1181-1187.

Sato Y, Kobayashi C, Ichikawa K, Kuwamoto R, Matsuura S, and Koyama T (2000) An Occurrence of Salmonella Typhimurium Infection in Sika Deer(Cervus nippon). *Journal of Veterinary Medical Science* 62:313-315.

Sato Y, Sato H, Naka K, Furuya S, Tsukiji H, Kitagawa K, Sonoda Y, Usui T, Sakamoto H, and Yoshino S (2011) A nationwide survey of hepatitis E virus (HEV) infection in wild boars in Japan: identification of boar HEV strains of genotypes 3 and 4 and unrecognized genotypes. *Archives of Virology* 156:1345-1358.

Saxena M, Singh V, Kumar A, Chaudhuri P, Singh VP, Shivachandra S, Biswas A, and Sharma B (2006) REP-PCR analysis of Pasteurella multocida isolates from wild and domestic animals in India. *Veterinary research communications* 30:851-861.

Schellenberg RS, Tan BJ, Irvine JD, Stockdale DR, Gajadhar AA, Serhir B, Botha J, Armstrong CA, Woods SA, and Blondeau JM (2003) An outbreak of trichinellosis due to consumption of bear meat infected with Trichinella nativa in 2 northern Saskatchewan communities. *Journal of Infectious Diseases* 188:835-843.

Schierack P, Römer A, Jores J, Kaspar H, Guenther S, Filter M, Eichberg J, and Wieler LH (2009) Isolation and characterization of intestinal Escherichia coli clones from wild boars in Germany. *Applied and environmental microbiology* 75:695-702.

Schmitt SM, Fitzgerald SD, Cooley TM, Bruning-Fann CS, Sullivan L, Berry D, Carlson T, Minnis RB, Payeur JB, and Sikarskie J (1997) Bovine tuberculosis in free-ranging white-tailed deer from Michigan. *Journal of Wildlife Diseases* 33:749-758.

Schweizer M, Falcone V, Gänge J, Turek R, and Neumann-Haefelin D (1997) Simian foamy virus isolated from an accidentally infected human individual. *Journal of virology* 71:4821-4824.

Seguchi K, Matsuno M, Kataoka H, Kobayashi T, Maruyama H, Itoh H, Koono M, and Nawa Y (1995) A case report of colonic ileus due to eosinophilic nodular lesions caused by Gnathostoma doloresi infection. *The American journal of tropical medicine and hygiene* 53:263-266.

Sestak K, Merritt CK, Borda J, Saylor E, Schwamberger SR, Cogswell F, Didier ES, Didier PJ, Plauche G, and Bohm RP (2003) Infectious agent and immune response characteristics of chronic enterocolitis in captive rhesus macaques. *Infection and immunity* 71:4079-4086.

Shah K, V. (1972) Evidence for an SV40-related papovavirus infection of man. *American journal of epidemiology* 95:199-206.

Sharma P, Kumar V, Pal B, Mandial R, Jithendran K, and Rastogi S (2013) Prevalence of gastrointestinal parasitism in free ranging rhesus macaque (Macaca mulatta) of Himachal Pradesh. *Magazine of Zoo Outreach Organization* 45:26.

Shayegani M, Stone W, DeForge I, Root T, Parsons L, and Maupin P (1986) Yersinia enterocolitica and related species isolated from wildlife in New York State. *Applied and environmental microbiology* 52:420-424.

Shi Z, and Hu Z (2008) A review of studies on animal reservoirs of the SARS coronavirus. *Virus research* 133:74-87.

Singh S, Singh A, Singh P, Kumar A, and Singh B (2011) Molecular identification and characterization of Mycobacterium avium subspecies paratuberculosis in free living non-human primate (Rhesus macaques) from North India. *Comparative Immunology, Microbiology and Infectious Diseases* 34:267-271.

Slavica A, Konjevic D, Huber Đ, Milas Z, Turk N, Sindicic M, Severin K, Dezđek D, and Mašek T (2010) Serologic evidence of Leptospira spp. serovars in brown bears (Ursus arctos) from Croatia. *Journal of Wildlife Diseases* 46:251-256.

Smith CG, Turner L, Harrison J, and Broom J (1961) Animal leptospirosis in Malaya: 1. methods, zoogeographical background, and broad analysis of results. *Bulletin of the World Health Organization* 24:5.

Smith KJ, Skelton HG, James WD, and Lupton GP (1991) Parapoxvirus infections acquired after exposure to wildlife. *Archives of dermatology* 127:79-82.

Smythe LD, Field HE, Barnett LJ, Smith C, Dohnt MF, Symonds ML, Moore MR, and Rolfe P (2002) Leptospiral antibodies in flying foxes in Australia. *Journal of Wildlife Diseases* 38:182-186.

Sohn W-M, and Lee S-H (1998) The first discovery of larva1'Gnathostoma hispidum (Nematoda: Gnathostomidae) from Agkistrodon brevicaudus. *The Korean journal of parasitology* 36:81-89.

Solaymani-Mohammadi S, Mobedi I, Rezaian M, Massoud J, Mohebali M, Hooshyar H, Ashrafi K, and Rokni M (2003) Helminth parasites of the wild boar, Sus scrofa, in Luristan province, western Iran and their public health significance. *Journal of helminthology* 77:263-268.

Solaymani-Mohammadi S, Rezaian M, Hooshyar H, Mowlavi GR, Babaei Z, and Anwar MA (2004) Intestinal Protozoa in Wild Boars (Sus scrofa) in Western Iran. *Journal of Wildlife Diseases* 40:801-803.

Stallknecht DE, Brown JD, and Swayne D (2008) Ecology of avian influenza in wild birds. In: *Avian influenza,* Swayne DE (editor), Iowa, USA: Blackwell Publishing pp 43-58

Starnes C, Talwani R, Horvath J, Duffus W, and Bryan C (2004) Brucellosis in two hunt club members in South Carolina. *Journal of the South Carolina Medical Association 1975* 100:113-115.

Stevenson HL, Bai Y, Kosoy MY, Montenieri JA, Lowell JL, Chu MC, and Gage KL (2003) Detection of novel Bartonella strains and Yersinia pestis in prairie dogs and their fleas (Siphonaptera: Ceratophyllidae and Pulicidae) using multiplex polymerase chain reaction. *Journal of Medical Entomology* 40:329-337.

Summer R, Ross S, and Kiehl W (2004) Imported case of rabies in Germany from India. *Euro Surveillance* 8:2585.

Susetya H, Sugiyama M, Inagaki A, Ito N, Mudiarto G, and Minamoto N (2008) Molecular epidemiology of rabies in Indonesia. *Virus research* 135:144-149.

Suzuki T, Minamoto N, Sugiyama M, Kinjo T, Suzuki Y, Sugimura M, and Atoji Y (1993) Isolation and antibody prevalence of a parapoxvirus in wild Japanese serows (Capricornis crispus). *Journal of Wildlife Diseases* 29:384-389.

Swayne D, and Suarez D (2000) Highly pathogenic avian influenza. *Revue scientifique et technique (International Office of Epizootics)* 19:463-482.

Taema MM, Bull JC, Macgregor SK, Flach EJ, Boardman WS, and Routh AD (2008) Retrospective study of Campylobacter infection in a zoological collection. *Applied and environmental microbiology* 74:1332-1338.

Takahashi K, Kitajima N, Abe N, and Mishiro S (2004) Complete or near-complete nucleotide sequences of hepatitis E virus genome recovered from a wild boar, a deer, and four patients who ate the deer. *Virology* 330:501-505.

Takano J-i, Narita T, Tachibana H, Shimizu T, Komatsubara H, Terao K, and Fujimoto K (2005) Entamoeba histolytica and Entamoeba dispar infections in cynomolgus monkeys imported into Japan for research. *Parasitology research* 97:255-257.

Tanaka S, Maruyama H, Ishiwata K, and Nawa Y (1997) A case report of pleural sparganosis. *Parasitology International* 46:73-75.

Taniguchi S, Watanabe S, Masangkay JS, Omatsu T, Ikegami T, Alviola P, Ueda N, Iha K, Fujii H, and Ishii Y (2011) Reston Ebolavirus antibodies in bats, the Philippines. *Emerging infectious diseases* 17:1559-1560.

Tappe D, Abdullah S, Heo C, Kannan Kutty M, and Latif B (2013) Review Paper Human and animal invasive muscular sarcocystosis in Malaysia–recent cases, review and hypotheses. *Tropical biomedicine* 30:355-366.

Taylor M, Elkin B, Maier N, and Bradley M (1991) Observation of a polar bear with rabies. *Journal of Wildlife Diseases* 27:337-339.

Tei S, Kitajima N, Ohara S, Inoue Y, Miki M, Yamatani T, Yamabe H, Mishiro S, and Kinoshita Y (2004) Consumption of uncooked deer meat as a risk factor for hepatitis E virus infection: An age‐and sex‐matched case‐control study. *Journal of medical virology* 74:67-70.

Tei S, Kitajima N, Takahashi K, and Mishiro S (2003) Zoonotic transmission of hepatitis E virus from deer to human beings. *The Lancet* 362:371-373.

Teo C (2010) Much meat, much malady: changing perceptions of the epidemiology of hepatitis E. *Clinical Microbiology and Infection* 16:24-32.

Tessaro SV (1986) The existing and potential importance of brucellosis and tuberculosis in Canadian wildlife: A review. *The Canadian Veterinary Journal* 27:119-124.

Thakur S, Sandfoss M, Kennedy-Stoskopf S, and DePerno CS (2011) Detection of Clostridium difficile and Salmonella in feral swine population in North Carolina. *Journal of Wildlife Diseases* 47:774-776.

Thanawongnuwech R, Amonsin A, Tantilertcharoen R, Damrongwatanapokin S, Theamboonlers A, Payungporn S, Nanthapornphiphat K, Ratanamungklanon S, Tunak E, and Songserm T (2005) Probable tiger-to-tiger transmission of avian influenza H5N1. *Emerging infectious diseases* 11:699-701.

Thayaparan S, Robertson I, Amraan F, Su’ut L, and Abdullah M (2013) Serological prevalence of Leptospiral infection in wildlife in Sarawak, Malaysia. *Borneo Journal of Resource Science and Technology* 2:79-82.

Thiangtum K, Nimsuphun B, Pinyopanuwat N, Chimnoi W, Tunwattana W, Tongthainan D, Jittapalapong S, Rukkwamsuk T, and Maruyama S (2006) Seroprevalence of Toxoplasma gondii in captive felids in Thailand. *Veterinary parasitology* 136:351-355.

Tian M, Chen Y, Wu L, Rosenthal BM, Liu X, He Y, Dunams DB, Cui L, and Yang Z (2012) Phylogenetic analysis of Sarcocystis nesbitti (Coccidia: Sarcocystidae) suggests a snake as its probable definitive host. *Veterinary parasitology* 183:373-376.

Tomiyama D, Inoue E, Osawa Y, and Okazaki K (2009) Serological evidence of infection with hepatitis E virus among wild Yezo‐deer, Cervus nippon yesoensis, in Hokkaido, Japan. *Journal of viral hepatitis* 16:524-528.

Toyoda K, Furusyo N, Takeoka H, Murata M, Sawayama Y, and Hayashi J (2008) Epidemiological study of hepatitis E virus infection in the general population of Okinawa, Kyushu, Japan. *Journal Of Gastroenterology And Hepatology* 23:1885-1890.

Trávniček M, Čisláková L, and Miško J (2000) Presence of antibodies to Chlamydia psittaci in farm-managed pheasants (Phasianus colchicus) and pigeons (Columba livia). *Veterinární medicína* 45:149-151.

Tresierra-Ayala Á, Espinoza F, Bendayán ME, Donayre M, and Fernández H (2006) La fauna silvestre de la Amazonía peruana, un potencial reservorio de Campylobacter jejuni subsp. jejuni y Campylobacter coli. *Folia Amazónica* 15:117-122.

Tribe G, and Fleming M (1983) Biphasic enteritis in imported cynomolgus (Macaca fascicularis) monkeys infected with Shigella, Salmonella and Campylobacter species. *Laboratory Animals* 17:65-69.

Tryland M, Derocher AE, Wiig Ø, and Godfroid J (2001) Brucella sp. antibodies in polar bears from Svalbard and the Barents Sea. *Journal of Wildlife Diseases* 37:523-531.

Tu C, Crameri G, Kong X, Chen J, Sun Y, Yu M, Xiang H, Xia X, Liu S, and Ren T (2004a) Antibodies to SARS coronavirus in civets. *Emerging infectious diseases* 10:2244-2248.

Tu Z-C, Zeitlin G, Gagner J-P, Keo T, Hanna BA, and Blaser MJ (2004b) Campylobacter fetus of reptile origin as a human pathogen. *Journal of clinical microbiology* 42:4405-4407.

Uhart MM, Vila AR, Beade MS, Balcarce A, and Karesh WB (2003) Health evaluation of pampas deer (Ozotoceros bezoarticus celer) at Campos del Tuyu Wildlife Reserve, Argentina. *Journal of Wildlife Diseases* 39:887-893.

Ullmann LS, Hoffmann JL, de Moraes W, Cubas ZS, Santos LCd, da Silva RC, Moreira N, Guimaraes AMS, Camossi LG, and Langoni H (2012) Serologic survey for Leptospira spp. in captive neotropical felids in Foz do Iguaçu, Paraná, Brazil. *Journal of Zoo and Wildlife Medicine* 43:223-228.

Une Y, and Mori T (2007) Tuberculosis as a zoonosis from a veterinary perspective. *Comparative Immunology, Microbiology and Infectious Diseases* 30:415-425.

Vanrompay D, Andersen A, Ducatelle R, and Haesebrouck F (1993) Serotyping of European isolates of Chlamydia psittaci from poultry and other birds. *Journal of clinical microbiology* 31:134-137.

Vashi NA, Reddy P, Wayne DB, and Sabin B (2010) Bat-associated leptospirosis. *Journal of general internal medicine* 25:162-164.

Vengust G, Valencak Z, and Bidovec A (2006) A serological survey of selected pathogens in wild boar in Slovenia. *Journal of Veterinary Medicine, Series B* 53:24-27.

Vicente Baños J, León-Vizcaíno L, Gortázar C, Cubero MJ, González M, and Martín-Atance P (2002) Antibodies to selected viral and bacterial pathogens in European wild boars from southcentral Spain. *Journal of Wildlife Diseases* 38:649-652.

Vicente J, Höfle U, Garrido JM, Fernández-De-Mera IG, Juste R, Barral M, and Gortazar C (2006) Wild boar and red deer display high prevalences of tuberculosis-like lesions in Spain. *Veterinary Research* 37:107-119.

Vieira-Pinto M, Morais L, Caleja C, Themudo P, Torres C, Igrejas G, Poeta P, and Martins C (2011) Salmonella sp. in game (Sus scrofa and Oryctolagus cuniculus). *Foodborne pathogens and disease* 8:739-740.

Vikøren T, Tharaldsen J, Fredriksen B, and Handeland K (2004) Prevalence of Toxoplasma gondii antibodies in wild red deer, roe deer, moose, and reindeer from Norway. *Veterinary parasitology* 120:159-169.

Vinhas BMR (2014) Sanitary evaluation of large game hunted in Idanha-a-Nova county: pilot study on evaluation of Sarcocystis spp. in muscular samples from large game harvested for human consumption. Universidade de Trás-os-Montes e Alto Douro, Villa Real, Spain.

Wafula MM, Patrick A, and Charles T (2008) Managing the 2004/05 anthrax outbreak in Queen Elizabeth and Lake Mburo National Parks, Uganda. *African Journal of Ecology* 46:24-31.

Wakelin C, and Churchman O (1991) Prevalence of bovine tuberculosis in feral pigs in Central Otago. *Surveillance* 18:19-20.

Wang C-M, Shia W-Y, Jhou Y-J, and Shyu C-L (2013a) Occurrence and molecular characterization of reptilian Campylobacter fetus strains isolated in Taiwan. *Veterinary Microbiology* 164:67-76.

Wang C-M, Shia W-Y, Jhou Y-J, and Shyu C-L (2013b) Occurrence and molecular characterization of reptilian Campylobacter fetus strains isolated in Taiwan. *Veterinary Microbiology* 164:67-76.

Wang M, Yan M, Xu H, Liang W, Kan B, Zheng B, Chen H, Zheng H, Xu Y, and Zhang E (2005) SARS-CoV infection in a restaurant from palm civet. *Emerging infectious diseases* 11:1860-1865.

Wang ZQ, Cui J, and Shen LJ (2007) The epidemiology of animal trichinellosis in China. *The Veterinary Journal* 173:391-398.

Ward MP, Cowled BD, Galea F, Garner MG, Laffan SW, Marsh I, Negus K, Sarre SD, and Woolnough AP (2013) Salmonella infection in a remote, isolated wild pig population. *Veterinary Microbiology* 162:921-929.

Warrilow D, Smith IL, Harrower B, and Smith GA (2002) Sequence Analysis of an Isolate from a Fatal Human Infection of Australian Bat Lyssavirus. *Virology* 297:109-119.

Watarai M, Ito N, Omata Y, and Ishiguro N (2006) A serological survey of Brucella spp. in free-ranging wild boar (Sus scrofa leucomystax) in Shikoku, Japan. *The Journal of veterinary medical science/the Japanese Society of Veterinary Science* 68:1139-1141.

Wazed Ali Mollah M, and McKinney P (2002) Brucellosis and suspected Paratuberculosis in a Nubian ibex (Capra Ibex Nubiana) A case report. In: *Proceedings of the World Association of Wildlife Veterinarians Wildlife Sessions at 27th World Veterinary Congress*. Tunisia:

Webster WA, Casey GA, and Charlton KM (1988) Ontario. Rabies in a squirrel. *The Canadian Veterinary Journal* 29:1015-1015.

Weigler BJ (1992) Biology of B virus in macaque and human hosts: a review. *Clinical Infectious Diseases* 14:555-567.

Wellehan JFX, Turenne C, Heard DJ, Detrisac CJ, and O'Kelley JJ (2004) Dermatophilus chelonae in a King Cobra (Ophiophagus hannah). *Journal of Zoo and Wildlife Medicine* 35:553-556.

Wenz-Muecke A, Sithithaworn P, Petney TN, and Taraschewski H (2013) Human contact influences the foraging behaviour and parasite community in long-tailed macaques. *Parasitology* 140:709-718.

Wichmann O, Schimanski S, Koch J, Kohler M, Rothe C, Plentz A, Jilg W, and Stark K (2008) Phylogenetic and case-control study on hepatitis E virus infection in Germany. *Journal of Infectious Diseases* 198:1732-1741.

Wilbur AK, Engel GA, Rompis A, Putra IA, LEE BPH, Aggimarangsee N, Chalise M, Shaw E, Oh G, and Schillaci MA (2012) From the mouths of monkeys: Detection of Mycobacterium tuberculosis complex DNA from buccal swabs of synanthropic macaques. *American journal of primatology* 74:676-686.

Wilde H, Chutivongse S, Tepsumethanon W, Choomkasien P, Polsuwan C, and Lumbertdacha B (1991) Rabies in Thailand: 1990. *Reviews of Infectious Diseases* 13:644-652.

Wilkins M, Bartlett P, Frawley B, o'Brien D, Miller C, and Boulton M (2003) Mycobacterium bovis (bovine TB) exposure as a recreational risk for hunters: results of a Michigan Hunter Survey, 2001. *The International Journal of Tuberculosis and Lung Disease* 7:1001-1009.

Wilkins MJ, Meyerson J, Bartlett PC, Spieldenner SL, Berry DE, Mosher LB, Kaneene JB, Robinson-Dunn B, Stobierski MG, and Boulton ML (2008) Human Mycobacterium bovis infection and bovine tuberculosis outbreak, Michigan, 1994–2007. *Emerging infectious diseases* 14:657-660.

Wisser J, Rudolph M, Frolich K, Pilaski J, Strauss G, Meyer H, Burck G, and Truyen U (2001) Cowpox virus infection causing stillbirth in an Asian elephant (Elephas maximus). *Veterinary Record* 149:244-246.

Witchaya Tongtako C, P., Premalatha, B., Shafarin, M.S. and Azizah, D. (2013) Pentastomid (Armillifer moniliformis) infection Malaysian Blood Python (Python curtus) *Malaysian Journal of Veterinary Research* 4:51-54.

Wiwanitkit V (2005) A review of human sparganosis in Thailand. *International journal of infectious diseases* 9:312-316.

Wobeser G, Campbell GD, Dallaire A, and McBurney S (2009) Tularemia, plague, yersiniosis, and Tyzzer’s disease in wild rodents and lagomorphs in Canada: A review. *The Canadian Veterinary Journal* 50:1251-1256.

Wolfe ND, Switzer WM, Carr JK, Bhullar VB, Shanmugam V, Tamoufe U, Prosser AT, Torimiro JN, Wright A, Mpoudi-Ngole E, McCutchan FE, Birx DL, Folks TM, Burke DS, and Heneine W (2004) Naturally acquired simian retrovirus infections in central African hunters. *The Lancet* 363:932-937.

Woolfrey B, Quall C, and Lally R (1985) Pasteurella multocida in an infected tiger bite. *Archives of pathology & laboratory medicine* 109:744-746.

Xu H, Wang M, Zhang Z, Zou X, Gao Y, Liu X, Lu E, Pan B, Wu S, and Yu S (2004) An epidemiologic investigation on infection with severe acute respiratory syndrome coronavirus in wild animals traders in Guangzhou. *Zhonghua yu fang yi xue za zhi [Chinese journal of preventive medicine]* 38:81-83.

Yamaguchi T (1991) Present status of trichinellosis in Japan. *Southeast Asian Journal of Tropical Medicine Public Health* 22:295-301.

Yamamoto K, Chomel BB, Lowenstine LJ, Kikuchi Y, Phillips LG, Barr BC, Swift PK, Jones KR, Riley SP, and Kasten RW (1998) Bartonella henselae antibody prevalence in free-ranging and captive wild felids from California. *Journal of Wildlife Diseases* 34:56-63.

Yan J, Liu W, Gao S, Zhao N, Zhang Y, and Zhao B (2013) Isolation, Identification and the Preliminary Establishment of the Multiplex-PCR of Pathogens from Soft-shelled Turtle. *Journal of Hebei Normal University (Natural Science Edition)* 2:017.

Yang Z-Q, Wei C-G, Zen J-S, Song J-L, Zuo Y-X, He Y-S, Zhang H-F, Attwood SW, Chen X-W, Yang G-C, Zhou X, Quan X, Li C-Y, Han D, Liu A-W, and Lin P (2005) A taxonomic re-appraisal of Sarcocystis nesbitti (Protozoa: Sarcocystidae) from the monkey Macaca fascicularis in Yunnan, PR China. *Parasitology International* 54:75-81.

Yao MH, Wu F, and Tang LF (2008) Human pentastomiasis in China: case report and literature review. *Journal of Parasitology* 94:1295-1298.

Yapo Ette H, Fanton L, Adou Bryn K, Botti K, Koffi K, and Malicier D (2003) Human pentastomiasis discovered postmortem. *Forensic science international* 137:52-54.

Yasothai R, and Shamsudeen P (2014) A report on outbreak of anthrax in elephant. *Indian Journal of Field Veterinarians (The)* 9:81-81.

Ye F, Sheng Z-K, Li J-J, and Sheng J-F (2013) Severe pentastomisasis in children: A report of 2 cases *The Southeast Asian Journal of Tropical Medicine Public Health* 44:25-30.

Yob JM, Field H, Rashdi AM, Morrissy C, van der Heide B, Rota P, bin Adzhar A, White J, Daniels P, and Jamaluddin A (2001) Nipah virus infection in bats (order Chiroptera) in peninsular Malaysia. *Emerging infectious diseases* 7:439-441.

Yogasundram K, Shane SM, and Harrington KS (1989) Prevalence of Campylobacter jejuni in Selected Domestic and Wild Birds in Louisiana. *Avian Diseases* 33:664-667.

Zanella G, Durand B, Hars J, Moutou F, Garin-Bastuji B, Duvauchelle A, Fermé M, Karoui C, and Boschiroli ML (2008) Mycobacterium bovis in wildlife in France. *Journal of Wildlife Diseases* 44:99-108.

Zarnke RL (1983) Serologic survey for selected microbial pathogens in Alaskan wildlife. *Journal of Wildlife Diseases* 19:324-329.
